# ApoER2-Dab1 disruption as the origin of pTau-associated neurodegeneration in sporadic Alzheimer’s disease

**DOI:** 10.1186/s40478-023-01693-9

**Published:** 2023-12-13

**Authors:** Christopher E. Ramsden, Daisy Zamora, Mark S. Horowitz, Jahandar Jahanipour, Elizabeth Calzada, Xiufeng Li, Gregory S. Keyes, Helen C. Murray, Maurice A. Curtis, Richard M. Faull, Andrea Sedlock, Dragan Maric

**Affiliations:** 1 Lipid Peroxidation Unit, Laboratory of Clinical Investigation, National Institute on Aging, NIH (NIA/NIH), 251 Bayview Blvd., Baltimore, MD 21224 USA; 2 Intramural Program of the National Institute on Alcohol Abuse and Alcoholism, NIH, Bethesda, MD 20892 USA; 3 Flow and Imaging Cytometry Core Facility, National Institute of Neurological Disorders and Stroke, NIH, Bethesda, MD 20892 USA; 4 Department of Anatomy and Medical Imaging and Centre for Brain Research, Faculty of Medical and Health Science, University of Auckland, Private Bag, Auckland, 92019 New Zealand; 5 Laboratory of Functional and Molecular Imaging, National Institute of Neurological Disorders and Stroke, NIH, Bethesda, MD 20892 USA

**Keywords:** ApoER2, ApoE, Dab1, Alzheimer's disease, P85α, LIMK1, Tau, PSD95, Reelin, ApoJ

## Abstract

In sporadic Alzheimer’s disease (sAD) specific regions, layers and neurons accumulate hyperphosphorylated Tau (pTau) and degenerate early while others remain unaffected even in advanced disease. ApoER2-Dab1 signaling suppresses Tau phosphorylation as part of a four-arm pathway that regulates lipoprotein internalization and the integrity of actin, microtubules, and synapses; however, the role of this pathway in sAD pathogenesis is not fully understood. We previously showed that multiple ApoER2-Dab1 pathway components including ApoE, Reelin, ApoER2, Dab1, pP85α_Tyr607_, pLIMK1_Thr508_, pTau_Ser202/Thr205_ and pPSD95_Thr19_ accumulate together within entorhinal-hippocampal terminal zones in sAD, and proposed a unifying hypothesis wherein disruption of this pathway underlies multiple aspects of sAD pathogenesis. However, it is not yet known whether ApoER2-Dab1 disruption can help explain the origin(s) and early progression of pTau pathology in sAD. In the present study, we applied in situ hybridization and immunohistochemistry (IHC) to characterize ApoER2 expression and accumulation of ApoER2-Dab1 pathway components in five regions known to develop early pTau pathology in 64 rapidly autopsied cases spanning the clinicopathological spectrum of sAD. We found that (1) these selectively vulnerable neuron populations strongly express ApoER2; and (2) multiple ApoER2-Dab1 components representing all four arms of this pathway accumulate in abnormal neurons and neuritic plaques in mild cognitive impairment (MCI) and sAD cases and correlate with histological progression and cognitive deficits. Multiplex-IHC revealed that Dab1, pP85α_Tyr607_, pLIMK1_Thr508_, pTau_Ser202/Thr205_ and pPSD95_Thr19_ accumulate together within many of the same ApoER2-expressing neurons and in the immediate vicinity of ApoE/ApoJ-enriched extracellular plaques. Collective findings reveal that pTau is only one of many ApoER2-Dab1 pathway components that accumulate in multiple neuroanatomical sites in the earliest stages of sAD and provide support for the concept that ApoER2-Dab1 disruption drives pTau-associated neurodegeneration in human sAD.

**Supplementary Information:**

The online version contains supplementary material available at 10.1186/s40478-023-01693-9.

## Introduction

**Fig. 1:**
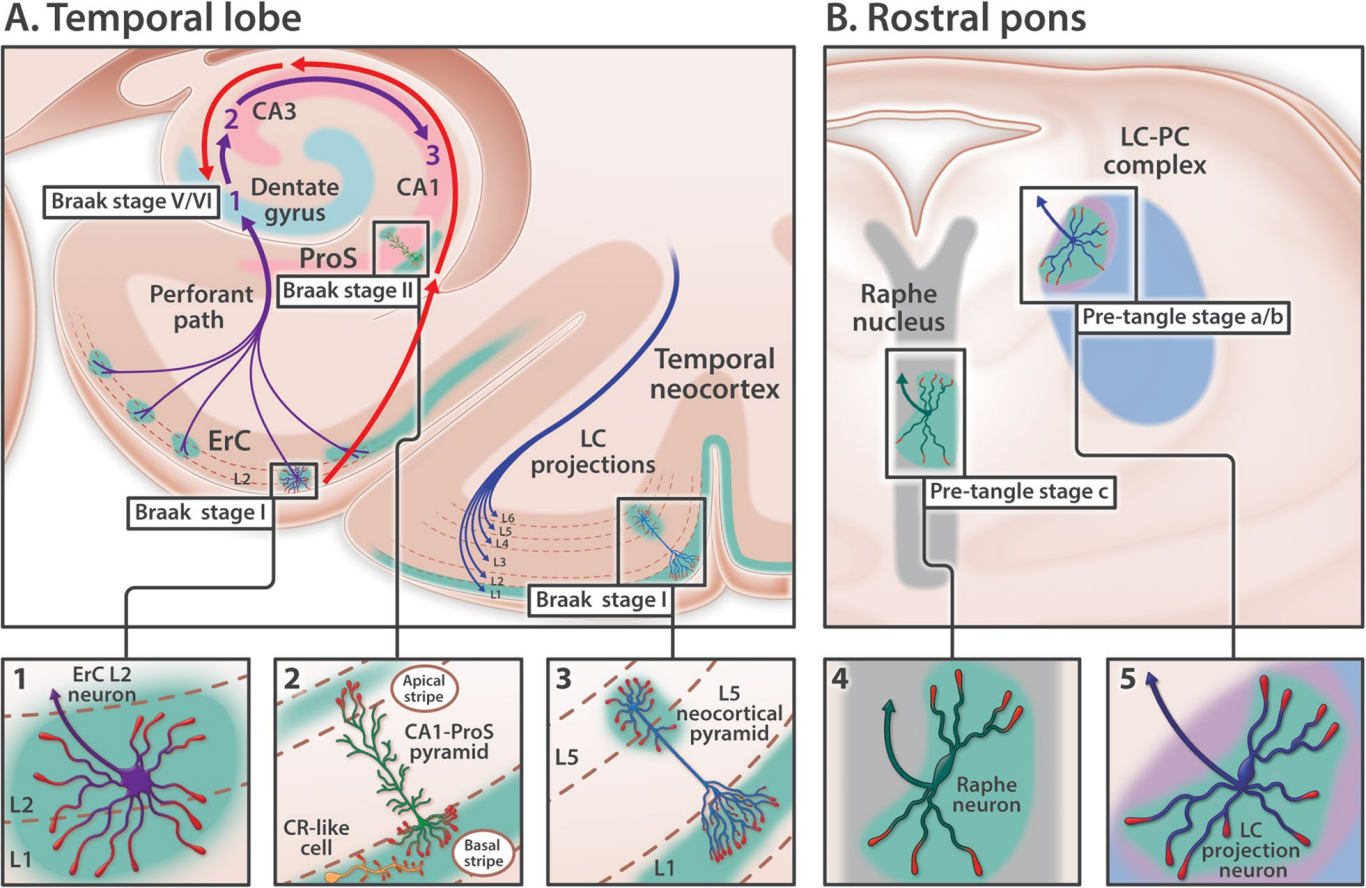
Anatomical sites of origin and ordered progression of NFT pathology in early sAD. Depiction of the stereotypical site(s) of origin and ordered sequence of progression of pTau pathology throughout the brain, focusing on five neuron populations in the temporal lobe (**A**) and rostral pons (**B**) that accumulate pTau in the earliest stages of sAD (i.e., Braak stages I–II and pre-tangle stages). The features that predispose these vulnerable neurons to NFT formation remain elusive. It has been suggested that Tau pathology spreads through synaptically connected neurons; however, discrepancies between connectivity and the distribution of pTau pathology appear to preclude a simple model of connectome-based spread in humans. In this paper we highlight these discrepancies and investigate an alternative hypothesis wherein high expression of a receptor for ApoE (teal shading) predisposes vulnerable neurons to pTau-associated neurodegeneration. **A** Temporal lobe: purple arrows denote the unidirectional tri-synaptic circuitry underlying memory formation. Red arrows trace the stereotypical progression of pTau pathology in the medial temporal lobe. In Braak stage I, pTau pathology is confined to ErC L2 projection neurons. In Braak stage II, pTau pathology progresses to include the basal lamina of the ProS-CA1 border region. In Braak stages III and IV, pTau pathology progresses throughout the cornu ammonis. Paradoxically, despite being the major synaptic recipient of ErC L2 neurons, dentate granule neurons (light blue arc) are spared from NFT pathology until late-stage sAD (Braak stages V/VI). This contrasting directionality of purple and red arrows indicates that NFT pathology progresses in a direction that is *opposite* to the synaptic connections underlying memory. Although often overlooked, pTau also accumulates within multiple distal dendritic tips of solitary L3 and L5 pyramids in the temporal neocortex very early in sAD (Braak stage I), while sparing neighboring neurons. **B** Rostral pons: pTau pathology classically begins in the LC–PC complex (pre-tangle stage a/b) before progressing to raphe nucleus (pre-tangle stage c) and ErC L2 (Braak stage I, see **A**). LC axonal projections (blue arrow in **B5**) innervate virtually the entire brain including all layers of temporal neocortex (depicted by blue axon terminals in L1–L6 of temporal cortex). Raphe neurons (green arrow in **B4**) also project widely throughout the brain. Since LC and raphe axons arborize over large areas and individual projection neurons classically innervate hundreds of neighboring target neurons, selective spread of pTau to ErC L2 neurons and solitary L3/L5 pyramids is not easily reconciled with connectome-based Tau spread

Sporadic Alzheimer’s disease (sAD) is not a global brain disease. Specific regions, layers and neurons accumulate hyperphosphorylated Tau (pTau) and degenerate early while others remain unaffected even in advanced disease [24, 25, 83, 84]. Despite decades of research, three key puzzles regarding the molecular basis of pTau-associated neurodegeneration remain unsolved. First, although neurofibrillary tangles (NFTs) have a remarkably consistent anatomical site of origin and sequence of progression throughout the brain (Fig. 1), specific molecular features that predispose vulnerable neurons to NFT formation remain elusive. Second, although pTau accumulates in four distinct lesions [NFTs, neuropil threads (NTs), neuritic plaques (NPs), and granulovacuolar degeneration bodies (GVDs)], the spatial and mechanistic connections between these lesions are not yet clear. Third, although Tau phosphorylation is well-established to destabilize microtubules, a causal role for pTau in other aspects of neurodegeneration (such as destabilization of the actin cytoskeleton and extracellular ApoE-Aβ plaque deposition) has not been established. While prion-like spread of Tau has been suggested to account for progression of pTau pathology throughout the brain, it does not explain how the first lesions develop [103] and it is not easily reconciled with the spatiotemporal distribution of NFTs in the early stages of sAD (Fig. 1). The identification of a single, shared mechanism that can (1) explain both the origin and stereotypical progression of NFT pathology; (2) provide a missing molecular link between the formation of NFTs, NTs, NPs, and GVDs; and (3) integrate these four pTau pathologies with other hallmark and emerging aspects of neurodegeneration, could have major implications for prevention and treatment of sAD.

### ApoER2-Dab1 disruption as the cause of pTau-associated neurodegeneration in sAD

**Fig. 2:**
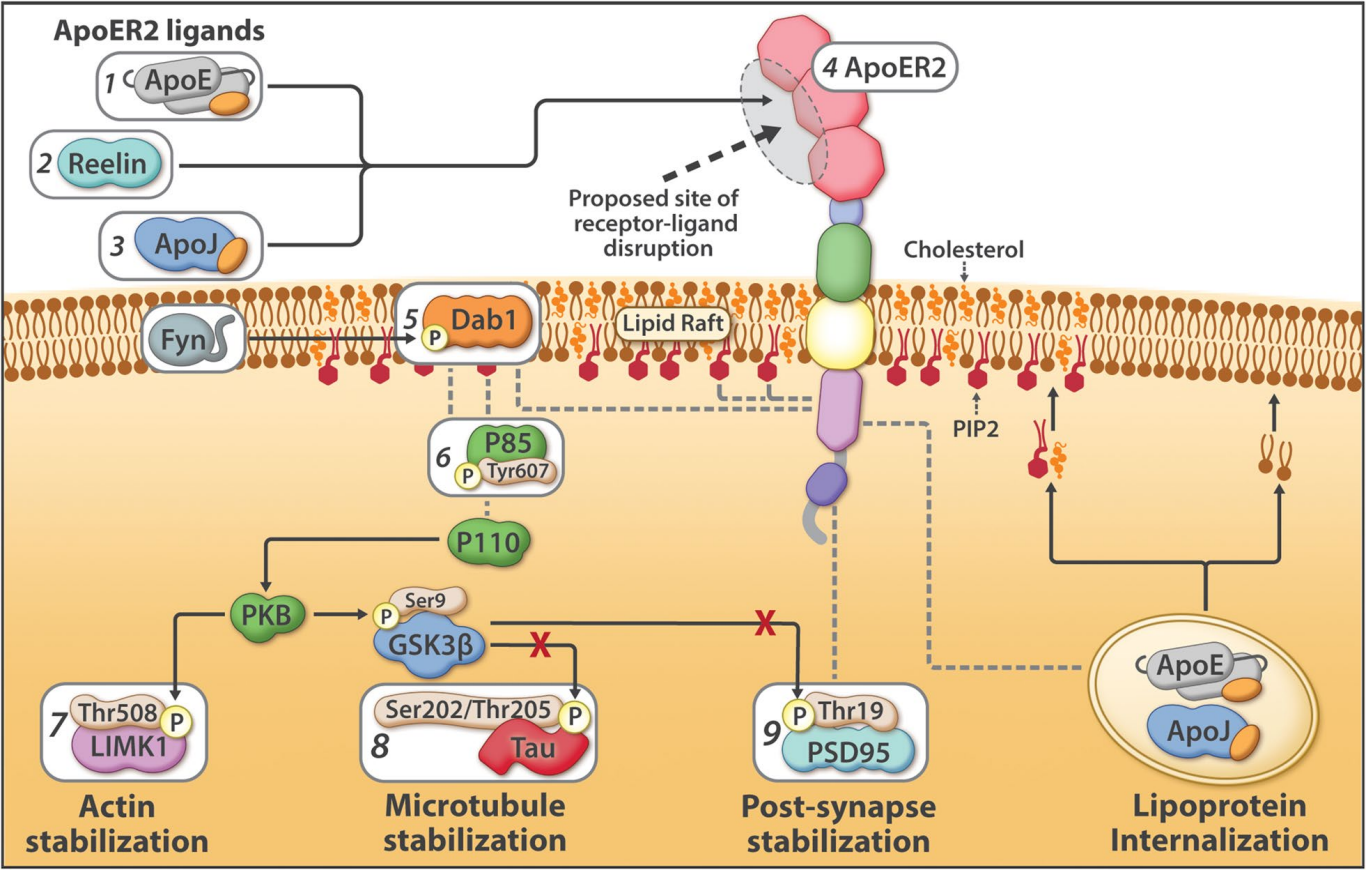
The ApoER2-Dab1 pathway and its proposed role in pTau-associated neurodegeneration in sAD. The ApoER2-Dab1 pathway suppresses Tau phosphorylation as part of a four-arm pathway that stabilizes actin, microtubules, and synapses, and delivers essential lipid cargo via receptor-mediated lipoprotein internalization. Nine core components of the ApoER2-Dab1 pathway that are a focus of the current paper are illustrated in the figure and described below. Under physiological conditions, binding of ligands (ApoE [1]), Reelin [2]), ApoJ [3]) to ApoER2 [4] triggers recruitment of Dab1 [5] and P85α [6] to lipid rafts, formation of ApoER2-Dab1-P85α signaling complexes, and phosphorylation, activation, and degradation of Dab1. Ensuing activation of the Dab1-P85α/PI3K cascade evokes phosphorylation of LIMK1 [7] to stabilize the actin cytoskeleton, and inhibition of GSK3β, which in turn inhibits phosphorylation of Tau [8] and PSD95 [9] to stabilize microtubules and postsynaptic receptor complexes, respectively. Disruption of this pathway at the level of ApoER2 (gray oval near ApoER2) is proposed to destabilize actin, microtubules, and synapses, to disrupt lipoprotein internalization, and to induce the co-accumulation of multiple ApoER2-Dab1 pathway components in sAD

Studies in model systems have shown that ApoER2-Dab1 signaling regulates memory, cognition, and neuronal integrity via a four-arm pathway (Fig. 2) [9, 17, 22, 35, 50, 51, 53, 57, 72, 93, 96, 98, 121, 124, 125, 128, 131, 136, 156]; however, the role of this pathway in the pathogenesis of human sAD has not been fully explored. The best-characterized arm of this pathway suppresses Tau phosphorylation [9, 50, 72, 125, 136] and thus disruption leads to pTau accumulation and destabilization of the microtubule cytoskeleton. The other three arms of this pathway regulate integrity of the actin cytoskeleton (via LIMK1 phosphorylation), synapse strength (via PSD95 phosphorylation), and neuronal delivery of cholesterol and specialized phospholipids (via lipoprotein internalization) (Fig. 2) (reviewed in [131]). Thus, disruption of this pathway at the level of ApoER2 could potentially trigger four core molecular derangements implicated in sAD pathogenesis, while inducing co-accumulation of multiple pathway components.

We previously showed that multiple ApoER2-Dab1 pathway components (including ApoE, Reelin, ApoER2, Dab1, pP85α_Tyr607_, pLIMK1_Thr508_, pTau_Ser202/Thr205_ and pPSD95_Thr19_) accumulate together in the terminal zones of the perforant path in sAD [131], and proposed a unifying hypothesis wherein disruption of this pathway drives multiple aspects of sAD pathogenesis. The importance of the ApoER2-Dab1 pathway in AD pathogenesis has since been bolstered by evidence that the *DAB1* gene locus is associated with AD risk in *APOE4* homozygotes [32], and that a gain-of-function variant in the *RELN* gene protects from familial AD in humans [109]. However, it is not yet known whether ApoER2-Dab1 disruption can help explain the origin(s) and stereotypical progression of pTau pathology in the early stages of sAD. Here, to gain insights into the potential role of ApoER2-Dab1 disruption in early pTau lesions we focus on five brain regions that are known to accumulate pTau in the very earliest stages of sAD (i.e., Braak stages I-II and pre-tangle stages) (Fig. 1). We hypothesized that (1) these vulnerable neuron populations strongly express ApoER2; and (2) multiple ApoER2-Dab1 pathway components accumulate in the vicinity of NTs, NFTs, NPs, and GVDs in mild cognitive impairment (MCI) and sAD cases in each affected region.

Using in situ hybridization (ISH), single-marker immunohistochemistry (IHC) and multiplex fluorescence IHC (MP-IHC) to examine human postmortem specimens spanning the clinicopathological spectrum of sAD we observed: (1) striking laminar and cellular distributions of ApoER2 with strong expression in neuron populations known to accumulate pTau early in sAD; and (2) accumulations of two ApoER2 ligands (ApoE, ApoJ) in extracellular plaques and five ApoER2 signaling partners (Dab1, pP85α_Tyr607_, pLIMK1_Thr508_, pPSD95_Thr19_, pTau_Ser202/Thr205_) within abnormal neurons in these same regions and layers in MCI and sAD cases. MP-IHC suggested that these five ApoER2 signaling partners co-accumulated within many of the same ApoER2-expressing, NT/NFT- and/or GVD-bearing neurons and in the vicinity of ApoE- and ApoJ-enriched NPs. These observations demonstrate co-accumulation of markers representing of all four arms of the ApoER2-Dab1 pathway in each of the sampled regions, layers, and neuron populations that are vulnerable to early NFT pathology. Collective findings reveal that pTau is only one of many ApoER2-Dab1 pathway components that accumulate in multiple neuroanatomical sites in the early stages of sAD and provide support for the concept that ApoER2-Dab1 disruption drives pTau-associated neurodegeneration in human sAD.

## Materials and methods

### Case selection and postmortem specimens

#### Banner Sun Health Research Institute Brain and Body Donation Program (BBDP)

Rapidly-autopsied, minimally-fixed specimens from 34 BBDP cases spanning the clinicopathological spectrum of sAD (Additional file 1: Tables S1 and S2) acquired from the BBDP at the Banner Sun Health Research Institute (http://www.brainandbodydonationprogram.org) [131] were used for the main analyses of ventral entorhinal cortex (ErC), prosubiculum (ProS)-CA1 border region, and temporal neocortex. Briefly, 6 μm-thick formalin-fixed, paraffin-embedded (FFPE) tissue sections containing (1) ErC at the level of the amygdala; (2) medial temporal lobe at the level of the body of the hippocampus; and (3) middle temporal gyrus were obtained from BBDP. BBDP employed a rapid, on-call autopsy team to achieve short postmortem interval (PMI) (mean of 3 h) to mitigate limitations due to tissue degradation. Standardized fixation procedures were employed using 1 cm^3^ tissue blocks fixed in 10% neutral buffered formalin for 48 h. All BBDP subjects provided written consent for study procedures, autopsy, and sharing of de-identified data prior to enrollment. The study and its consenting procedures were approved by the Western Institutional Review Board (IRB) of Puyallup, Washington. The Banner BBDP population has been extensively described. Briefly, most BBDP donors were enrolled as cognitively normal volunteers residing in retirement communities near Phoenix, Arizona. Following provision of informed consent, donors received standardized medical, neurological, and neuropsychological assessments during life. Neuropathological and cognitive endpoints captured by BBDP are extensive as described in previous publications [8, 16, 122, 158] and in the Additional file 1: pages 25–26. AD cases selected for this study had clinical dementia during life with pathologic diagnosis determined according to the NIA-Reagan criteria [152] using a high likelihood of AD threshold. MCI cases selected for this study were classified as MCI by BBDP based on a clinical diagnosis of MCI plus the presence of mild to moderate AD-type pathology that did not meet NIA-Reagan criteria for AD. Controls did not meet criteria for AD or MCI, although some degree of AD-type pathology was evident at autopsy in most controls. Key individual and summary characteristics of the BBDP cohort including PMI, Braak stage (0–VI), Thal phase (0–5), total amyloid plaques (0–15), neuritic plaque density (0–3), and *APOE* status are provided in Additional file 1: Tables S1a and S2a. The antemortem Mini-Mental Status Exam (MMSE, 0–30) test had the least missing data and was the main cognitive endpoint for the present study.

#### University of Auckland, New Zealand Neurological Foundation Human Brain Bank (UA-HBB) [120]

Minimally-fixed specimens from 10 sAD cases and 8 neurologically normal control cases (Additional file 1: Tables S1 and S2) that were acquired from the UA-HBB and the Human Anatomy Laboratory within the Department of Anatomy and Medical Imaging at the University of Auckland [120, 157] were used for the main analyses of upper pons [locus coeruleus (LC) plus raphe nucleus] and as a validation cohort for pathologies observed in the ErC and ProS-CA1 regions in BBDP specimens. Briefly, 5 μm-thick FFPE tissue sections including (1) transverse sections of the upper pons at the level of the LC and (2) medial temporal lobe specimens at the level of the body of the hippocampus were obtained from the UA-HBB team. Tissue was donated with informed consent from the family before brain removal; all procedures were approved by the University of Auckland Human Participants Ethics Committee (Ref: 011654). The right hemisphere of each brain was either perfusion or immersion fixed (depending on the location of the mortuary conducting the brain removal) in 15% formaldehyde in 0.1 M phosphate buffer for 48 h at room temperature. The upper pons block was paraffin-embedded in a transverse orientation as previously described [157]. The UA-HBB team carefully dissected the upper pons and each block was visually inspected to ensure presence of the LC and raphe nucleus. Presence of the LC and raphe nucleus was further confirmed by our team via hematoxylin–eosin and anti-tyrosine hydroxylase staining in serial sections. Neuropathological endpoints captured by UA-HBB have been extensively described [120, 157]. AD cases selected for this study had clinical dementia during life with pathologic diagnosis confirmed by independent pathological assessment. Neurologically normal control cases had no history of neurological abnormalities, and no substantive neuropathology was noted upon autopsy. Key individual and summary characteristics of the UA-HBB cohort including age, sex, PMI, Braak stage (0–VI), Thal phase (0–5), neuritic plaque density (0–3), and *APOE* status are provided in Additional file 1: Tables S1b and S2b.

#### University of Kentucky Alzheimer's Disease Research Center (UK-ADRC) Biobank

FFPE blocks containing the superior and middle temporal gyrus (SMTG) from 12 rapidly-autopsied cases spanning the clinicopathological spectrum of sAD were acquired through the UK-ADRC Biobank (Additional file 1: Tables S1 and S2). 6 μm-thick sections generated from these specimens were used solely for validation of pathologies observed in the BBDP and UA-HBB cohorts and were not used for quantitative analyses. The UK-ADRC team classified cases as AD, MCI, or control according to the NIA-Reagan criteria as previously described [140]. Standardized fixation procedures were employed with brain fixed in 10% neutral buffered formalin for approximately 1 month. UK-ADRC subjects provided written consent for study procedures, autopsy, and sharing of de-identified data prior to enrollment. The study and its consenting procedures were approved by the IRB (44009) of the University of Kentucky. Neuropathological and cognitive endpoints captured by UK-ADRC have been extensively described [140]. Individual and summary characteristics of the UK-ADRC cohort including age, sex, PMI, Braak stage (0–VI), Thal phase (0–5), neuritic plaque density (0–3), and *APOE* status are provided in Additional file 1: Tables S1c and S2c.

### Single-marker immunohistochemistry (IHC) and antibody validation

Single-marker IHC was performed by Histoserv (Gaithersburg, MD, USA) and in our NIA laboratory as previously described [131]. Briefly, combined blocks containing temporal cortex from sAD cases and non-AD controls were sectioned into 6 μm-thick coronal sections. Optimal immunostaining conditions were then empirically determined using an incremental heat induced epitope retrieval (HIER) method using 10 mM Na/Citrate pH6 and/or 10 mM TRIS/EDTA pH9 buffer that were heated at 70 °C for 10–40 min. Selected antibodies with suboptimal HIER treatment underwent additional retrieval rounds using formic acid (88%, 10 min) at room temperature. Secondary antibodies (Jackson ImmunoResearch) used for single IHC chromogenic staining were appropriately matched to the host class/subclass of the primary antibody. These combined sAD plus anatomically-matched control tissue blocks were used to generate negative controls (comparing staining results versus pre-immune serum and with the primary antibody omitted) and positive controls (comparing staining results in temporal or frontal cortex specimens from confirmed AD cases with known region-specific Aβ and tangle scores versus non-AD controls) as previously described. These empirically determined optimal conditions were then used to immunolabel sets of slides using IHC and MP-IHC (as described below). Briefly, for IHC, sections were first deparaffinized using standard Xylene/Ethanol/Rehydration protocol followed by antigen unmasking with 10–40 min HIER at 70 °C or formic acid for 10 min at room temperature, as described above.

Sources and technical specifications of reagents used in this study are provided in Additional file 1: Table S3. All antibodies used for IHC in this study have previously been used for IHC in human FFPE specimens and those targeting core ApoER2-Dab1 pathway components have been used specifically for IHC in human brain specimens in published manuscripts [131]. Data supporting the validation of these antibodies (i.e., Western blot, RNA–protein co-detection, multi-epitope immunolabeling) was included in the Additional file 1 of a published manuscript [131] and is summarized in Additional file 1: Table S3. For the present study, we performed additional automated Western immunoblotting experiments comparing antibody detection of lysates generated from HEK293T cells transfected to transiently overexpress human target proteins or empty vector transfected HEK293T cells (Additional file 1 pages 27–30).

### Multiplex fluorescence immunohistochemistry and in situ hybridization

MP-IHC and multiplex fluorescence in situ hybridization (MP-ISH) were performed on sections that were mounted on Leica Apex Superior adhesive slides (VWR, 10015-146) or UberFrost (InstrumeC) slides to prevent tissue loss. We completed one round of MP-ISH and up to 6 iterative rounds of sequential MP-IHC staining with select mRNA probes and antibody panels targeting the ApoER2-Dab1 pathway and/or classical sAD biomarkers, alongside classical cytoarchitectural biomarkers to map brain tissue parenchyma (see Additional file 1: Table S3). MP-ISH was carried out on a subset of sections using custom designed RNA probes and standard RNAScope Multiplex Fluorescent V2 hybridization kits (Advanced Cell Diagnostics, Inc.) per manufacturer’s instructions. Fluorophore-labelled mRNA/protein targets from each round of staining were imaged by multispectral epifluorescence microscopy followed by mRNA probe/antibody stripping and tissue antibody re-staining steps to repeat the cycle, as previously described [112, 131], each time using a different antibody panel. Briefly, for screening tissues by MP-ISH/MP-IHC, the sections were first deparaffinized using standard Xylene/Ethanol/Rehydration protocol followed by proteinase and/or HIER target unmasking steps in 10 mM Tris/EDTA buffer for 10 min using an 800 W microwave set at 100% power. Select sections were then processed for MP-ISH, as referenced above, and all sections to be sequentially processed for iterative MP-IHC screening were first incubated with Human BD Fc Blocking solution (BD Biosciences) to saturate endogenous Fc receptors and then in True Black Reagent (Biotium) to quench intrinsic tissue autofluorescence. Sections were then immunoreacted for 1 h at room temperature using cocktail mixture of immunocompatible antibody panels, with each antibody used at optimal staining concentration, as listed in Additional file 1: Table S3. This step was followed by washing off unbound primary antibodies in PBS supplemented with 1 mg/mL bovine serum albumin (BSA) and staining the sections using a 1 μg/mL cocktail mixture of the appropriately cross-adsorbed and host- and immunoglobulin class/subclass-specific secondary antibodies (purchased from either Thermo Fisher, Jackson ImmunoResearch, or Li-Cor Biosciences) conjugated to one of the following spectrally compatible fluorophores: Alexa Fluor 430, Alexa Fluor 488, Alexa Fluor 546, Alexa Fluor 594, Alexa Fluor 647, IRDye 600LT, or IRDye 800CW. After washing off excess secondary antibodies, sections were counterstained using 1 μg/mL DAPI (Thermo Fisher Scientific) for visualization of cell nuclei. Slides were then coverslipped using Immu-Mount medium (Thermo Fisher Scientific) and imaged using a multispectral epifluorescence microscope (see next section). After imaging, tissue-bound primary and secondary antibodies were both stripped off the slides after a 5-min incubation at room temperature in NewBlot Nitro 5X Stripping buffer (Li-Cor Biosciences) followed by a 1-min additional HIER step in Tris/EDTA buffer. The above processing cycle beginning with re-blocking of tissues in Human BD Fc Blocking solution was repeated and the same sections then incubated using an additional panel of antibodies of interest.

### Multiplex fluorescence immunohistochemistry image acquisition and computational reconstruction

Composite MP-IHC images were used to provide cytoarchitectural and pathological context for single-marker IHC images, which were used for quantitation. Images were acquired from MP-ISH and/or MP-IHC probed specimen sections using the Axio Imager.Z2 slide scanning epifluorescence microscope (Zeiss) equipped with a 20×/0.8 Plan-Apochromat (Phase-2) non-immersion objective (Zeiss), a high resolution ORCA-Flash4.0 sCMOS digital camera (Hamamatsu), a 200 W X-Cite 200DC broad band lamp source (Excelitas Technologies), and eight customized filter sets (Semrock) optimized to detect the following fluorophores: DAPI, Alexa Fluor 430, Alexa Fluor 488 (or Opal 520), Alexa Fluor 546 (or Opal 570), Alexa Fluor 594 (or Opal 620), Alexa Fluor 647, IRDye 680LT (or Opal 690), and IRDye 800CW. Image tiles (600 × 600 μm viewing area) were individually captured at 0.325 micron/pixel spatial resolution, and the tiles seamlessly stitched into whole specimen images using the ZEN 2 image acquisition and analysis software program (Zeiss), with an appropriate color table having been applied to each image channel to either match its emission spectrum or to set a distinguishing color balance. The RGB histogram of each image was adjusted using Zen software, resulting in optimized signal brightness and contrast, improved dynamic range, exposure correction, and gamma/luminosity-enhancement to reveal hidden/dim image details. The stitched images were exported as tif files, then computationally registered at the subpixel level using affine transformation and corrected for photobleaching, autofluorescence, non-uniform illumination shading, spectral bleed-through, and molecular colocalization artifacts. Images were exported as BigTIFF and imported into Adobe large document format upon which the images were linearly contrast-enhanced using the levels function, sharpened to reduce blurring using the sharpening filter, and pseudo-colored to enhance color contrast either to show colocalization or separated to display multiple markers in a single image, as previously described by Maric et al. [19, 120, 131].

### Regional annotation and quantitation

Single-marker IHC images were uploaded into HALO 3.5 image analysis software (Indica Labs, Corrales, NM). Boundaries of each anatomical region of interest (ErC, ProS-CA1 border region, gray matter of middle temporal gyrus, pontine LC plus raphe nucleus) were identified and annotated using a combination of anatomical landmarks (rhinal sulcus, dentate gyrus, temporal horn of lateral ventricle, pyramidal blades of the dentate gyrus, 4th ventricle) and cytoarchitectonic mapping as previously described [131]. MP-IHC images from serial sections with numerous cytoarchitectural markers labeled were used to assist with identification of landmarks and boundaries (i.e., white matter tracts, gray matter-white matter interfaces). Following alignment and registration of serial sections, the regional annotations were applied to each image. The flood fill annotation tool was used to help define boundaries and to limit the inclusion of edge artefacts. For most pathological markers, the HALO Area Quantification (AQ) v2.4.2 module was used to quantify the stain positive area as a percentage of each annotated region, as previously described [131]. Although prominent accumulations of Dab1 were evident in dystrophic neurites in the immediate vicinity of neuritic plaques, it is also expressed by many healthy neurons. To distinguish between pathological plaque-associated Dab1 and the Dab1 typically present in neurons, we used the HALO Object Colocalization (OC) v2.1.5 module with the classifier function enabled to selectively detect and quantify plaque-associated Dab1 objects per mm^2^ within each annotated region, as previously described [131]. Representative examples depicting applications of these two modules are provided in Additional file 1: Methods page 31.

### Statistical analysis

For each annotated region, between-group differences according to each marker were quantified using Kruskal–Wallis tests. Variable transformations (e.g., natural log) were used as necessary. A Spearman’s correlation coefficient between each immunohistochemical marker and each AD endpoint (Braak stage, Aβ plaque load, MMSE) was calculated. Graphs showing individual data points in each group and their relationships to sAD endpoints are provided in Figs. 4, 6, 9, and 10. In a sensitivity analyses accounting for the false discovery rate, we adjusted the *p* values using the two-stage linear step-up procedure described by Benjamini et al. [2, 14]. (Additional file 1: Table S4). Statistical analyses were conducted in Stata Release 17 [145].

## Results

### ApoER2 is strongly expressed in the same regions, layers and neuron populations that develop NFT pathology in the earliest stages of sAD

**Fig. 3:**
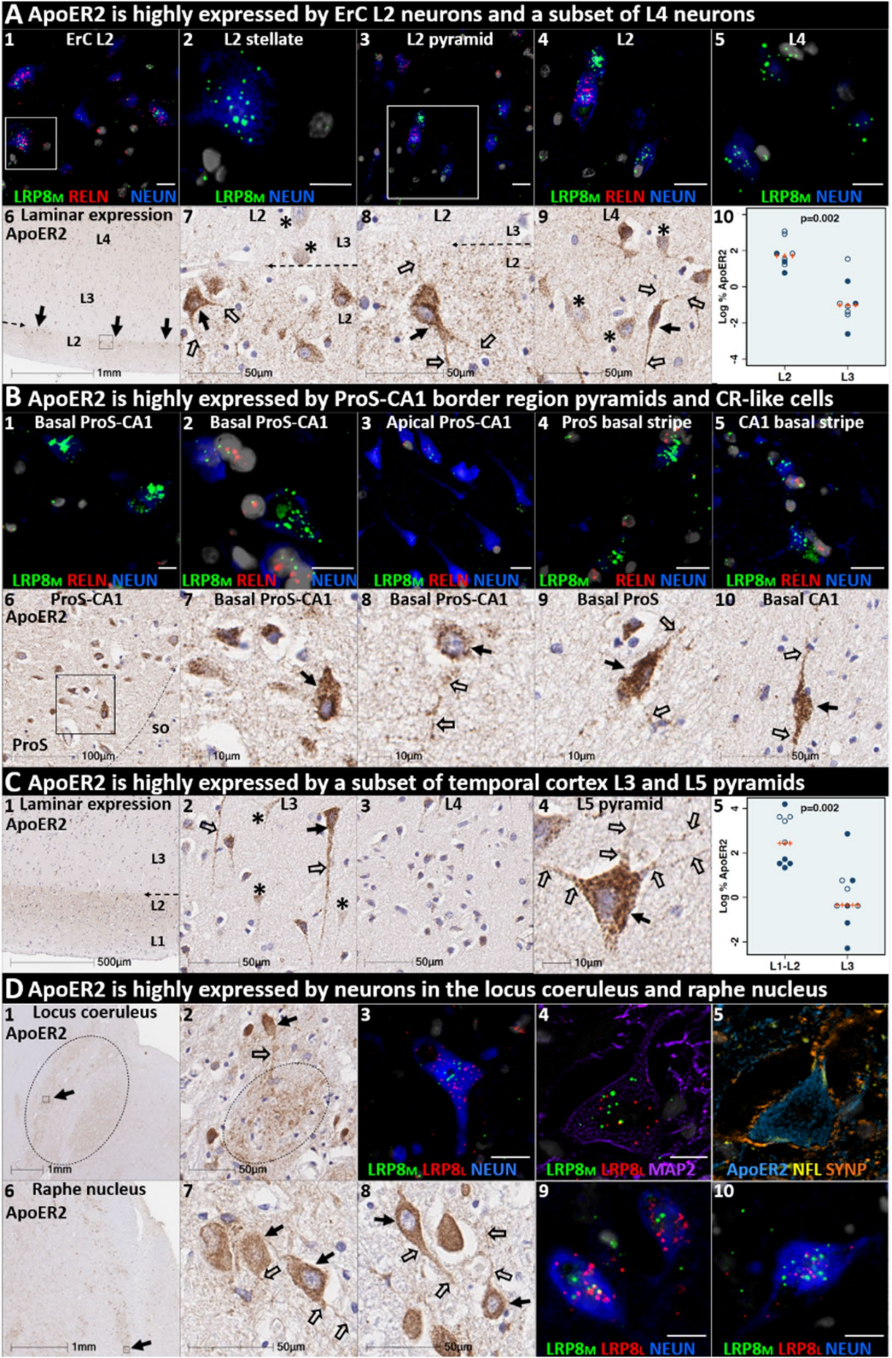
ApoER2 expression parallels regional, laminar, and cellular vulnerabilities to NFT pathology. Panels **A**–**D** are coronal sections of the ErC (**A**), ProS-CA1 border region (**B**), middle temporal gyrus (**C**), and transverse sections of the upper pons (**D**) from one representative middle-aged Braak stage I non-AD control. Soma and neuritic projections are demarcated by arrows and open arrows, respectively. Composite MP-IHC images were used to provide cytoarchitectural and pathological context for single-marker IHC images. **ErC** (**A**_**1–10**_): *LRP8* gene (LRP8_M_) (**A**_**1–5**_) and ApoER2 protein (**A**_**6–10**_) are strongly expressed by stellate-shaped Reelin-expressing (RELN) neurons in (**A**_**1–2,7–8**_), a subset of L2 pyramids (**A**_**3–4**_), and basal and apical dendritic tufts emanating from L2 neurons (**A**_**7–9**_). A visible laminar threshold (broken arrows in **A**_**6–8**_) for ApoER2 expression was observed near the L2–L3 border (**A**_**10**_); *p* value based on Wilcoxon rank-sum test. *LRP8* and ApoER2 are strongly expressed by a subset of L4 pyramids and surrounding neurites (**A**_**5**_,** A**_**9**_); adjacent L4 neurons with low ApoER2 expression are demarcated by an * in **A**_**9**_. *ProS-CA1 region* (**B**_**1–10**_): Strong *LRP8* (**B**_**1–5**_) and ApoER2 (**B**_**6–10**_) expression was observed in a subset of basal pyramids (**B**_**1–2**_) and neurons localized to the basal stripe (**B**_**4–5**_). Pyramids located in the middle and apical laminae tended to have lower *LRP8* and ApoER2 expression (**B**_**3**_). *Temporal neocortex* (**C**_**1–5**_): Strong ApoER2 expression in a subset of neocortical L3 and L5 pyramids, apical and basal dendritic projections (**C**_**3–4**_), and highly-ramified apical dendritic tufts (**C**_**1**_) located in the vicinity of L2. ApoER2 expression was low or absent in neighboring L3 pyramids (demarcated by * in **C**_**2**_) and most L4 stellate-shaped neurons (**C**_**3**_). A visible laminar threshold (broken arrow in **C**_**1**_) was observed near the L2–L3 border (**C**_**5**_); *p* value based on Wilcoxon rank-sum test. *Pontine LC and raphe nucleus* (**D**_**1–10**_): Strong ApoER2 protein (**D**_**1–2,6–8**_) and *LRP8* gene (LRP8_M_ and LRP8_L_) (**D**_**3–5,9–10**_) expression was observed in fusiform-shaped and multipolar LC neurons (**D**_**2–5**_) and in their MAP2-labeled dendritic tufts emanating into the peri-coeruleus region (oval in **D**_**2,**_ white arrows in **D**_**4–5**_). ApoER2 (**D**_**6–8**_) and *LRP8* (**D**_**9–10**_) are also strongly expressed in neurons and neuritic projections in the raphe nucleus. Scale bars in MP-IHC images = 5µm.

ApoER2 regulates hippocampal dendritic arborization and memory in rodent models [45, 93]. We recently demonstrated that human DG and hippocampal pyramidal neurons express ApoER2 [131], however, it is not yet known whether neurons that develop NFT pathology in the earliest stages of sAD express ApoER2. In the present study, IHC and MP-ISH/IHC revealed that regional, laminar, cellular and subcellular (dendritic) distributions of ApoER2 and *LRP8* expression parallel well-established selective vulnerability to develop NFT pathology—with strong expression in ErC L2 neurons, the ProS-CA1 border region, a subset of L5 and L3 neocortical pyramids, and pontine LC and raphe nuclei neurons—and lower or absent expression in neuron populations that are spared from NFT pathology (Fig. 3, Additional file 1: Ext Fig S3.1). In ErC L2, both Reelin-expressing stellate projection neurons and pyramidal neurons are particularly vulnerable to pTau accumulation and are affected in Braak stage I [24, 25, 27, 95], with pTau pathology originating in distal dendrites, followed by cell bodies and finally axons [25]. L4 pyramids are affected next in Braak stage II, while L3 neurons are not affected until later in the disease process [24, 25, 27]. ISH and IHC in the ErC region revealed striking laminar and cellular *LRP8* and ApoER2 expression patterns, with strong signals observed in the soma of Reelin-expressing stellate-shaped ErC L2 neurons and in a subset of ErC L2 pyramids lacking Reelin expression, and their basal and apical dendritic projections emanating into L1-L2 border region (Fig. 3, Additional file 1: Ext Fig. S3.1). ApoER2 protein expression was higher in L2 than L3, resulting in a visible laminar termination threshold at the L2–L3 border. Strong ApoER2 expression was also observed in a subset of ErC L4 pyramids but was minimal or absent in a subset of neighboring L4 neurons (Fig. 3A, Additional file 1: Ext Fig. S3.1).

The basal stripe of the ProS-CA1 border region begins to accumulate pTau in Braak stage II and is the first subregion of the hippocampal formation to develop NFT pathology [24, 27]. ISH and IHC in the ProS-CA1 border region revealed prominent laminar and cellular *LRP8* and ApoER2 expression patterns with moderate-to-strong expression in basal pyramids and non-pyramidal neurons and neurites localized to the basal stripe (Fig. 3B). Pyramids located within the middle and apical layers tended to have lower *LRP8* expression (Fig. 3B).

pTau accumulation is evident within multiple distal dendritic tips emanating from solitary temporal L5 and L3 pyramids very early in sAD (Braak stage I) [29]. The observation that pTau pathology spares neighboring neurons is an enduring puzzle that is not readily explained by prion-like Tau spread [27, 29, 30]. Spiny stellate cells in L4 do not develop pTau pathology even in severe sAD [28, 29]. In temporal and frontal neocortex (Fig. 3C, Additional file 1: Ext Fig. S3.1), we observed striking layer- and cell-specific distributions of ApoER2 with the strongest expression in the perikarya of subset of L5 and L2/L3 pyramids and their basal dendrites, and in the distal portions of their highly branched apical dendritic tufts in the L1–L2 border region (Fig. 3C, Additional file 1: Ext Fig. S3.1) ApoER2 protein expression was higher in L1–L2 than L3, resulting in a visible laminar termination threshold near the L2–L3 border. Despite this high expression in a subset of L5 neocortical pyramids and their basal and apical dendrites (Fig. 3C, Additional file 1: Ext Fig. S3.1), ApoER2 expression was minimal or absent in a subset of neighboring L5 pyramids and in most L4 neurons.

LC and raphe neurons in the upper pons accumulate pTau very early in the sAD process (pre-tangle stages a/b and c, respectively) [31]. In LC and raphe nuclei and scattered clusters of multipolar neurons located between both structures, IHC and MP-ISH/IHC revealed strong *LRP8* and ApoER2 expression in neuronal soma and high ApoER2 expression in neuritic projections (Fig. 3D). Strong ApoER2 protein expression was observed within highly-branched projections emanating from LC neurons to the peri-coeruleus. MP-IHC revealed that ApoER2 expression in the LC had substantial overlap with MAP2-labeled dendritic arbors but minimal overlap with the axonal and pre-synaptic terminal markers NFL and synaptophysin, respectively.

The expression of two other ApoE receptors (LRP1 and VLDLR) was less restricted than ApoER2 and neither closely matched the laminar and cellular distribution of NFT pathology. LRP1 was expressed by glia and neurons, with prominent signals in glia surrounding NPs (Additional file 1: Ext Fig. S3.2). Unlike ApoER2, VLDLR was strongly and ubiquitously expressed by neurons in all neocortical layers including neocortical L4 stellate neurons (Additional file 1: Ext Fig. S3.2) that are known to be resistant to NFT pathology [28, 29].

### Pathological accumulation of ApoER2-Dab1 pathway components in ErC L2

**Fig. 4:**
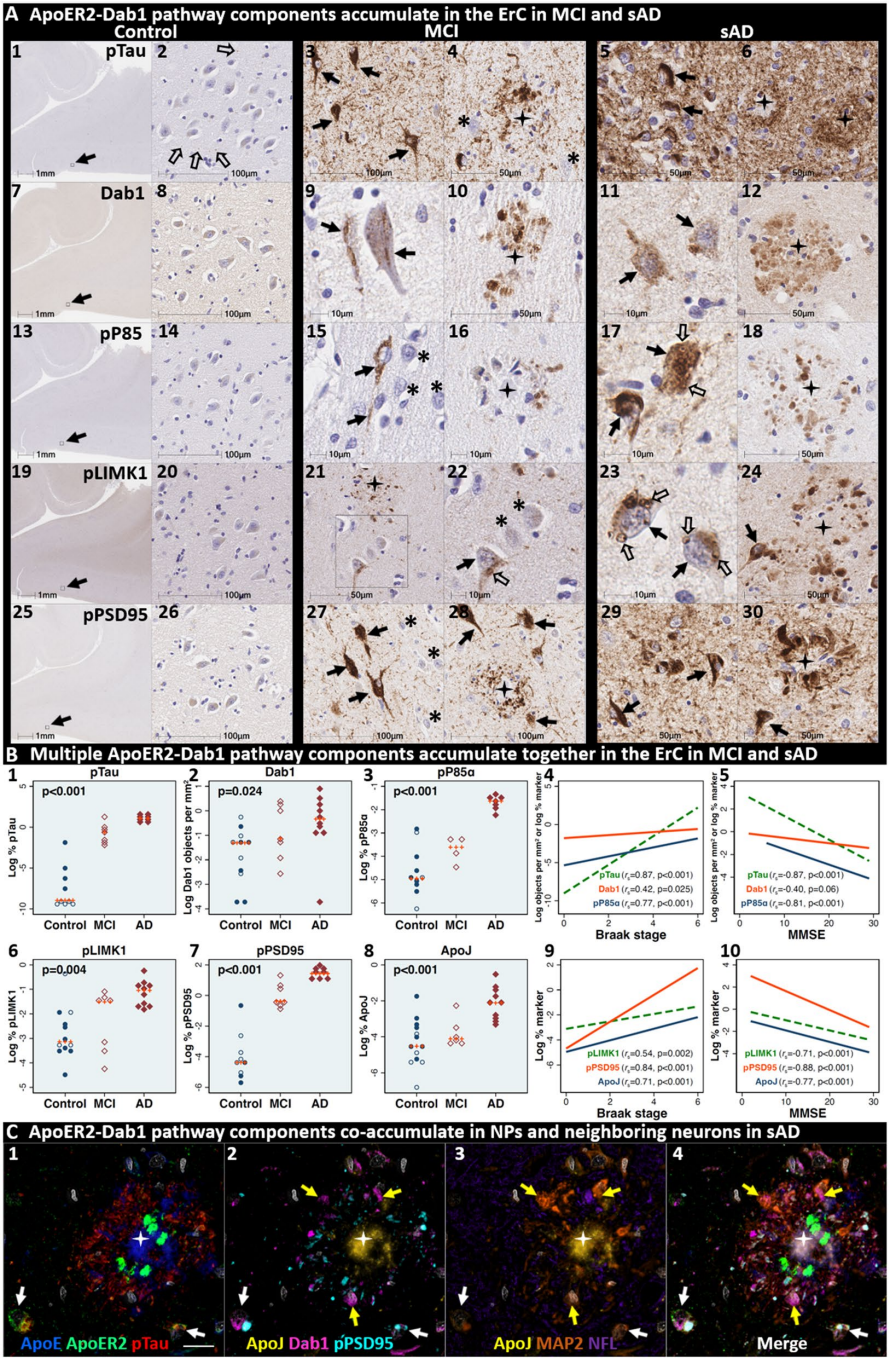
Multiple ApoER2-Dab1 pathway components accumulate together with pTau in ErC in MCI and sAD cases. **A** Serial coronal sections of the entorhinal region from non-AD control (Braak stage I), MCI (Braak stage III) and sAD (Braak stage VI) cases were probed with antibodies targeting ApoER2-Dab1 pathway components (see Additional file 1: Table S3). Zoomed-out images of the non-sAD control are provided (left column in **A**) to illustrate the lack of positive IHC staining throughout the whole ErC section, with only modest pTau_Ser202/Thr205_ accumulation (open arrows in **A**_**2**_) and no overt accumulation of pP85α_Tyr607_, pLIMK1_Thr508_, or pPSD95_Thr19_. Dab1 expression was confined to the soma of a subset of neurons. By contrast, in MCI (middle column in **A**) and especially sAD (right column in **A**), prominent accumulations of pTau_Ser202/Thr205_, Dab1, pP85α_Tyr607_, pLIMK1_Thr508_, and pPSD95_Thr19_ were observed within abnormal neurons (solid arrows) and in the vicinity of NPs (black stars). Examples of neighboring neurons with little or no evidence of ApoER2-Dab1 component accumulation are designated with * (**A**_**4,15,22,27**_). Open arrows in **A**_**17**_ and **A**_**22–23**_ designate granulovacuolar accumulations of pP85α_Tyr607_ and pLIMK1_Thr508_, respectively. **B** The expression of multiple neuronal ApoER2-Dab1 pathway components increased across the clinicopathological spectrum of sAD (**B**_**1–3,6–8**_) and correlated with Braak stage (**B**_**4,9**_) and cognitive deficits (**B**_**9,10**_). For dot plots, open and closed blue circles indicate young controls and age-matched controls; open and closed red diamonds indicate MCI cases and sAD cases, respectively. *p* values are from Kruskal–Wallis tests. Fitted line plots show Spearman’s rank correlation coefficients and *p* values. **C** MP-IHC suggested that Dab1, pTau_Ser202/Thr205_, and pPSD95_Thr19_ accumulate together within ApoER2-expressing neurons (white arrows in **C**_**1–4**_) and that Dab1 accumulated within MAP2-labeled dystrophic dendrites (yellow arrows in **C**_**1–4**_) in the vicinity of ApoE and ApoJ-enriched extracellular plaques (white star). Scale bars in MP-IHC images = 5 µm

We next sought to determine if ApoER2-Dab1 pathway components accumulate in the ErC and whether such accumulations increase across the clinicopathological spectrum of sAD. Single-target IHC using serial sections revealed that seven ApoER2-Dab1 pathway components—Dab1, pP85α_Tyr607_, pLIMK1_Thr508_, pTau_Ser202/Thr205_, pPSD95_Thr19_, ApoJ and ApoE—accumulated in abnormal neurons, and in proximity to NPs, were higher in MCI and sAD cases than controls and positively correlated with histological progression or antemortem cognitive deficits (Fig. 4, Additional file 1: Ext Fig. S4.1). ApoER2-Dab1 components exhibited different distributions and morphologies (Fig. 4A). pTau prominently accumulated as hallmark NTs and NFTs, and in the neuritic components of NPs. Dab1 accumulated within intraneuronal inclusions and large globular complexes. pP85α_Tyr607_ and pLIMK1_Thr508_ accumulated in intraneuronal vacuolar structures reminiscent of GVDs and to a lesser extent in globular structures consistent with plaque-associated dystrophic neurites. pPSD95_Thr19_ accumulated in intraneuronal vacuolar structures, small punctae surrounding affected neurons, and globular structures consistent with plaque-associated dystrophic neurites. ApoE and ApoJ accumulated primarily within extracellular plaques. MP-IHC suggested that Dab1, pP85α_Tyr607_, pLIMK1_Thr508_, and pPSD95_Thr19_ accumulated together with pTau_Ser202/Thr205_ within MAP2 and ApoER2-labeled dystrophic dendrites and perikarya of nearby ApoER2-expressing abnormal neurons. Extracellular lipoprotein deposition was also evident with ApoE and ApoJ co-localized in the central core of many of the same NPs (Fig. 4C).

**Fig. 5:**
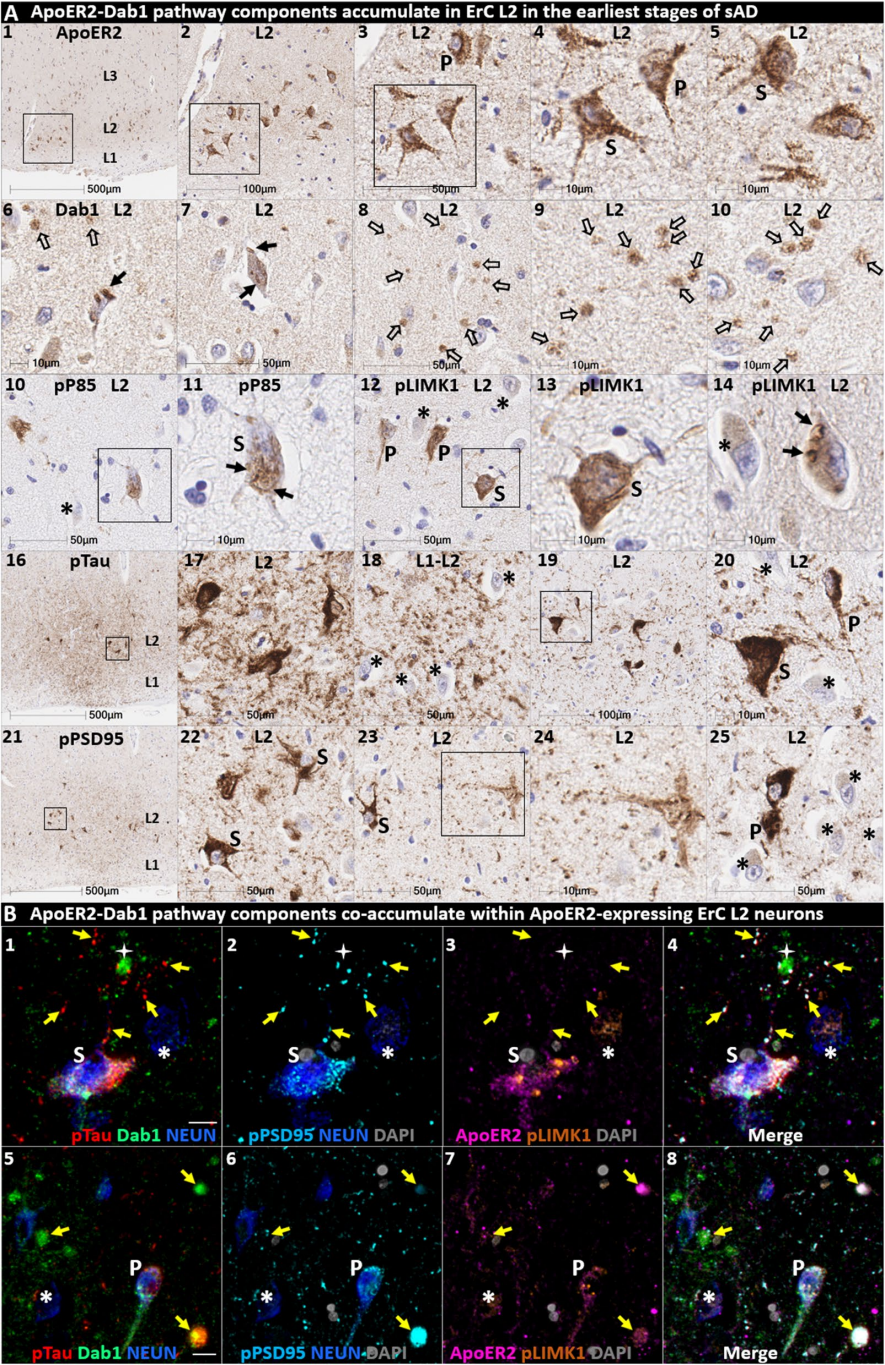
Multiple ApoER2-Dab1 components accumulate in the same ApoER2-expressing ErC L2 neurons in Braak stage I. Serial coronal sections of the ErC from one non-AD control case in an initial stage of NFT pathology (Braak stage I, Thal phase I, MMSE 27/30) were probed with antibodies targeting ApoER2-Dab1 pathway components. Single-target IHC revealed that both stellate-shaped (designated S) and pyramid-shaped (designated P) neurons in ErC L2 strongly express ApoER2 (**A**_**1–5**_), and that multiple ApoER2-Dab1 pathway components including Dab1 (**A**_**6–10**_), pP85α_Tyr607_ (**A**_**10–11**_), pLIMK1_Thr508_ (**A**_**12–15**_), and pPSD95_Thr19_ (**A**_**21–25**_) accumulated together with pTau_Ser202/Thr205_ (**A**_**16–20**_). Dab1 accumulated within the soma of a subset of ErC L2 neurons (solid arrows) and adjacent globular grape-like structures (open arrows). pP85α_Tyr607_ accumulated within the soma of a subset of stellate-shaped neurons (S in **A**_**11**_). pLIMK1_Thr508_ accumulated within a subset of stellate-shaped (S in **A**_**12–13**_) and pyramidal neurons (P in **A**_**12**_). Granulovacuolar pLIMK1_Thr508_ accumulations are designated with solid arrows in **A**_**14**_. pTau_Ser202/Thr205_ accumulated within a subset of NFT-bearing stellate-shaped and pyramidal neurons, and as NTs in the adjacent neuropil. pPSD95_Thr19_ accumulated in the soma of affected stellate-shaped and pyramidal neurons and as discrete punctae in adjacent neuropil. Neighboring neurons with little or no evident accumulations of ApoER2-Dab1 components are designated with an * in **A**_**10–25**_. **B** MP-IHC suggested that pTau_Ser202/Thr205_, Dab1, pLIMK1_Thr508_, and pPSD95_Thr19_ accumulate together within the same ApoER2-expressing stellate-shaped neurons (designated **S** in **B**_**1–5**_) and pyramidal neurons (designated **P** in **A**_**5–8**_), and adjacent dystrophic neurites (yellow arrows) that may emanate from these abnormal ApoER2 and Dab1-expressing ErC L2 neurons. Neighboring NEUN-labeled neurons with little or no ApoER2-Dab1 components evident in the soma are designated with an *. Scale bars in MP-IHC images = 5 µm

We next searched for evidence for ApoER2-Dab1 pathway disruption in the earliest stage of pTau pathology before clinical manifestations are apparent. Using IHC and MP-IHC for deep phenotyping of ErC in a Braak stage I/Thal phase I non-AD case (Fig. 5), we observed that Dab1, pLIMK1_Thr508_, and pPSD95_Thr19_ tend to accumulate together with pTau_Ser202/Thr205_ within the soma of the same L2 ApoER2-expressing stellate-shaped neurons and pyramidal neurons (Fig. 5). Despite this selective vulnerability of their neighbors, a subset of adjacent neurons appears to be spared from pathological accumulation of pTau and other ApoER2-Dab1 components (designated with * in Fig. 5). pTau_Ser202/Thr205_ and pPSD95_Thr19_ also accumulated within adjacent ApoER2-enriched neuritic projections. Dab1 accumulated within globular structures that co-localized with MAP2-labeled dystrophic dendrites, and to a lesser extent with NFL-labeled axons (Additional file 1: Ext Fig. S5.1). Unlike MCI and sAD cases, these intra-neuronal and neuritic ApoER2-Dab1 pathway component accumulations were accompanied by only subtle extracellular ApoE and ApoJ deposits (Additional file 1: Ext Fig. S4.1).

### Pathological accumulation of ApoER2-Dab1 pathway components in the ProS-CA1 border region

**Fig. 6:**
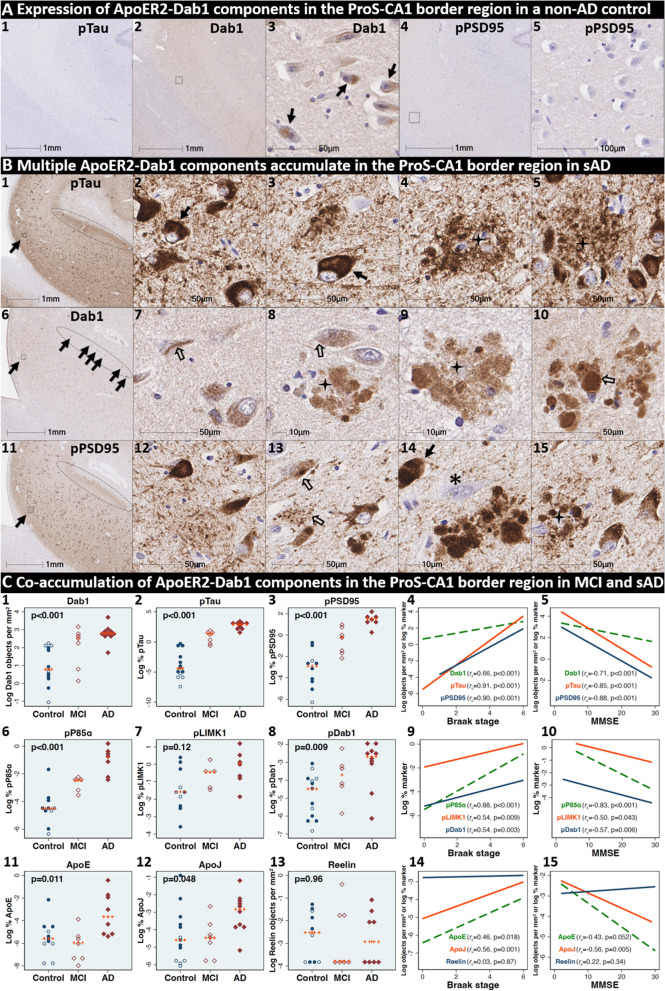
Co-accumulation of ApoER2-Dab1 pathway components in ProS-CA1 border region in sAD. (**A**) Serial coronal sections of the ProS-CA1 region from representative non-AD control (Braak stage I) and sAD (Braak stage IV) cases were probed with antibodies targeting ApoER2-Dab1 pathway components (see Additional file 1: Table S3). In the non-sAD control (**A**), single-target IHC revealed little or no expression of pTau_Ser202/Thr205_ and pPSD95_Thr19_, with modest Dab1 expression confined to a subset of neurons. In sAD cases, pTau_Ser202/Thr205_ accumulated within NTs, NFTs, and the neuritic components of NPs (**B**_**1–5**_). Dab1 accumulated within abnormal neurons (**B**_**7–8**_) and in plaque-associated clusters of globular neurites (**B**_**8–10**_). pPSD95_Thr19_ accumulated within abnormal neurons, neuritic components of NPs, and in discrete punctae within the neuropil. All three components accumulated within the apical stripe region (arrows in **B**_**1,6&11**_), which harbors the terminal apical dendritic projections emanating from basal ProS-CA1 pyramidal neurons. **C** Expression of Dab1, pTau_Ser202/Thr205_, pPSD95_Thr19_, pP85α_Tyr607_, pLIMK1_Thr508_, pDab1_Tyr220_, ApoE, and ApoJ increased across the clinicopathological spectrum of sAD and correlated with Braak stage and cognitive deficits (**C**_**1–15**_). For dot plots, open and closed blue circles indicate young controls and age-matched controls; open and closed red diamonds indicate MCI cases and sAD cases, respectively. *p* values are from Kruskal–Wallis tests. Fitted line plots show Spearman’s rank correlation coefficients and *p* values. IHC images for pP85α_Tyr607_, pLIMK1_Thr508_, pDab1_Tyr220_, and ApoJ are provided in Additional file 1: Ext Fig. S6.1

**Fig. 7:**
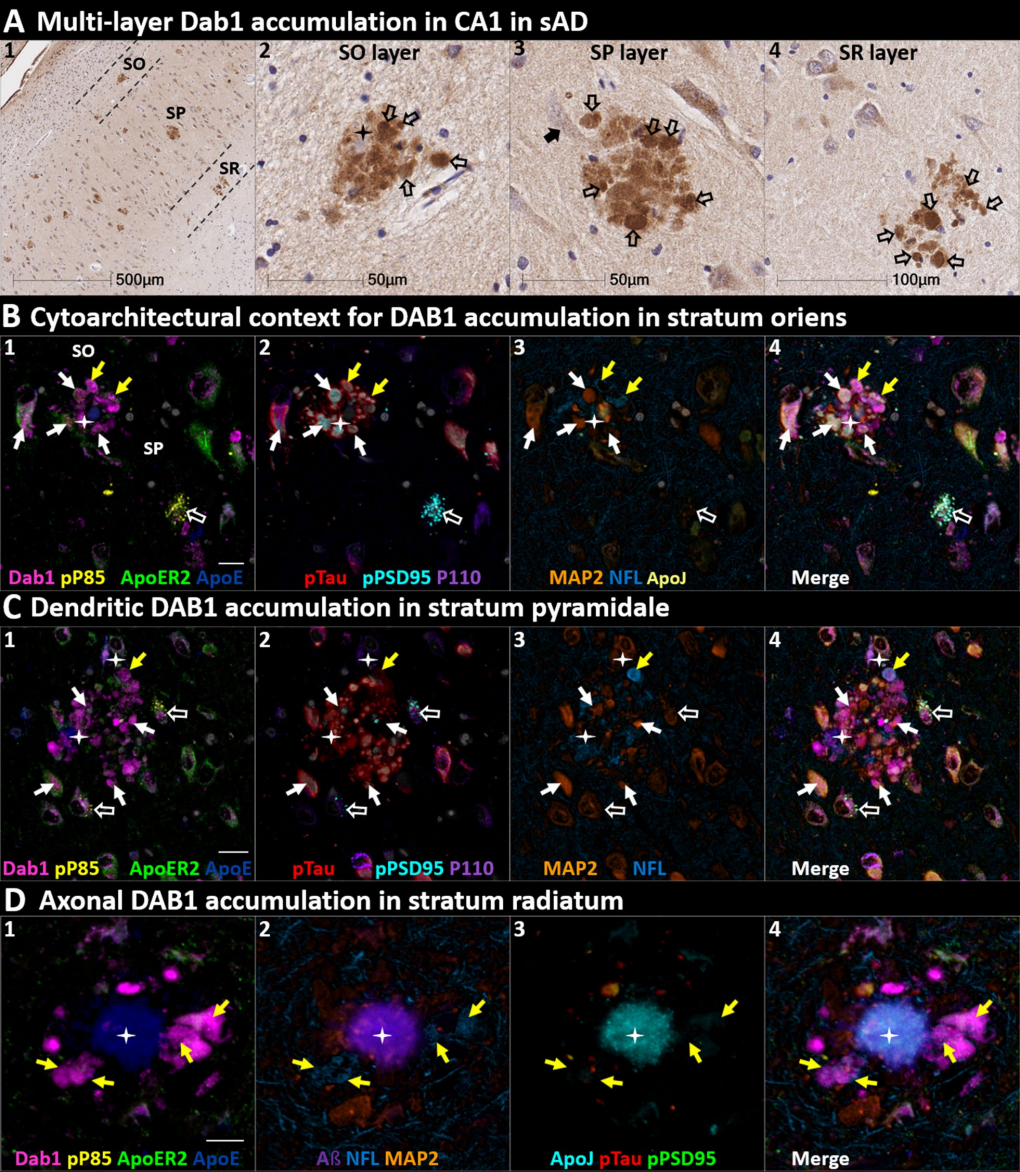
DAB1 accumulates in dystrophic dendrites and axons in the CA1 region in sAD. Coronal sections of a representative (Braak stage IV, Thal phase 5) sAD case were probed with antibodies targeting ApoER2-Dab1 pathway components (see Additional file 1: Table S3). A Single-target IHC revealed that Dab1 accumulates as grape-like clusters in three layers of CA1 (stratum oriens [SO], stratum pyramidale [SP], and stratum radiatum [SR]). The globular Dab1 accumulations (open arrows in **A**_**2–4**_) are reminiscent of dystrophic neurites, however it is not yet clear if they are of dendritic or axonal origin. Dab1 clusters in the SP layer appear to emanate from a single neighboring neuron (black arrow in **A**_**3**_). A presumptive neighboring neuronal source of Dab1 complexes is not evident in the SO and SR. **B** MP-IHC suggested that in the SO layer Dab1 accumulates within both NFL-labeled axons (yellow arrows) and MAP2-labeled dendrites (white arrows) surrounding an ApoE enriched plaque (star). One neighboring ApoER2-expressing neuron at the SO-SP border had prominent intraneuronal accumulation of Dab1 and pTau (white arrows). Another nearby ApoER2 and Dab1-expressing neuron in the SP layer had prominent granulovacuolar co-accumulation of pP85α_Tyr607_ and pPSD95_Thr19_ (open white arrows), with little or no pTau_Ser202/Thr205_ evident. **C** MP-IHC in the SP layer suggested that Dab1 accumulates primarily within MAP2-labeled dendrites (white arrows in **C**_**1–4**_) and soma of neighboring neurons in the vicinity of extracellular ApoE (white stars). However, one Dab1-expressing globular structure co-localized with NFL (yellow arrow). Dab1 accumulated together with pTau and pPSD95_Thr19_ in several neighboring ApoER2-expressing neurons (white arrows in **C**_**1–4**_). Two nearby ApoER2 and Dab1-expressing neurons had prominent granulovacuolar co-accumulation of pP85α_Tyr607_ and pPSD95_Thr19_ (open white arrows), with little or no pTau_Ser202/Thr205_ evident. **D** MP-IHC in the SR layer suggested that Dab1 accumulated primarily within dystrophic NFL-labeled axons (yellow arrows in **D**_**1–4**_) surrounding ApoE, ApoJ and Aβ-enriched extracellular plaques (white star). Unlike the SP layer, Dab1 accumulation was not evident within MAP2-labeled dendrites. Scale bars in MP-IHC images = 10 µm

**Fig. 8:**
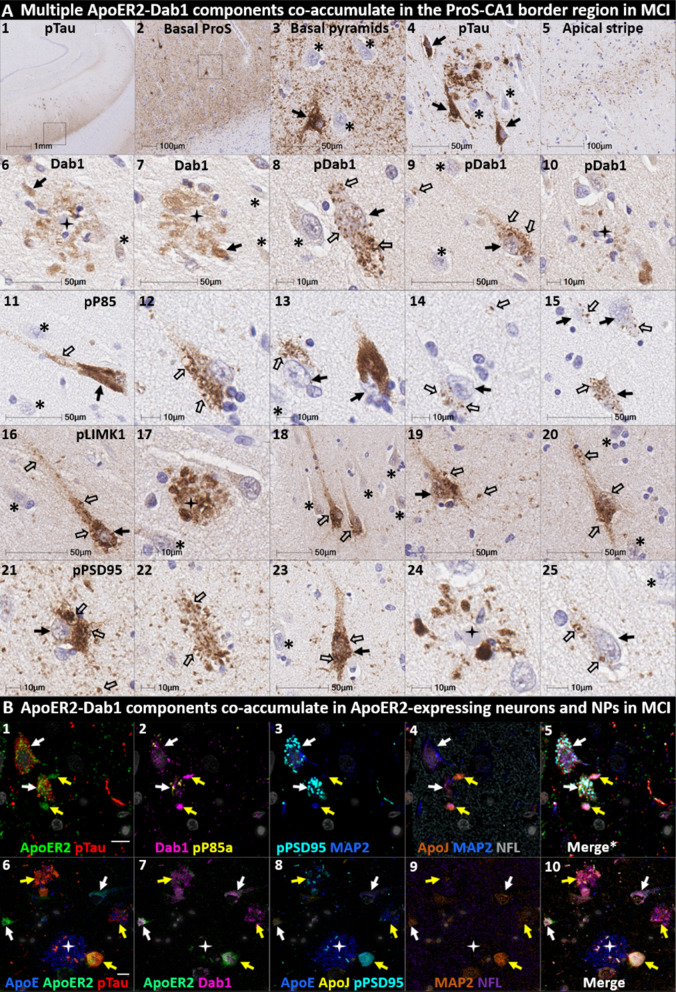
Multiple ApoER2-Dab1 components accumulate in ApoER2-expressing neurons and NPs in the ProS-CA1 border region in early MCI. Serial coronal sections of the ProS-CA1 border region in a representative early MCI case (Braak stage III, MMSE 28/30) were probed with antibodies targeting ApoER2-Dab1 pathway components. Single-target IHC revealed regional co-accumulation of multiple ApoER2-Dab1 pathway markers including pTau_Ser202/Thr205_ (**A**_**1–5**_), Dab1 (**A**_**6–7**_), pDab1_Y220_ (**A**_**8–10**_), pP85α_Tyr607_ (**A**_**11–15**_), pLIMK1_Thr508_ (**A**_**16–20**_), and pPSD95_Thr19_ (**A**_**20–55**_). Affected neurons, GVD-like structures, and the apparent locations of extracellular plaques are designated with solid arrows, open arrows, and stars, respectively. Neighboring neurons with no evident accumulations of ApoER2-Dab1 components are designated with an * in **A**_**10–25**_. MP-IHC suggested that pTau_Ser202/Thr205_, Dab1, pP85α_Tyr607_, and pPSD95_Thr19_ accumulate together within a subset of ApoER2-expressing basal pyramidal neurons (open arrows in **B**_**1–5**_), and within MAP2- and ApoER2-immunoreactive globular (yellow arrows in **B**_**1–10**_) or thread-like (white arrows in **B**_**1–5**_) dystrophic dendrites in the vicinity of affected neurons and ApoE-enriched NPs (white star in **B**_**6–10**_). NFL is not included in merged images (**B**_**5**_, **B**_**10**_) to enhance visualization. Scale bars in MP-IHC images = 10 µm

We next sought to determine if ApoER2-Dab1 components accumulate in the ProS-CA1 border region and whether such accumulations increase across the clinicopathological spectrum of sAD. Single-target IHC revealed that eight ApoER2-Dab1 pathway markers (Dab1, pP85α_Tyr607_, pLIMK1_Thr508_, pTau, pPSD95_Thr19_, pDab1_Tyr220_, ApoE, ApoJ) accumulated within abnormal neurons or NPs, were higher in MCI and sAD cases than controls, and positively correlated with histological progression or cognitive deficits (Figs. 6, 7 and 8, Additional file 1: Ext Fig. S6.1). Subtle peri-plaque Reelin aggregates were observed in ProS-CA1 in some sAD cases (Additional file 1: Ext Fig. S6.2). However, Reelin aggregates were less common and much less prominent in ProS-CA1 than we previously observed in the CA2 and CA3 regions of the hippocampus [131] (see Additional file 1: Ext Fig. S6.2) and thus did not correlate with histological progression or antemortem cognitive deficits (Fig. 6). MP-IHC revealed that within the ProS-CA1 border region Dab1, pP85α_Tyr607_, and pPSD95_Thr19_ generally accumulated together with pTau_Ser202/Thr205_ in swollen dystrophic neurites surrounding ApoE-enriched NPs and within a subset of neighboring ApoER2-expressing neurons (Figs. 7 and 8). In a recent publication [131], we described large plaque-associated Dab1 complexes in the molecular layer of the dentate gyrus and the CA2 region of the hippocampus in sAD cases wherein Dab1 was primarily localized to MAP2-labeled dystrophic dendrites. In the present study, we observed similar large Dab1 complexes in three layers [stratum oriens (SO), stratum pyramidale (SP), stratum radiatum (SR)] of the ProS-CA1 border region in sAD cases (Fig. 7). As expected, MP-IHC in the SP layer suggested that globular Dab1 accumulated primarily within MAP2-labeled swollen, dystrophic dendrites in the vicinity of ApoE-enriched NPs. Dab1-enriched dystrophic dendrites in the SP layer appear to emanate from one or more neighboring ApoER2-expressing neurons that co-accumulated multiple ApoER2-Dab1 components including pP85α_Tyr607_, pPSD95_Thr19_ and pTau_Ser202/Thr205_ (Fig. 7). Unexpectedly, in the SR layer, Dab1 accumulated primarily within NFL-positive (MAP2-negative) dystrophic axons surrounding ApoE-enriched NPs (Fig. 7). In the SO layer, Dab1 accumulated in both MAP2-labeled dendrites and NFL-labeled axons (Fig. 7), suggesting a dual dendritic and axonal origin for Dab1 in the vicinity of extracellular lipoprotein deposits.

We next sought to determine if accumulation of ApoER2-Dab1 components in the ProS-CA1 border region is evident in the early stages of sAD. Single-target IHC in a Braak stage III (MMSE 28/30) MCI case (Fig. 8) revealed that even in this early stage when NFTs are sparse in the hippocampus [3], pTau pathology was accompanied by prominent accumulations of Dab1, pDab1_Tyr220_, pP85α_Tyr607_, pLIMK1_Thr508_, and pPSD95_Thr19_. Dab1 was most prominent within globular structures in the immediate vicinity of NPs. By contrast, pP85α_Tyr607_, pLIMK1_Thr508_ and pDab1_Tyr220_ were most prominent in GVD-like vacuolar structures that were localized to the soma and neuritic projections, and as solitary vacuolar structures in the neuropil (open arrows in Fig. 8A_4,9__&19_). pLIMK1_Thr508_ also labeled discrete punctae in neuropil (solid arrows in Fig. 8A_6–10_). pPSD95_Thr19_ was prominent in GVD-like structures, small punctae surrounding affected neurons, and dystrophic neurites in the vicinity of NPs. MP-IHC suggested that Dab1, pP85α_Tyr607_, pTau_Ser202/Thr205_ and pPSD95_Thr19_ accumulated together within many of the same ApoER2-expressing abnormal neurons (Fig. 8C_1–5_) and MAP2/ApoER2-labeled dystrophic dendrites in the vicinity of affected neurons and ApoE-enriched NPs (Fig. 8C_6–10_).

### Dab1 accumulates together with ApoER2-Dab1 partners in temporal neocortex in MCI and sAD

**Fig. 9:**
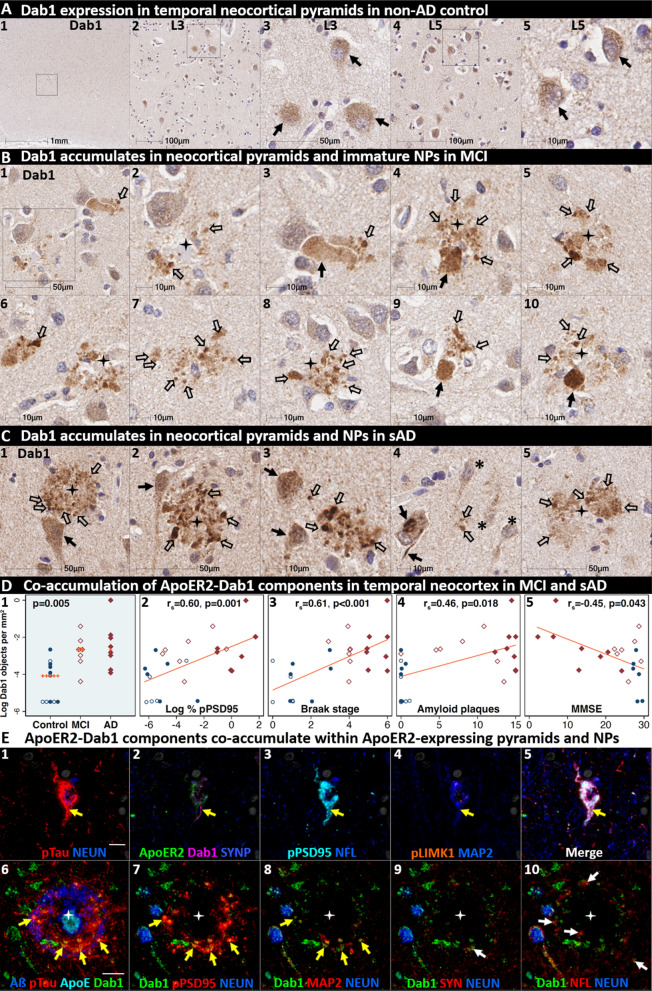
Dab1 accumulates together with ApoER2 signaling partners in temporal cortex in MCI and sAD. **A** Serial coronal sections of the temporal neocortex from representative non-AD control (Braak stage I), MCI (Braak stage III) and sAD (Braak stage VI) cases were probed with antibodies targeting ApoER2-Dab1 pathway components (see Additional file 1: Table S3). In the non-sAD control (**A**), single-target IHC revealed that Dab1 expression is confined to a subset of pyramidal neurons (solid arrows). In MCI (**B**) and sAD (**C**) cases, prominent accumulations of Dab1 were observed within abnormal neurons and within clusters of globular structures (open arrows in **B** and **C**) in the vicinity of NPs (black stars) that in some cases appeared to emanate from neighboring neurons (black arrows in **A**_**3–4**_,** A**_**9–10**_,** C**_**1–2**_). Intraneuronal Dab1 inclusions are evident in a subset of L3 and L5 pyramids (solid arrow **C4**); neighboring neurons with little or no Dab1 expression are designated with an * in **C**_**4**_. **D** Dab1 accumulation increased across the clinicopathological spectrum of sAD and correlated with pPSD95_Thr19_, Braak stage, Aβ plaque load, and cognitive deficits. In the dot plot, open and closed blue circles indicate young controls and age-matched controls; open and closed red diamonds indicate MCI cases and sAD cases, respectively. *p* value is from a Kruskal–Wallis test. Fitted line plots show Spearman’s rank correlation coefficients and *p* values. **E** MP-IHC suggested that Dab1, pTau_Ser202/Thr205_ and pPSD95_Thr19_ accumulate within the same ApoER2-expressing, NFT-bearing L5 and L3 pyramids (yellow arrows in **E**_**1–5**_) and MAP2-labeled dystrophic dendrites (yellow arrows in **E**_**6–10**_) in the immediate vicinity of Aβ plaques with ApoE enriched in the central core (white stars in **E**_**6–10**_). A small portion of Dab1 deposits appeared to accumulate within NFL-labeled axons or synaptophysin-labeled presynaptic terminals (white arrows in **E**_**9–10**_). Scale bars in MP-IHC images = 10 µm

Having shown that a subset of temporal L5 and L3 neocortical pyramids and their basal and apical dendritic projections strongly express ApoER2 (Fig. 3), we sought to determine if ApoER2-Dab1 pathway components accumulate in the temporal neocortex and whether such accumulations increase across the clinicopathological spectrum of sAD. In a recent report using IHC in middle temporal gyrus sections [131], we observed that pPSD95_Thr19_ accumulates within abnormal neurons and NPs in MCI and sAD cases and correlates with Braak stage, Aβ plaque load, and cognitive deficits. In the present study, single-marker IHC using serial sections from the same cohort revealed that Dab1 accumulates in MCI and sAD cases (Fig. 9) with strong signals localized to abnormal neurons, their basal dendritic arbors, and within the neuritic components of NPs. These Dab1-positive complexes increased across the spectrum of sAD and correlated with pPSD95_Thr19_, Braak stage, Aβ plaque load, and cognitive deficits (Fig. 9D). MP-IHC revealed that Dab1 generally accumulated together with pTau and pPSD95_Thr19_ in many of the same ApoER2-expressing, NFT-bearing pyramids (Fig. 9E_1–5_) and within swollen MAP2-positive dystrophic dendrites in the immediate vicinity of ApoE-enriched NPs (Fig. 9E_6–8_). However, as previously shown in the ErC and ProS-CA1 border region, a subset of these peri-plaque Dab1 accumulations in the temporal neocortex co-localized with NFL-labeled dystrophic axons (Fig. 9E_10_, Additional file 1: Ext Fig. S9.1).

Since Aβ plaques can precede widespread pTau pathology in neocortex, we next searched for evidence of peri-plaque Dab1 accumulation in cases that have substantial neocortical Aβ plaques but minimal pTau pathology. Single-target IHC in a non-AD control with Aβ plaques (Thal phase 3) but no overt pTau pathology (Braak stage 0) revealed multiple, globular plaque-associated Dab1 accumulations that were most prominent in L5 and L3 (Additional file 1: Ext Fig. S9.2), with little or no pTau_Ser202/Thr205_ or pPSD95_Thr19_ evident in serial sections. MP-IHC revealed that some of these globular Dab1 accumulations were clustered around an ApoE-enriched plaque core (Additional file 1: Ext Fig. S9.2). When considered together with evidence from model systems that ApoER2-Dab1 signaling regulates both conversion of AβPP to Aβ [59, 76, 78] and GSK3β-mediated Tau phosphorylation [9, 50, 72, 125, 136], our findings suggest that Dab1 could potentially serve as a convergence point linking ApoE to both Aβ and pTau pathologies in early sAD. Future studies are needed to determine if these early plaque-associated Dab1 lesions in temporal neocortex are localized to axons or dendrites.

### Co-accumulation of ApoER2-Dab1 components in pontine LC-PC complex and raphe nucleus in sAD

**Fig. 10:**
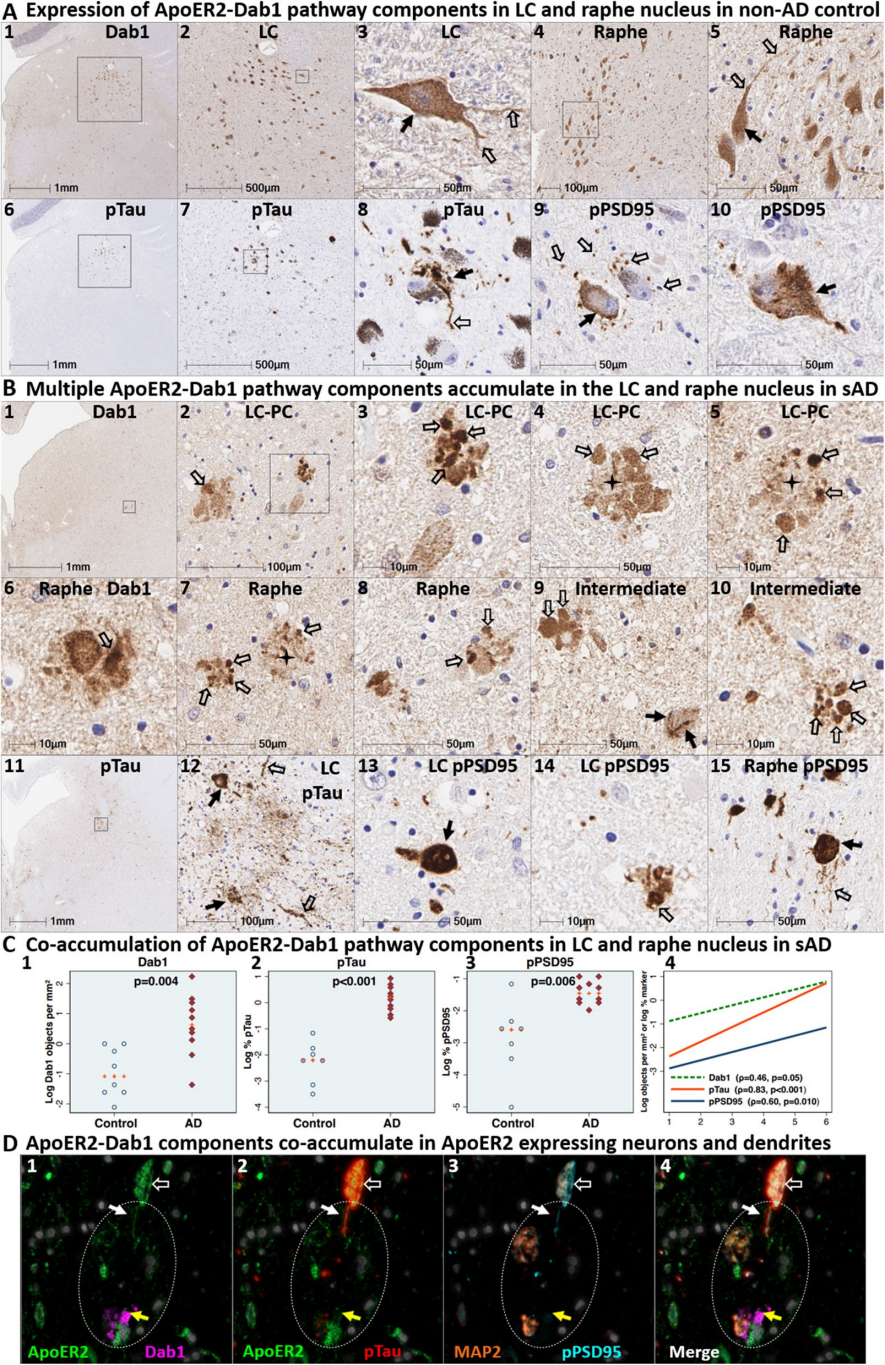
Dab1 accumulates together with ApoER2 signaling partners in LC and raphe nucleus in sAD. **A** Serial transverse sections of the upper pons from representative non-AD control (Braak stage I) and sAD (Braak stage V) cases were probed with antibodies targeting ApoER2-Dab1 pathway components (see Additional file 1: Table S3). In the non-sAD controls (**A**), single-target IHC revealed that Dab1 is expressed by a subset of LC and raphe nucleus neurons (solid arrows in **A**_**3**_**, A**_**5**_) and their neuritic projections (open arrows in **A**_**3**_**, A**_**5**_). pTau_Ser202/Thr205_ and pPSD95_Thr19_ accumulated within small subsets of LC and raphe nucleus neurons (solid arrows in **A**_**8–10**_). pTau_Ser202/Thr205_ also accumulated in neuritic projections (open arrows in **A8**) while pPSD95 accumulated as discrete punctae in adjacent neuropil (open arrows in **A8**). In sAD cases (**B**), prominent accumulations of Dab1 were observed within clusters of globular structures (open arrows in **B**_**2–10**_) and as inclusions within abnormal neurons (solid arrows in **B**_**9**_). Additional examples of intraneuronal Dab1 inclusions are provided in Additional file 1: Ext Fig. S10.1 **C** Dab1 accumulated across the clinicopathological spectrum of sAD and correlated with Braak stage. For dot plots, *p* values are from Kruskal–Wallis tests. The fitted line plot shows Spearman’s rank correlation coefficients and *p* values. **D** MP-IHC suggested that Dab1, pTau_Ser202/Thr205_, and pPSD95_Thr19_ accumulate within the same ApoER2-expressing neurons (**D**_**1–4**_) and appeared to accumulate within MAP2-positive dendritic arbors (white circles in **E**_**1–4**_). Scale bars in MP-IHC images = 10 µm

We next sought to determine if Dab1 and other ApoER2-Dab1 pathway components accumulate in the vicinity of the LC and raphe nucleus in sAD cases and neurologically normal controls using sections generated from carefully dissected pons specimens. In sAD cases, we observed that Dab1, pPSD95_Thr19_, and pTau_Ser202/Thr205_ accumulate in both the LC–PC complex and raphe nuclei, and in the vicinity of scattered clusters of multipolar neurons located between these two nuclei (Fig. 10 and Additional file 1: Ext Fig. S10.1). Accumulations of all three markers were less pronounced or absent in non-AD controls, with accumulations positively correlating with Braak stage (Fig. 10C). Dab1 accumulated both as large globular neuritic complexes (Fig. 10B_2–10_) and as intraneuronal inclusions (Fig. 10B_9_ and Additional file 1: Ext Fig. S10.1). Prominent pTau_Ser202/Thr205_ accumulations were observed in neuronal perikarya and in neighboring neuritic projections. pPSD95_Thr19_ accumulations had a similar distribution as pTau; however, unlike pTau, pPSD95_Thr19_ was most prominent within neuronal perikarya and discrete punctae surrounding affected neurons, with comparatively little staining of neuritic projections. MP-IHC suggested that Dab1 accumulated together with pPSD95_Thr19_ and pTau_Ser202/Thr205_ within many of the same ApoER2-expressing neurons and within MAP2-labeled dendritic arbors that appear to emanate from neighboring ApoER2-expressing neurons (Fig. 10D). Extracellular accumulation of the ApoER2 ligand ApoE was observed in many sAD cases (Additional file 1: Ext Fig. S10.2). Reelin-positive extracellular accumulations were observed in a few sAD cases (Additional file 1: Ext Fig. S10.2) but were not evident in most cases.

## Discussion

Fundamental questions about the origin and progression of pTau pathology in sAD have remained unanswered for decades. Our data support a model in which ApoER2-Dab1 disruption is the underlying cause of pTau-related neurodegeneration in humans. In contrast to the prevailing hypothesis for NFT progression—that Tau spreads in a prion-like manner—our findings suggest that pTau is locally produced by ApoER2-expressing neurons at multiple anatomical locations in response to ApoER2-Dab1 disruption. Since the ApoER2-Dab1 pathway regulates memory through delivery of essential lipids and stabilization of actin, microtubules, and synapses (Fig. 2), our finding that multiple pathway components co-accumulate in each affected region reframes Tau pathology as only one of many consequences of pathway disruption. As an alternative to prion-like spread, ApoER2-Dab1 disruption provides a single, shared mechanism that could: (1) account for the actin and microtubule destabilization, synaptic dysfunction, extracellular lipoprotein deposition, and cognitive deficits that characterize sAD; (2) help explain both the origin(s) and stereotypical progression of NFT pathology; (3) mechanistically link the etiology of NFTs to NPs, NTs, and GVDs; and (4) integrate these four pTau lesions with other hallmark (ApoE, Aβ, ApoJ) and emerging (Reelin, Dab1) neuropathological features to generate a unifying model for sAD in humans.

### Can ApoER2 expression and demand for activation explain the origin(s) and progression of NFT pathology?

ApoER2-Dab1 disruption promotes Tau hyperphosphorylation through compromised Reelin and ApoE signaling [9, 50, 72, 125, 136]. Since ApoER2 is not ubiquitously expressed, our finding that ApoER2 is strongly expressed in the same regions, layers, neurons and subcellular compartment (distal dendrites) [27, 29, 164] that accumulate pTau earliest in sAD is consistent with our proposed model in which multisite ApoER2 disruption drives development of NFTs. Whereas the presence of ApoER2 may be required for NFT formation, a high demand for activation of the ApoER2-Dab1 pathway (with incessant turnover of core pathway components) could also contribute to the selective vulnerability of ApoER2-expressing neurons. It is therefore notable that ErC and LC neurons are active nearly continuously from birth until death due to their pivotal roles in memory formation during waking hours and memory consolidation during sleep [47, 67, 68, 115], among other vital functions [11].

### Dab1 accumulation reveals evidence for ApoER2-Dab1 pathway disruption

Activation of the Reelin-ApoER2-Dab1 pathway shapes and strengthens synaptic connections underlying learning and memory (Fig. 2) [51, 53, 72, 98, 128, 153, 156]. Since Reelin signaling through ApoER2 induces rapid proteasomal degradation of Dab1 [18, 159], the accumulation of Dab1 protein in dystrophic neurites implies a localized, functional deficit in Reelin signaling through ApoER2 [131]. Our previous finding that Dab1 accumulates in the terminal zones of the perforant path in sAD [131] provided evidence for ApoER2-Dab1 disruption in circuitry underlying memory in humans. Here, the finding that Dab1 accumulates in each of five regions that develop NFT pathology prior to the hippocampus and dentate gyrus provides evidence that ApoER2-Dab1 disruption is widespread even in the early stages of sAD. Remarkably, Dab1 accumulation was extensive in MCI, and even preceded overt pTau accumulation in some controls, indicating that ApoER2-Dab1 disruption is likely a very early degenerative phenomenon. MP-IHC suggested that Dab1 and pTau generally accumulated within the same layers, neurons, and subcellular compartment (MAP2-labeled dystrophic dendrites) in each region. Since Dab1 is an upstream regulator of GSK3β-mediated Tau phosphorylation (Fig. 2) [9, 50, 72, 125, 136], together these observations provide a basis for our model wherein the accumulations of Dab1 and pTau (in NTs, NFTs, NPs) in each region result from ApoER2-Dab1 pathway disruption in ApoER2-expressing neurons. Intriguingly, Bracher-Smith et al. [32] recently reported a genetic association between the *DAB1* locus and AD risk that was evident only in *APOE4* homozygotes, a high-risk population that accounts ≈ 10% of sAD cases [160]. Our findings of extensive Dab1 accumulation in *APOE3* homozygote and *APOE2/APOE3* heterozygote MCI and sAD cases provide evidence that ApoER2-Dab1 disruption is a shared mechanism underlying sAD that may be exacerbated by, but is not dependent on, the *APOE4* gene variant. Moreover, our findings that Dab1 accumulates together with phosphorylated forms of its binding partner P85α [17] and three downstream ApoER2-Dab1-P85α signaling partners (LIMK1_,_ Tau, PSD95) that are known to stabilize actin, microtubules, and synapses, respectively, (Fig. 2) [35, 57, 124, 131] provide insights into molecular pathways and mechanisms linking Dab1 accumulation to cytoskeletal instability and synapse loss in sAD.

### Dendritic co-accumulation of pP85α, pLIMK1, pTau and pPSD95 as evidence for ApoER2-Dab1 disruption

Dendritic spines are actin-rich protrusions that harbor excitatory synapses in postsynaptic densities, whose plasticity plays a central role in learning and memory [49, 155]. Binding of Reelin to ApoER2 stabilizes actin and microtubule cytoskeletons and postsynaptic densities by regulating phosphorylation and activation of Dab1, PI3K, LIMK1, GSK3β, Tau and PSD95 (Fig. 2) [9, 22, 35, 50, 51, 53, 57, 72, 96, 98, 121, 124, 125, 128] (reviewed in [131]). In rodent and cellular models, compromised Reelin-ApoER2-Dab1-PI3K signaling promotes GSK3β-mediated Tau hyperphosphorylation [9, 50, 72, 125, 136]. The Dab1 phosphotyrosine-binding (PTB) domain—which recruits ApoER2-Dab1 complexes to lipid rafts by simultaneously binding the polar head group of PIP2 and cytoplasmic tail of ApoER2 [147] (Fig. 2)—is required for Reelin-Dab1 signaling [80, 146]. P85α serves as a critical node in this pathway by recruiting Dab1 and PI3K to lipid rafts [17, 74], enabling formation of postsynaptic ApoER2-Dab1-PI3K-signaling complexes. Reelin triggers interactions between P85α and Dab1 to suppress GSK3β-mediated Tau phosphorylation via activation of the PI3K pathway [17], while Tyr607-phosphorylation of P85α conversely inhibits PI3K [38]. Since activated GSK3β phosphorylates PSD95 to induce synapse disassembly [121], ApoER2-Dab1 pathway disruption provides a straightforward mechanism that could account for somatodendritic accumulations of both pTau and pPSD95_Thr19_ [131]. LIMK1 phosphorylation regulates remodeling of the actin cytoskeleton of dendritic spines [13, 107]. Heredia et al. [71] observed increased numbers of pLIMK1_Thr508_ positive neurons in AD-affected regions. Similarly, in sAD hippocampus, we previously reported that pP85α_Tyr607_ and pLIMK1_Thr508_ accumulate together within GVD-like structures in neurons that accumulate pTau_Ser202/Thr205_ and pPSD95_Thr19_, and in neighboring abnormal neurons lacking overt pTau pathology [131]. In the present study, we observed that five core intra-neuronal pathway components (Dab1, pP85α_Tyr607_, pLIMK1_Thr508_, pTau_Ser202/Thr205_, PSD95_Thr19_) accumulate together within many of the same ApoER2-expressing, NFT and/or GVD-bearing neurons and MAP2-labeled dystrophic dendrites in each region. These collective observations provide multifaceted evidence for a pathogenic nexus centered around dendritic ApoER2-Dab1 disruption in the neuron populations that are most vulnerable to early NFT pathology in humans.

### ApoER2-Dab1 disruption could explain pTau pathology without invoking prion-like spread

The prevailing model cited to account for the ordered sequence of NFT progression posits that prion-like properties enable pathogenic forms of Tau to spread from donor neurons to recipient neurons and to convert normal Tau to pathogenic Tau [26, 29, 52, 56, 103]. While rodent and cellular models have shown that trans-synaptic Tau transmission is possible [69], three pivotal observations in humans appear to contradict a simple model of connectome-based spread. First, NFT progression proceeds in a direction opposite to unidirectional connectivity in the medial temporal lobe memory system (Fig. 1A) [15, 87]. Second, pTau pathology classically originates in the LC before progressing specifically to ErC L2 [26, 31]. However, LC neurons project widely throughout the brain and do not selectively innervate ErC L2 (Fig. 1B) [1, 4, 64, 90, 117]. Third, individual projection neurons classically innervate hundreds or even thousands of neighboring target neurons [70, 88, 105, 106]. Yet in the earliest stages of sAD pTau accumulates within multiple distal dendritic tips of rare, solitary L5 and L3 neocortical pyramids (Fig. 1A) while sparing neighboring neurons [29, 92]. Accommodation of the prion-like spread hypothesis therefore requires speculation about connections that are not yet known to exist [27] or a revised model of brain connectivity [29, 30]. In this connectome-inspired revision [29, 30], all pre-synaptic terminals emanating from a single donor axon synapse on dendrites emanating from a single target neuron and refrain from contacting neighboring neurons.

Our finding that multiple ApoER2-Dab1 pathway components, including several that are upstream of Tau phosphorylation (Fig. 2), accumulate in each affected region strongly suggests that pTau is locally produced by ApoER2-expressing neurons at each site. Since prion-like properties are thought to be a unique feature of Tau [5, 118], connectome-based spread of numerous ApoER2-Dab1 components (including Dab1, pP85α_Tyr607_, pLIMK1_Thr508_, pTau_Ser202/Thr205_, PSD95_Thr19_) is an exceedingly unlikely explanation for this multisite co-accumulation. Tau inclusions are known to originate in distal dendrites [27, 29, 164]. Thus, our finding that pTau accumulates together with multiple upstream and downstream ApoER2-Dab1 components in MAP2-labeled dystrophic dendrites of many ApoER2-expressing neurons is consistent with ApoER2-Dab1 disruption but not easily reconciled with prion-like spread. The prion-like spread hypothesis, as usually presented, also does not explain the point(s) of origin of the disease process, or which intrinsic molecular features account for the vulnerability of this origin [103]. By contrast, dendritic ApoER2-Dab1 pathway disruption could explain the local production and accumulation of pTau in all affected neurons including the populations where pTau pathology is classically reported to begin (LC, ErC L2).

### ApoER2-Dab1 disruption could mechanistically and spatially link four pTau-containing lesions

**Fig. 11:**
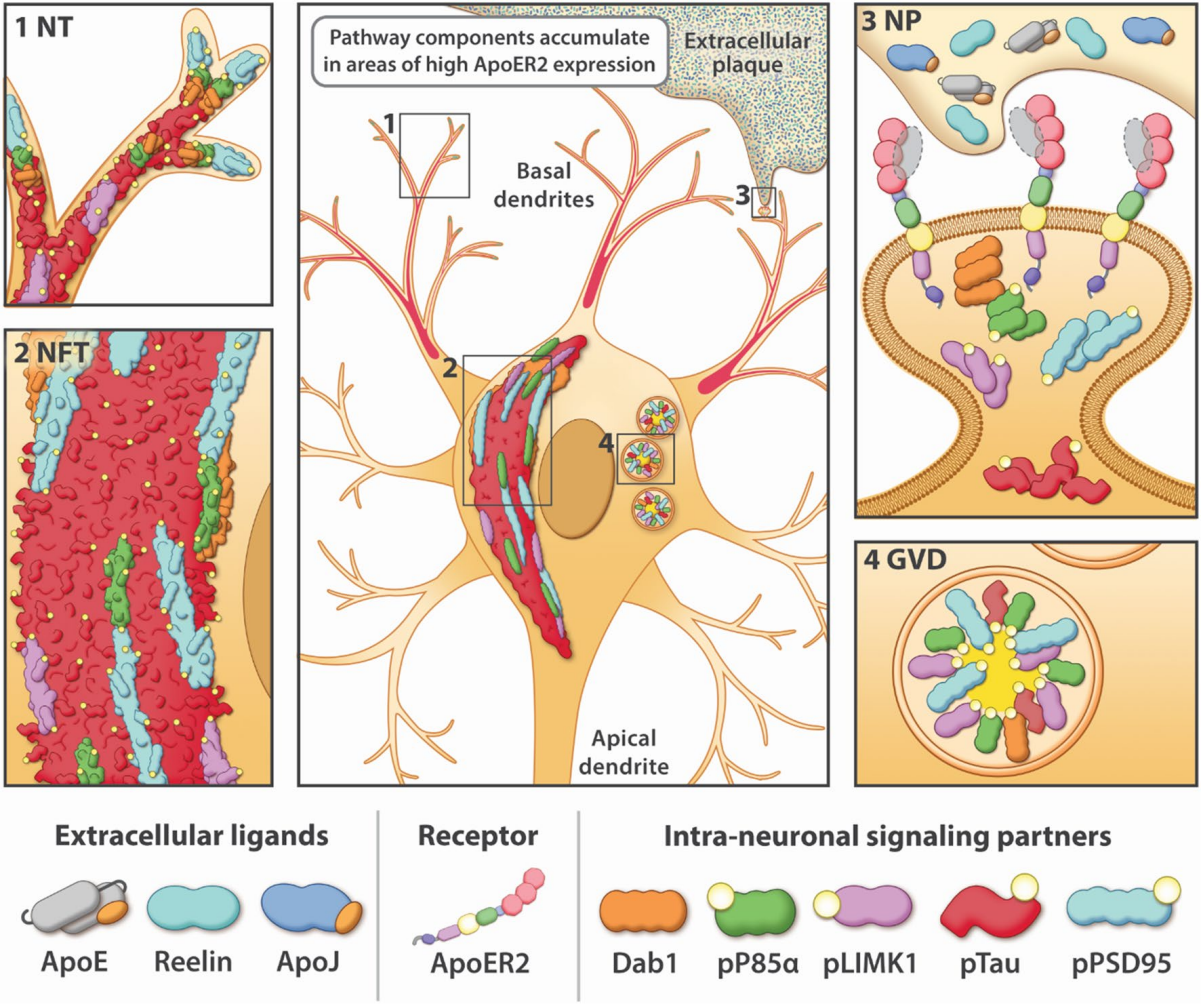
Dendritic ApoER2-Dab1 pathway disruption links four pTau-containing lesions. The ApoER2-Dab1 pathway disruption hypothesis can mechanistically and spatially link four pTau-containing lesions (NTs [1], NFTs [2], NPs [3], GVDs [4]). ApoER2-Dab1 disruption is proposed to trigger Tau hyperphosphorylation and thus the presence of ApoER2 in neuronal membranes confers vulnerability to pTau-associated neurodegeneration. In this model, a triggering molecular lesion or competitive inhibition among ligands disrupts ApoER2 binding, leading to extracellular trapping and accumulation of the ApoER2 ligands ApoE, Reelin, and ApoJ in NPs. Impaired ApoER2 signaling disrupts Dab1 degradation leading to localized neuritic Dab1 accumulation. Ensuing disruption of the Dab1-P85α/PI3K-LIMK1 arm destabilizes the actin cytoskeleton and induces pP85α_Tyr607_ and pLIMK1_Thr508_ accumulation. Impaired Reelin-ApoER2-Dab1-P85α/PI3K signaling activates GSK3β; ensuing hyperphosphorylation of Tau and PSD95 destabilizes microtubules and postsynaptic receptor complexes, and promotes accumulation of pTau_Ser202/Thr205_ and pPSD95_Thr19_ in NTs, NFTs, NPs and GVDs. Thus, the ApoER2-Dab1 pathway disruption model attributes deficits in memory and cognition in sAD to impaired neuronal lipoprotein internalization and destabilization of actin, microtubules, and synapses. Our observation that multiple ApoER2-Dab1 pathway components accumulate in the immediate vicinity of NFTs, NTs, NPs, and GVDs is consistent with this model

We previously reported that two ApoER2 ligands (ApoE and Reelin) accumulate together with extracellular Aβ and dendritic pTau in hippocampal NPs [131]. Our present finding that yet another ApoER2 ligand (ApoJ) [101] accumulates together with ApoE in the central core of many NPs, provides further evidence for ApoE receptor-ligand disruption with impaired lipoprotein internalization. ApoER2, Dab1, P85α, LIMK and PSD95 are enriched in the distal dendritic tips [10, 48, 51, 100, 107, 134] of pyramidal neurons, where pTau inclusions originate as NTs before progressing to proximal dendrites and soma of NFT-bearing neurons [27, 29]. Taken together, these findings support a model wherein dendritic ApoER2-Dab1 disruption initiates a disease-cascade resulting in both ‘extracellular trapping’ of ApoER2 ligands with lipoprotein deposition in the synaptic cleft, and accumulation of ApoER2-Dab1 signaling partners in adjacent distal dendrites (Fig. 11). GVDs are enigmatic pTau-containing lesions that are first evident in hippocampal and subicular pyramids that are considered to be pre-NFTs by some investigators [63, 79, 97, 151]. Nishikawa et al. [123] showed strong expression of PIP2 and lipid raft proteins in GVDs and NFTs, suggesting that both lesions originate in PI-enriched lipid rafts. Our finding that phosphorylated forms of ApoER2-Dab1 components known to play central roles in organizing these rafts—including P85α, Dab1, PSD95, and LIMK [48, 51, 100, 134]—accumulate alongside pTau in GVDs suggests a potential mechanistic link between ApoER2-Dab1 pathway disruption, granulovacuolar degeneration and NFT formation. Thus, dendritic ApoER2 disruption is a plausible mechanism that could help explain why NPs, NTs, NFTs and GVDs develop within spatially separated substructures (synaptic cleft, dendritic tips, soma) in the same affected neurons (Fig. 11). Consistent with this interpretation, in the ProS-CA1 border region we observed that multiple ApoER2-Dab1 pathway components accumulate in anatomically distinct areas (basal stripe, pyramidal layer, apical stripe) corresponding to spatially-separated substructures in the same neuron populations.

### Is ApoER2-Dab1 disruption also the origin of Aβ pathology in sporadic AD?

Dab1 serves as a cytoplasmic adaptor protein for both ApoER2 and AβPP [75, 166]. ApoER2-Dab1 signaling has been shown to regulate AβPP cleavage and Aβ synthesis in model systems [59, 76, 78]. AβPP is enriched in axons [110, 141], and Aβ appears to be synthesized primarily in dystrophic axon terminals [91, 137]. Although they are most abundant in dendritic spines, emerging evidence indicates that ApoER2 and Dab1 are also enriched in axonal growth cones [9], where they regulate arborization in a Reelin- and ApoE-dependent manner [21, 81, 102]. Thus, our finding that Dab1 accumulates in a subset of NFL-labeled dystrophic axons surrounding Aβ plaques suggests that axonal ApoER2-Dab1 disruption may regulate Aβ formation in humans. This finding, together with evidence that ApoER2-Dab1 signaling suppresses Tau phosphorylation in preclinical models [9, 50, 72, 125, 136] and our observation that Dab1 accumulates together with pTau_Ser202/Thr205_ in dystrophic dendrites in humans, suggests that ApoER2-Dab1 disruption could serve as a convergence point and biologically plausible shared origin linking ApoE, Reelin and Dab1 to both the Aβ plaques and pTau tangles that define sAD in humans [20].

### ApoER2-Dab1 disruption integrates formerly disjointed observations into a unifying model for sAD

Our present and previous [131] findings provide the foundation for a plausible unifying model wherein ApoE receptor-ligand disruption triggers a disease-cascade that ultimately manifests as sAD by: (1) disrupting ApoE receptor-ApoE/ApoJ dependent delivery of lipid cargo required to shape and remodel neuronal membranes; (2) disrupting ApoER2-Dab1 signaling cascades that stabilize actin and microtubule cytoskeletons and postsynaptic receptor complexes; (3) promoting Tau hyperphosphorylation and NT/NFT formation; (4) trapping lipid-laden ApoE/ApoJ particles outside neurons where they provide seeds for Aβ oligomerization [34, 111, 149]; and (5) promoting axonal Aβ synthesis and Aβ plaque formation. This model is attractive because it could help explain the origins of pTau pathology in the LC, ErC L2 and dendritic tips of solitary L5 and L3 neocortical pyramids and the sequential involvement of other neuron populations in successive Braak stages (Fig. 1) [29]. This proposed model also provides a straightforward, plausible explanation for the convergence of Aβ with pTau, ApoE, ApoJ and other ApoER2-Dab1 pathway components in NP niche, and mechanistically links the etiology of NPs to NTs, NFTs and GVDs. The ApoER2-Dab1 pathway disruption model is also consistent with established and emerging genetic risk factors for sAD [12, 99]—including variants in genes coding for ApoE [40, 99, 132, 133, 148], ApoJ [12, 55], Dab1 [32], P85α [44, 104], and components of glial pathways that govern extra-neuronal clearance of ApoE, ApoJ, Aβ, and lipids [65, 99, 165] and neuronal endolysosomal and proteasomal clearance pathways [12, 150].

### Potential implications for sAD therapeutics

The prion-like spread model provides the rationale for immunotherapeutics designed to block the spread of Tau throughout the brain [39, 89]. However, despite clear beneficial effects in mice that are genetically modified to overexpress Tau [36, 138], anti-Tau monoclonals tested thus far in MCI and early sAD patients failed to demonstrate efficacy or even worsened cognitive decline [54, 86, 119]. Since our findings suggest that pTau accumulation in humans results from multisite ApoER2-Dab1 disruption rather than pTau spread, treatments that prevent or mitigate the underlying causes of this disruption may be better positioned to delay sAD progression.

### Underlying causes of ApoER2-Dab1 pathway disruption

Plausible triggers for ApoER2-Dab1 disruption include neuritic injury-induced ApoE hypersecretion, lipoprotein peroxidation, Reelin depletion, and Aβ-induced Reelin sequestration. Astrocytes stimulate repair of injured axons by dramatically increasing local secretion of lipid-loaded ApoE particles [23, 85]. Since ApoE competes with Reelin for ApoER2 binding in model systems [46], an excess of ApoE following injury could potentially disrupt Reelin-ApoER2-Dab1 signaling. ApoE particles deliver lipids that are highly vulnerable to peroxidation to neurons via ApoE receptors including ApoER2 (reviewed in [131]). Our recent finding that ApoE and ApoER2 are vulnerable to lipid aldehyde-induced adduction and crosslinking [131] provides a mechanistic link between lipid peroxidation and ApoER2-Dab1 disruption. Since peroxidation is a common feature of many sAD risk factors [131] this mechanism could help explain why lipid peroxidation is markedly increased in the early stages of sAD [33, 60, 108, 113, 114, 116, 139, 143, 144, 167]. Since Reelin-ApoER2-Dab1 signaling suppresses Tau phosphorylation [9, 50, 72, 125, 136], and Reelin-secreting neurons are reported to degenerate in sAD [6, 37, 42, 135], Reelin depletion could potentially contribute to the intra-neuronal accumulations of pTau, Dab1, pP85α_Tyr607_, pLIMK1_Thr508_, and pPSD95_Thr19_ observed in the present study. However, unlike ApoER2-Dab1 pathway disruption, Reelin depletion alone cannot readily account for our findings that ApoER2 ligands such as ApoE and ApoJ [and in some cases Reelin itself [131] (see Additional file 1: Ext Fig. S6.2)] accumulate in the immediate vicinity of many NPs. Finally, emerging evidence that Aβ can sequester Reelin [41, 43, 94] suggests that Aβ overproduction could potentially contribute to ApoER2-Dab1 disruption. Aβ-induced Reelin sequestration may be particularly important in familial AD cases with a genetic source of Aβ overproduction [154] (and transgenic mouse models harboring these same familial AD mutations) [161]. Future studies are needed to better understand the roles of ApoE secretion, lipoprotein peroxidation, Reelin depletion and Aβ-induced Reelin sequestration in familial AD and sAD.

### Limitations and future directions

Delayed autopsy and prolonged fixation are reported to have limited impact on highly-aggregated proteins such as Aβ and pTau [129] but can obscure signals for less aggregated proteins [7, 66, 163]. Thus, use of rapidly-autopsied specimens that underwent uniform, time-limited (48 h) fixation, is an important strength of the present study. Postmortem specimens spanning the clinicopathological spectrum of sAD were used to approximate the spatiotemporal sequence of NFT progression. This cross-sectional study design cannot establish a sequence of disease progression [142]. Experimental data are therefore needed to establish a role for ApoER2-Dab1 pathway disruption in sAD. The moderate sample sizes are another important limitation. Although ApoER2-Dab1 pathologies were observed in all major *APOE* variants and both sexes, larger studies are needed to determine if results are influenced by genetics, sex, and other variables. The comparable IHC results obtained using specimens from three brain banks that employed different procedures for autopsies, sample processing and pathological assessment add confidence to study findings. However, since all three cohorts were primarily of Caucasian descent, future studies including more races and ethnicities are needed to determine generalizability. MP-IHC was performed on a subset of representative cases using thin sections with 20 × magnification. Future studies with more cases, thicker sections, and higher-resolution imaging are needed to assign ApoER2-Dab1 components to specific neurons and their dendritic arbors and to localize components to specific organelles such as LAMP1-labeled lysosomes. The human brain contains multiple *LRP8* splice isoforms [62, 126] which encode variants in domains that regulate extracellular ligand binding, formation of ApoER2-PSD95 receptor complexes in excitatory synapses, and synaptic plasticity in preclinical models [10, 73, 77, 126, 130, 162]. Since the present study used two mRNA probes designed to detect sequences present in the most abundant *LRP8* isoforms (Additional file 1: Table S3), future studies are needed to determine which specific isoforms are most closely associated with pTau accumulation and degeneration. Since Tau pathology is present predominantly but not exclusively in excitatory neurons [58, 127], future studies characterizing expression of *LRP8* isoforms in these and other neuron subpopulations may provide additional insights into ApoER2-related Tau pathology in humans. Although they lack the restricted distribution of ApoER2, other brain ApoE receptors with common ligands and adaptor proteins may contribute to observed disease manifestations via partially overlapping mechanisms. While our finding that pTau accumulated together with upstream markers of pTau production (i.e., Dab1, pP85α_Tyr607_) strongly suggests that pTau is locally produced by neurons within each affected region, it does not rule out the possibility that prion-like propagation of Tau, including pTau or Tau that is not detected by traditional methods [61, 82], could contribute to pTau-related neurodegeneration in sAD or other tauopathies.

## Summary and conclusion

We found that five neuron populations that accumulate pTau in the earliest stages of sAD strongly express ApoER2 and that pTau is only one of many ApoER2-Dab1 components that accumulate within NPs and NT-, NFT-, and GVD-bearing ApoER2-expressing neurons. Collective findings support ApoER2-Dab1 pathway disruption as a plausible, alternative explanation for why specific neurons degenerate, and integrate pTau pathology into a unifying model with other hallmark (ApoE, Aβ, ApoJ) and emerging (Reelin, Dab1) pathological features of sAD in humans.

### Supplementary Information

**Additional file 1.** Contains supplementary tables, extended figures, and supplementary text on materials and methods.

## Data Availability

The datasets and code are available from the corresponding author upon reasonable request.

## Abbreviations

AD: Alzheimer’s disease
ApoE: Apolipoprotein E
ApoER2: ApoE receptor 2
ApoJ: Apolipoprotein J
CA: Cornu ammonis
CR: Cajal–Retzius
Dab1: Disabled homolog-1
ErC: Entorhinal cortex
GVD: Granulovacuolar degeneration body
HIER: Heat induced epitope retrieval
L2: Layer II
LC: Locus coeruleus
LIMK1: LIM domain kinase-1
*LRP8*: Low-density lipoprotein receptor-related protein 8 (gene encoding ApoER2)
LRP8_M_: The midchain, beta propeller region of LRP8
LRP8_L_: The ligand binding region of LRP8
NEUN: Neuronal nuclear/soma antigen
NFL: Neurofilament light chain
NFT: Neurofibrillary tangle
NP: Neuritic plaque
NT: Neuropil thread
P85α: PI3K regulatory subunit P85alpha
PC: Peri-coeruleus
PI: Phosphatidylinositol
PIP2: Phosphatidylinositol-(4,5)-bisphosphate
PI3K: Phosphatidylinositol-3-kinase
PMI: Postmortem interval
ProS: Prosubiculum
PSD95: Postsynaptic density protein 95
pTau: PTau_Ser202/Thr205_
PTB: Phosphotyrosine binding domain
*RELN*: Gene encoding Reelin protein
sAD: Sporadic AD
SO: Stratum oriens
SP: Stratum pyramidale
SR: Stratum radiatum
SYNP: Synaptophysin
