# Supplementary material for: ApoER2-Dab1 disruption as the origin of pTau-associated neurodegeneration in sporadic Alzheimer’s disease
Source: Acta Neuropathol Commun. 2023 Dec 13;11:197. doi: 10.1186/s40478-023-01693-9 (PMC10720169; doi:10.1186/s40478-023-01693-9)
Supplement: Supplementary file 1 — Additional file 1. Contains supplementary tables, extended figures, and supplementary text on materials and methods. [file 40478_2023_1693_MOESM1_ESM.docx]

**Supplement**

| **Section** | **Page** |
| --- | --- |
| I. Supplementary Tables | 2 |
| II. Extended Figures | 13 |
| III. Supplementary Materials and Methods | 24 |

**I. Supplementary Tables**

| **Table** | **Title** |
| --- | --- |
| S1 (a) | Thirty-four cases spanning the clinicopathological spectrum of Alzheimer's disease progression (Banner). |
| S1 (b) | Eighteen cases spanning the clinicopathological spectrum of Alzheimer's disease progression (New Zealand). |
| S1 (c) | Twelve cases spanning the clinicopathological spectrum of Alzheimer's disease progression (UK-ADRC). |
| S2 (a) | Summary characteristics (Banner, n=34). |
| S2 (b) | Summary characteristics (New Zealand, n=18). |
| S2 (c) | Summary characteristics (UK-ADRC, n=12). |
| S3 | Key resources. |
| S4 | False discovery rate adjusted p-values for manuscript figures. |

**Table S1 (a). Thirty-four cases spanning the clinicopathological spectrum of Alzheimer's disease progression (Banner).**

| **ID** | **Sex** | **Age** | **PMI** | **Braak** | **Thal** | **Amyloid** | **Neuritic** | **MMSE** | **ApoE** |
| --- | --- | --- | --- | --- | --- | --- | --- | --- | --- |
|  |  | **(years) ᵃ** | **(hours)** | **stage** | **phase** | **plaques** | **plaques** | **(0-30)** | **status** |
|  |  |  |  | **(0-VI)** | **(0-5)** | **(0-15) ᵇ** |  |  |  |
| **Alzheimer's Disease** | | | | | | | | | |
| 1 | Male | 70-74 | 4.8 | VI | 5 | 14 | Frequent | 7 | 3/4 |
| 2 | Female | 70-74 | 4.9 | VI | 4 | 15 | Frequent | 17 | 3/4 |
| 3 | Female | 75-79 | 3.1 | V |  | 15 | Frequent | 6 | 3/3 |
| 4 | Male | 75-79 | 3.6 | VI | 5 | 15 | Frequent | 10 | 3/4 |
| 5 | Male | 80-84 | 4.0 | V | 5 | 14 | Frequent | 14 | 3/4 |
| 6 | Female | 80-84 | 4.0 | VI | 5 | 15 | Frequent | 19 | 3/3 |
| 7 | Female | 80-84 | 3.3 | VI | 5 | 15 | Frequent | 24 | 3/3 |
| 8 | Male | 85-89 | 2.2 | V |  | 12 | Frequent | 21 | 2/3 |
| 9 | Female | 85-89 | 3.4 | VI | 5 | 14 | Frequent | 2 | 3/3 |
| 10 | Female | ≥90 | 2.2 | V | 3 | 15 | Frequent | 13 | 3/3 |
| **Mild Cognitive Impairment** | | | | | | | | | |
| 11 | Female | 75-79 | 3.2 | IV | 2 | 4 | None | 24 | 2/3 |
| 12 | Female | 80-84 | 3.0 | IV |  | 12 | Frequent | 29 | 2/3 |
| 13 | Female | 80-84 | 3.2 | IV | 4 | 11 | Frequent | 29 | 2/3 |
| 14 | Male | 85-89 | 2.2 | III | 5 | 8 | Moderate | 28 | 3/3 |
| 15 | Male | 85-89 | 4.2 | IV | 0 | 0 | None | 23 | 3/3 |
| 16 | Female | 85-89 | 3.6 | IV | 3 | 10 | Frequent | 28 | 2/3 |
| 17 | Female | ≥90 | 3.2 | IV | 2 | 5 | Frequent | 22 | 3/3 |
| 18 | Female | ≥90 | 3.2 | IV |  | 14 | Frequent | 26 | 2/3 |
| **Age-matched Controls** | | | | | | | | | |
| 19 | Male | 70-74 | 4.6 | I | 0 | 0 | None | 29 | 3/3 |
| 20 | Male | 70-74 | 3.5 | III | 0 | 0 | None | 27 | 3/3 |
| 21 | Male | 75-79 | 2.3 | I | 2 | 6 | Sparse | 29 | 3/4 |
| 22 | Female | 75-79 | 2.5 | II | 0 | 0 | None | 28 | 3/3 |
| 23 | Female | 80-84 | 2.1 | I | 1 | 1 | Sparse | 29 | 2/3 |
| 24 | Male | 80-84 | 2.0 | I | 2 | 0 | None |  | 3/3 |
| 25 | Male | 85-89 | 3.0 | I | 2 | 4 | Sparse |  | 3/3 |
| 26 | Female | 85-89 | 3.1 | III | 0 | 0 | None | 28 | 3/4 |
| 27 | Male | ≥90 | 3.4 | I | 1 | 0 | Sparse | 27 | 3/3 |
| 28 | Male | ≥90 | 3.0 | I | 0 | 0 | None | 30 | 3/3 |
| 29 | Female | ≥90 | 3.0 | III | 3 | 6 | Sparse | 27 | 3/3 |
| **Middle-age Controls** | | | | | | | | | |
| 30 | Male | 35-39 | 3.0 | 0 | 0 | 0 | None |  | 3/3 |
| 31 | Male | 45-49 | 4.5 | 0 | 0 | 0 | None |  | 3/3 |
| 32 | Female | 50-54 | 4.7 | I | 0 | 0 | None |  | 3/3 |
| 33 | Female | 55-59 | 3.1 | I | 1 | 1 | None |  | 3/3 |
| 34 | Male | 60-64 | 2.3 | I | 1 | 0 | Sparse |  | 3/3 |
| ᵃ Ages of individual cases are presented in 5-year intervals to ensure patient confidentiality. In addition, BBDP classified all cases that were at least 90 years of age as "≥90". | | | | | | | | | |
| ᵇ Amyloid plaque density or neurofibrillary tangle density score in the following regions: frontal, temporal, parietal, hippocampus, and entorhinal cortex. | | | | | | | | | |

**Table S1 (b). Eighteen cases spanning the clinicopathological spectrum of Alzheimer's disease progression (New Zealand).**

| **ID** | **Sex** | **Age** | **PMI** | **Braak** | **Thal** | **Neuritic** | **ApoE** |
| --- | --- | --- | --- | --- | --- | --- | --- |
|  |  | **(years) ᵃ** | **(hours)** | **stage** | **phase** | **plaques** | **status** |
|  |  |  |  | **(0-VI)** | **(0-5)** |  |  |
| **Alzheimer's Disease** | | | | | | | |
| 1 | Female | 90-94 | 11.5 | VI | 5 | Moderate | 3/4 |
| 2 | Female | 60-64 | 16.0 | VI | 5 | Moderate | 4/4 |
| 3 | Female | 80-84 |  | V | 4 | Moderate | 3/4 |
| 4 | Male | 65-69 | 12.0 | V | 5 | Moderate | 3/3 |
| 5 | Female | 70-74 | 8.5 | V | 5 | Moderate | 3/3 |
| 6 | Male | 70-74 | 5.0 | V |  | Frequent | 3/3 |
| 7 | Male | 80-84 | 15.0 | V |  | Moderate | 3/4 |
| 8 | Male | 85-89 |  | IV | 3 | Sparse | 3/4 |
| 9 | Male | 85-89 | 24.0 | IV | 4 | Moderate | 3/4 |
| 10 | Male | 75-79 | 11.5 | IV | 5 | Frequent | 3/3 |
| **Age-matched Controls** | | | | | | | |
| 11 | Female | 75-79 | 20.0 | III |  |  | 3/3 |
| 12 | Male | 75-79 | 13.0 | II |  |  | 3/3 |
| 13 | Male | 70-74 | 5.5 | II |  |  | 3/3 |
| 14 | Male | 70-74 | 13.0 | II |  |  | 3/3 |
| 15 | Male | 70-74 | 23.0 | I |  |  | 3/3 |
| 16 | Male | 75-79 | 23.0 | I |  |  | 3/3 |
| 17 | Male | 60-64 | 9.0 | I |  |  | 3/3 |
| 18 | Male | 55-59 | 24.5 | I |  |  | 3/3 |
| ᵃ Ages of individual cases are presented in 5-year intervals to ensure patient confidentiality. | | | | | | | |

**Table S1 (c). Twelve cases spanning the clinicopathological spectrum of Alzheimer's disease progression (UK-ADRC).**

| **ID** | **Sex** | **Age** | **PMI** | **Braak** | **Thal** | **Neuritic** | **MMSE** | **ApoE** |
| --- | --- | --- | --- | --- | --- | --- | --- | --- |
|  |  | **(years) ᵃ** | **(hours)** | **stage** | **phase** | **plaques** | **(0-30)** | **status** |
|  |  |  |  | **(0-VI)** | **(0-5) ᵇ** |  |  |  |
| **Alzheimer's Disease** | | | | | | | | |
| 1 | Female | 90-94 | 2.8 | VI |  | Frequent | 0 | 3/3 |
| 2 | Male | 80-84 | 3.3 | VI | 5 | Frequent | 2 | 3/4 |
| 3 | Male | 75-79 | 3.3 | VI |  | Frequent | 5 | 4/4 |
| 4 | Female | 85-89 | 2.2 | VI | 5 | Frequent | 10 | 2/3 |
| **Mild Cognitive Impairment** | | | | | | | | |
| 5 | Male | 80-84 | 3.5 | IV |  | Moderate | 24 | 3/4 |
| 6 | Male | 85-89 | 2.8 | IV |  | None | 27 | 3/3 |
| 7 | Female | 90-94 | 2.3 | III | 3 | None | 28 | 3/4 |
| 8 | Female | 80-84 | 2.5 | II | 0 | None | 26 | 3/3 |
| **Age-matched Controls** | | | | | | | | |
| 9 | Male | 85-89 | 4.0 | II | 2 | None | 28 | 3/3 |
| 10 | Female | 90-94 | 1.3 | I |  | None | 24 | 3/3 |
| 11 | Female | 80-84 | 2.8 | I | 1 | None | 30 | 3/4 |
| 12 | Male | 70-74 | 2.6 | 0 | 3 | None | 28 | 3/3 |
| ᵃ Ages of individual cases are presented in 5-year intervals to ensure patient confidentiality. | | | | | | | | |
| ᵇ Thal phase not available for autopsies prior to 2012. | | | | | | | | |

**Table S2 (a). Summary characteristics.**

**Banner (n=34) ᵃ**

|  | **AD (n=10)** | **MCI (n=8)** | **Control (n=11)** | **Young Control (n=5)** |
| --- | --- | --- | --- | --- |
| **Demographic and clinical characteristics** |  |  |  |  |
| Age, y, median (range) ᵇ | 82 (73-90) | 85 (76-90) | 84 (71-90) | 52 (38-61) |
| Education, y, median (range) | 14 (12-16) | 13 (12-16) | 15 (10-18) | 16 (14-19) |
| Female | 6 | 6 | 4 | 2 |
| Post-mortem interval, hours, median (range) | 3.5 (2.2-4.9) | 3.2 (2.2-4.2) | 3.0 (2.0-4.6) | 3.1 (2.3-4.7) |
| **Neuropathology and genetics** |  |  |  |  |
| Thal phase (0-5), mean (range) | 5 (3-5) | 2 (0-5) | 1 (0-3) | 0 (0-1) |
| Braak stage (0-6), median (range) | 6 (5-6) | 4 (3-4) | 1 (1-3) | 1 (0-1) |
| Neurofibrillary tangle (0-15), median (range) ᶜ | 15 (10-15) | 7 (5-8) | 2 (0-4) | 0 (0-0) |
| Entorhinal cortex (0-3), median (range) | 3 (3-3) | 3 (2-3) | 1 (0-2) | 0 (0-0) |
| Hippocampus (0-3), median (range) | 3 (2-3) | 3 (2-3) | 0 (0-2) | 0 (0-0) |
| Temporal cortex (0-3), median (range) | 3 (2-3) | 1 (0-2) | 0 (0-1) | 0 (0-0) |
| Neuritic plaque density (0-3), median (range) | 3 (3-3) | 3 (0-3) | 0 (0-1) | 0 (0-1) |
| Amyloid plaques (0-15), median (range) ᶜ | 15 (12-15) | 9 (0-14) | 0 (0-6) | 0 (0-1) |
| Entorhinal cortex (0-3), median (range) | 3 (2-3) | 2 (0-3) | 0 (0-2) | 0 (0-0) |
| Hippocampus (0-3), median (range) | 3 (1-3) | 0 (0-2) | 0 (0-1) | 0 (0-0) |
| Temporal cortex (0-3), median (range) | 3 (3-3) | 2 (0-3) | 0 (0-2) | 0 (0-1) |
| **Cognitive endpoints** |  |  |  |  |
| Cognitive dysfunction, y, median (range) | 8 (1-15) |  |  |  |
| MMSE (0-30), median (range) | 14 (2-24) | 27 (22-29) | 28 (27-30) |  |
| Clinical Dementia Rating sum of boxes (0-18), median (range) | 13 (0-18) | 0 (0-2) | 0 (0-0) |  |
| Clinical Dementia Rating global score (0-3), median (range) | 2 (0-3) | 0 (0-0) | 0 (0-0) |  |
| FAST Score (1-7), median (range) | 4 (1-6) | 2 (1-3) | 2 (1-2) |  |
| Figure Recall Score (0-3), median (range) | 1 (0-2) | 2 (1-2) | 3 (3-3) |  |
| AVLT Total Learning (0-75), median (range) ᶜ | 21 (12-46) | 48 (21-48) | 45 (25-52) |  |
| AVLT STM A6 (0-15), median (range) | 4 (0-8) | 9 (4-10) | 11 (8-13) |  |
| WMSR Digit Span Forward Score (0-12), median (range) | 8 (6-9) | 8 (6-12) | 8 (7-11) |  |
| ApoE, n |  |  |  |  |
| 3/3 | 5 | 3 | 8 | 5 |
| 2/3 | 1 | 5 | 1 | 0 |
| 3/4 | 4 | 0 | 2 | 0 |
| NIA-Reagan, Likelihood of Alzheimer's disease, n ᵈ |  |  |  |  |
| Not AD | 0 | 0 | 2 | 0 |
| Intermediate | 0 | 1 | 0 | 0 |
| High | 10 | 0 | 0 | 0 |
| Criteria Not Met | 0 | 7 | 9 | 5 |
| Dementia not otherwise specified, n | 0 | 0 | 0 | 0 |
| Hippocampal sclerosis, n | 0 | 0 | 0 | 0 |
| Vascular dementia, n ᵉ | 0 | 0 | 0 | 0 |
| ᵃ Some markers have fewer than 30 cases (no less than 21). ᵇ BBDP classified all cases >90 years of age as "90 years of age" to ensure confidentiality. ᶜ Total scores for plaques and tangles include the entorhinal, hippocampus, temporal, parietal, and frontal areas. Each was scored according to the CERAD templates [2] using Campbell-Switzer silver stain, Gallyas silver stain and Thioflavin S stains. ᵈ Modified NIA-Reagan diagnosis of Alzheimer's disease based on consensus recommendations for postmortem diagnosis of Alzheimer's disease [1] including neurofibrillary tangles (Braak) and neuritic plaques (CERAD). ᵉ Defined by NINDS-AIREN criteria.[3] | | | | |

**REFERENCES**

1 (1997) Consensus recommendations for the postmortem diagnosis of Alzheimer's disease. The National Institute on Aging, and Reagan Institute Working Group on Diagnostic Criteria for the Neuropathological Assessment of Alzheimer's Disease. Neurobiol Aging 18: S1-2

2 Mirra SS, Heyman A, McKeel D, Sumi SM, Crain BJ, Brownlee LM, Vogel FS, Hughes JP, van Belle G, Berg L (1991) The Consortium to Establish a Registry for Alzheimer's Disease (CERAD). Part II. Standardization of the neuropathologic assessment of Alzheimer's disease. Neurology 41: 479-486 Doi 10.1212/wnl.41.4.479

3 Roman GC, Tatemichi TK, Erkinjuntti T, Cummings JL, Masdeu JC, Garcia JH, Amaducci L, Orgogozo JM, Brun A, Hofman Aet al (1993) Vascular dementia: diagnostic criteria for research studies. Report of the NINDS-AIREN International Workshop. Neurology 43: 250-260 Doi 10.1212/wnl.43.2.250

**Table S2 (b). Summary characteristics.**

**New Zealand (n=18)**

|  | **AD (n=10)** | **Control (n=8)** |
| --- | --- | --- |
| Age, y, median (range) | 79 (60-94) | 73 (59-78) |
| Female | 4 | 1 |
| Post-mortem interval, hours, median (range) | 12 (5-24) | 16 (6-24) |
| Thal phase (0-5), mean (range) | 5 (3-5) |  |
| Braak stage (0-6), median (range) | 5 (4-6) | 2 (1-3) |
| Neuritic plaque density (0-3), median (range) | 2 (1-3) |  |
| Cognitive dysfunction, y, median (range) | 13 (4-25) |  |
| ApoE, n |  |  |
| 3/3 | 4 | 8 |
| 3/4 | 5 | 0 |
| 4/4 | 1 | 0 |

**Table S2 (c). Summary characteristics.**

**UK-ADRC (n=12)**

|  | **AD (n=4)** | **MCI (n=4)** | **Control (n=4)** |
| --- | --- | --- | --- |
| Age, y, median (range) | 84 (75-91) | 86 (84-92) | 84 (71-92) |
| Education, y, median (range) | 14 (12-20) | 17 (13-18) | 17 (15-18) |
| Female, n | 2 | 2 | 2 |
| Post-mortem interval, hours, median (range) | 3.0 (2.2-3.3) | 2.6 (2.3-3.5) | 2.7 (1.3-4.0) |
| Brain weight, grams, mean (SD) | 1085 (880-1130) | 1245 (1020-1530) | 1268 (1050-1495) |
| Braak stage (0-6), median (range) | 6 (6-6) | 4 (2-4) | 1 (0-2) |
| MMSE (0-30), median (range) | 4 (0-10) | 26 (24-28) | 28 (24-30) |
| Clinical Dementia Rating global score (0-3), median (range) | 3 (2-3) | 0 (0-0) | 0 (0-2) |
| ApoE, n |  |  |  |
| 3/3 | 1 | 2 | 3 |
| 2/3 | 1 | 0 | 0 |
| 3/4 | 1 | 2 | 1 |
| 4/4 | 1 | 0 | 0 |

**Table S3. Key Resources**

| ***Reagent type or resource*** | ***Target*** | ***Designation*** | ***Type*** | ***Source*** | ***Catalog #*** | ***Previous uses and validation*** | ***IHC conditions*** |
| --- | --- | --- | --- | --- | --- | --- | --- |
| **RNA probes** | |  |  |  |  |  |  |
| ISH probe | LRP8 mRNA probe 1  (M = Midchain β-propeller region) | *LRP8***M** | ISH probe | ACD, Biotechne | 807461 | ISH in human brain FFPE specimens,  RNA-protein co-detection |  |
| ISH probe | LRP8 mRNA probe 2  (L = Ligand binding region) | *LRP8***L** | ISH probe | ACD, Biotechne | 1160541 | Custom-designed for this study,  first use for RNA-protein co-detection |  |
| ISH probe | Reelin mRNA probe | *RELN* | ISH probe | ACD, Biotechne | 413051 | ISH in human brain organoids |  |
| **Primary antibodies for IHC** | |  |  |  |  |  |  |
| antibody | ApoE receptor 2 | ApoER2 | Rabbit IgG | Millipore-Sigma | SAB2103110 | IHC human brain FFPE, positive and negative control IHC, WB in OE vs Control lysates, multi-epitope labeling | pH6 at 70C for 40 min; 1:80-1:150 |
| antibody | Disabled homolog 1 | Dab1 | Rabbit IgG | Invitrogen | PA5-86617 | IHC human brain FFPE, positive and negative control IHC, WB in OE vs Control lysates, multi-epitope labeling | pH6 at 70C for 40 min; 1:50 |
| antibody | Tyr607-phosphorylated P85α | pP85α_Tyr607_ | Rabbit IgG | Invitrogen | PA5-104853 | IHC human brain FFPE, positive and negative control IHC, WB, WB with phospho-blocking peptide | pH6 at 70C for 40 min; 1:100-1:150 |
| antibody | Thr508-phosphorylated LIM kinase-1 | pLIMK1_Thr508_ | Rabbit IgG | Invitrogen | PA5-104925 | IHC human brain FFPE, positive and negative control IHC, WB, WB with phospho-blocking peptide | pH6 at 70C for 20 min; 1:100 |
| antibody | Ser202/Thr205-phosphorylated Tau | pTau | Mouse IgG1 [AT8] | Invitrogen | MN1020 | IHC human brain FFPE, WB | pH6 at 70C for 40 min; 1:100 |
| antibody | Thr19-phosphorylated PSD95 | pPSD95_Thr19_ | Rabbit IgG | Millipore-Sigma | ABN998 | IHC human brain FFPE, positive and negative control IHC, WB in OE vs Control lysates, multi-epitope labeling | pH6 at 70C for 40 min; 1:50 |
| antibody | Apolipoprotein E | ApoE | Mouse IgG1 [WUE4] | Novus Biologicals | NB110-60531 | IHC human brain FFPE, positive and negative control IHC, WB, multi-epitope labeling | pH6 at 70C for 40 min; 1:60-1:100 |
| antibody | Apolipoprotein E | ApoE | Chicken IgG | Biosynth | NEP4809 | IHC human brain FFPE, positive and negative control IHC, WB, multi-epitope labeling | 88% formic acid for 10 min, 1:50 |
| antibody | Apolipoprotein J | ApoJ | Rabbit IgG | Invitrogen | PA5-24426 | IHC human brain FFPE, positive and negative control IHC, WB | pH6 at 70C for 40 min; 1:100-1:150 |
| antibody | Reelin | Reelin | Mouse IgG2a [E-5] | Santa Cruz | SC-25346 | IHC human brain FFPE, positive and negative control IHC, WB | pH6 at 70C for 25 min; 1:50 |
| antibody | Amyloid Beta Protein | Aβ | Mouse IgG2b [MOAB-2] | Novus Biologicals | NBP2-13075 | IHC human brain FFPE, positive and negative control IHC, WB, multi-epitope labeling | 88% formic acid for 10 min, 1:100 |
| antibody | Tyr220-phosphorylated Dab1 | pDab1_Tyr220_ | Rabbit IgG | Invitrogen | PA5-104586 | IHC human FFPE brain, positive and negative control IHC, WB, WB with phospho-blocking peptide | pH6 at 70C for 30 min; 1:50 |
| antibody | LDL receptor related protein 1 | LRP1 | Mouse IgG1 [A2MR-beta1] | Invitrogen | 37-7600 | IHC human FFPE specimens, positive and negative control IHC, WB | pH9 at 70C for 40 min; 1:100 |
| antibody | Very low-density lipoprotein receptor | VLDLR | Mouse IgG1 [VL1A9] | Diagnocine | BML031 | IHC human brain FFPE, positive and negative control IHC, WB | 88% formic acid for 10 min,1:150-1:200 |
| **Cytoarchitectural antibodies for multiplex-IHC** | |  |  |  |  |  |  |
| antibody | Neuronal marker NeuN | NEUN | Guinea Pig IgG | Millipore Sigma | ABN90P | IHC human brain FFPE, positive and negative control IHC, WB | pH9 at 70C for 40 min; 1:100 |
| antibody | Microtubule associated protein 2 | MAP2 | Mouse IgG3 [885232] | R&D Systems | MAB8304 | IHC human brain FFPE, positive and negative control IHC | pH9 at 70C for 40 min; 1:100 |
| antibody | Neurofilament light chain | NFL | Mouse IgG1 [NFL2] | Biolegend | 846002 | IHC human brain, positive and negative control IHC, WB, multi-epitope labeling | pH9 at 70C for 40 min; 1:100 |
| antibody | Neurofilament light chain | NFL | Mouse IgG1 [NFL3] | Biolegend | 845902 | IHC human brain, positive and negative control IHC, WB, multi-epitope labeling | pH9 at 70C for 40 min; 1:100 |
| antibody | Synaptophysin | SYNP | Mouse IgM [SP15] | Millipore Sigma | MAB329 | IHC human brain FFPE, positive and negative control IHC, WB | pH9 at 70C for 40 min; 1:100 |
| **Secondary Antibodies for IHC** | |  |  |  |  |  |  |
| antibody | Goat anti-Rat IgG |  |  | Jackson | 112-035-167 |  | 1.6 ug/mL |
| antibody | Goat anti-Mouse IgG1 |  |  | Jackson | 115-035-205 |  | 1.6 ug/mL |
| antibody | Goat anti-Mouse IgG2a |  |  | Jackson | 115-035-206 |  | 1.6 ug/mL |
| antibody | Goat anti-Mouse IgG2b |  |  | Jackson | 115-035-207 |  | 1.6 ug/mL |
| antibody | Goat anti-Mouse IgG3 |  |  | Jackson | 115-035-209 |  | 1.6 ug/mL |
| antibody | Goat anti-Mouse IgM |  |  | Jackson | 115-035-075 |  | 1.6 ug/mL |
| antibody | Donkey anti-Rabbit IgG |  |  | Jackson | 711-035-152 |  | 1.6 ug/mL |
| antibody | Donkey anti-Chicken IgY |  |  | Jackson | 703-035-155 |  | 1.6 ug/mL |
| **Additional primary antibodies and transient overexpression and empty vector control lysates used for multi-epitope Immunodetection of target protein experiments** | | | | | | | ***Conditions*** |
| antibody | ApoE receptor 2 | ApoER2 | Rabbit IgG | Biosynth | NEP4929 | WB in OE vs Control lysates, multi-epitope labeling | Fig S1 legend |
| lysate | LRP8 (gene encoding ApoER2) lysate | ApoER2 OE lysate | HEK293T transient overexpression lysate | Novus Biologicals | NBP2-06973 | WB in OE vs Control lysates, multi-epitope labeling | Fig S1 legend |
| antibody | Disabled homolog 1 | Dab1 | Rabbit IgG | Invitrogen | PA5-86616 | WB in OE vs Control lysates, multi-epitope labeling | Fig S2-3 legends |
| antibody | Disabled homolog 1 | Dab1 | Rabbit IgG | Invitrogen | PA5-62538 | WB in OE vs Control lysates, multi-epitope labeling | Fig S2-3 legends |
| lysate | Disabled homolog 1 lysate | Dab1 OE lysate | HEK293T transient overexpression lysate | Novus Biologicals | NBL1-09709 | WB in OE vs Control lysates, multi-epitope labeling | Fig S2-3 legends |
| antibody | Postsynaptic density protein 95 | PSD95 | Mouse IgG1 | Invitrogen | MA1-0146 | WB in OE vs Control lysates, multi-epitope labeling | Fig S4 legend |
| antibody | Postsynaptic density protein 95 | PSD95 | Mouse IgG2a | Biolegend | 810401 | WB in OE vs Control lysates, multi-epitope labeling | Fig S4 legend |
| lysate | DLG4 (gene encoding PSD95) lysate | PSD95 OE lysate | HEK293T transient overexpression lysate | Novus Biologicals | NBL1-09911 | WB in OE vs Control lysates, multi-epitope labeling | Fig S4 legend |

Abbreviations: IHC, immunohistochemistry; HIER, heat induced epitope retrieval; ISH, *in situ* hybridization; FFPE, formalin-fixed paraffin embedded; WB, western blot; OE, overexpression.

**Table S4. False discovery rate adjusted p-values for manuscript figures ᵃ**

|  | **by Group ᵇ** | |  | **vs Braak stage ᶜ** | |  | **vs MMSE ᶜ** | |
| --- | --- | --- | --- | --- | --- | --- | --- | --- |
|  | **p-value** | **Sharpened** |  | **p-value** | **Sharpened** |  | **p-value** | **Sharpened** |
|  |  | **q-value** |  |  | **q-value** |  |  | **q-value** |
| **Figure 2.A. ErC** | | | | | | | | |
| ApoER2 | 0.002 | 0.003 |  |  |  |  |  |  |
| **Figure 2.C. Temporal Cortex** | | | | | | | | |
| ApoER2 | 0.002 | 0.002 |  |  |  |  |  |  |
| **Figure 3.B. ErC** | | | | | | | | |
| *Row 1* | | | | | | | | |
| pTau | <0.001 | 0.001 |  | <0.001 | 0.001 |  | <0.001 | 0.001 |
| Dab1 | 0.024 | 0.010 |  | 0.025 | 0.010 |  | 0.062 | 0.016 |
| pP85α | <0.001 | 0.001 |  | <0.001 | 0.001 |  | <0.001 | 0.001 |
| *Row 2* | | | | | | | | |
| pLIMK1 | 0.004 | 0.003 |  | 0.002 | 0.003 |  | <0.001 | 0.001 |
| pPSD95 | <0.001 | 0.001 |  | <0.001 | 0.001 |  | <0.001 | 0.001 |
| ApoJ | <0.001 | 0.001 |  | <0.001 | 0.001 |  | <0.001 | 0.001 |
| **Figure 5.C. ProS-CA1** | | | | | | | | |
| *Row 1* | | | | | | | | |
| Dab1 | <0.001 | 0.001 |  | <0.001 | 0.001 |  | <0.001 | 0.001 |
| pTau | <0.001 | 0.001 |  | <0.001 | 0.001 |  | <0.001 | 0.001 |
| pPSD95 | <0.001 | 0.001 |  | <0.001 | 0.001 |  | <0.001 | 0.001 |
| *Row 2* | | | | | | | | |
| pP85α | <0.001 | 0.001 |  | <0.001 | 0.001 |  | <0.001 | 0.001 |
| pLIMK1 | 0.122 | 0.027 |  | 0.009 | 0.005 |  | 0.043 | 0.013 |
| pDab1 | 0.009 | 0.005 |  | 0.003 | 0.003 |  | 0.006 | 0.004 |
| *Row 3* | | | | | | | | |
| ApoE | 0.011 | 0.006 |  | 0.018 | 0.008 |  | 0.052 | 0.015 |
| ApoJ | 0.048 | 0.014 |  | 0.001 | 0.002 |  | 0.005 | 0.004 |
| Reelin | 0.957 | 0.074 |  | 0.872 | 0.072 |  | 0.342 | 0.053 |
| **Figure 8.D. Temporal Cortex** | | | | | | | | |
| Dab1 | 0.005 | 0.004 |  | <0.001 | 0.002 |  | 0.043 | 0.013 |
|  |  |  |  | **vs Amyloid Plaques ᶜ** | |  | **vs PSD95 ᶜ** | |
|  |  |  |  | 0.018 | 0.008 |  | 0.001 | 0.002 |
| **Figure 9.C. LC and Raphe Nucleus** | | | | | | | | |
|  |  |  |  | **vs Braak Stage ᶜ** | |  |  |  |
| pTau | <0.001 | 0.001 |  | <0.001 | 0.001 |  |  |  |
| pPSD95 | 0.006 | 0.005 |  | 0.007 | 0.005 |  |  |  |
| ᵃ Based on the two-stage linear step-up procedure described by Benjamini, et al. [1, 2]. | | | | | | | | |
| ᵇ For Figure 2 & 9, p-values were determined using Wilcoxon rank-sum test by layers. For the other figures, p-values were determined with the Kruskal-Wallis equality-of-populations rank test. | | | | | | | | |
| ᶜ P-values were determined using Spearman's rank correlations test. | | | | | | | | |

**REFERENCES**

1 Anderson ML (2008) Multiple Inference and Gender Differences in the Effects of Early Intervention: A Reevaluation of the Abecedarian, Perry Preschool, and Early Training Projects. J Am Stat Assoc 103: 1481-1495 Doi 10.1198/016214508000000841

2 Benjamini Y, Krieger AM, Yekutieli D (2006) Adaptive Linear Step-up Procedures That Control the False Discovery Rate. Biometrika 93: 491-507

**II. Extended Figures**

| **Ext Fig** | **Title** |
| --- | --- |
| 3.1 | Laminar and cellular patterns of ApoER2 expression in ErC and neocortex |
| 3.2 | VLDLR and LRP1 lack the restricted expression observed for ApoER2 |
| 4.1 | Accumulation of ApoE and ApoJ in the ErC in sAD |
| 5.1 | Cytoarchitectural context for early Dab1 accumulation in the ErC |
| 6.1 | Multiple ApoER2-Dab1 components accumulate in the ProS-CA1 border region in sAD |
| 6.2 | Reelin and Dab1 accumulations in the hippocampus and ProS-CA1 border region in sAD |
| 9.1 | Dab1 accumulation within plaque-associated dystrophic axons in temporal neocortex in sAD |
| 9.2 | Neocortical Dab1 accumulation in a case with Aβ-ApoE plaques but no pTau pathology |
| 10.1 | Intra-neuronal Dab1 inclusions in locus coeruleus and raphe nucleus in sAD |
| 10.2 | Extracellular accumulations of ApoER2 ligands in locus coeruleus & raphe nucleus in sAD |


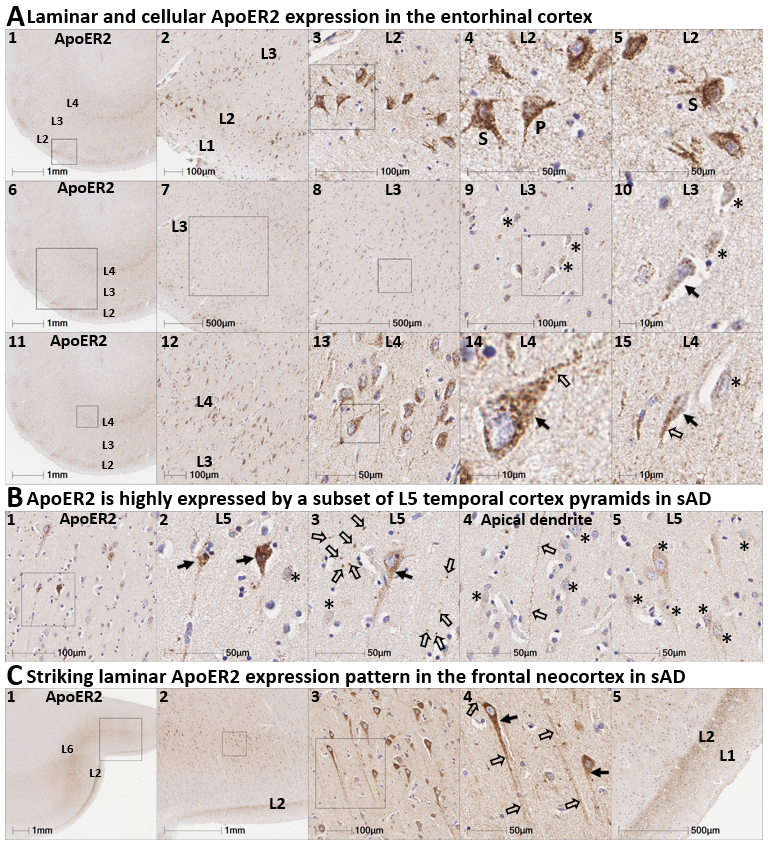


**Ext Fig 3.1. Laminar and cellular patterns of ApoER2 expression in the ErC and neocortex**

Panels **A-C** are coronal sections of the ErC (**A**), middle temporal gyrus (**B**), and frontal cortex (**C**) from one representative middle-aged non-AD control and two sAD cases, respectively. (**A**) ApoER2 is strongly expressed by stellate (demarcated by S in **A_4-5_**) and pyramidal neurons (demarcated by P in **A_4_**) in ErC L2. ApoER2 expression is lower or absent in L3 pyramids (**A_7-10_**) and strongly expressed by a subset of L4 pyramids and surrounding neurites (**A_11-15_**). Examples of L3 and L4 neurons with low ApoER2 expression are demarcated by an * in **A_9, 10 & 15_**. (**B**) ApoER2 is strongly expressed by a subset of neocortical L5 pyramids (solid arrows in **B_2-3_**) and their apical and basal dendritic projections (open arrows in **B_2-4_**); L5 neurons with low or absent ApoER2 expression are demarcated by an * in **A_4-5._** (**C**) In the frontal neocortex, ApoER2 is strongly expressed by a subset of L3 and L5 pyramidal neurons and their apical dendritic projections (open arrows in **B_4_**) and highly-ramified apical dendritic tufts (**C_2, 5_**) located in the vicinity of L2.


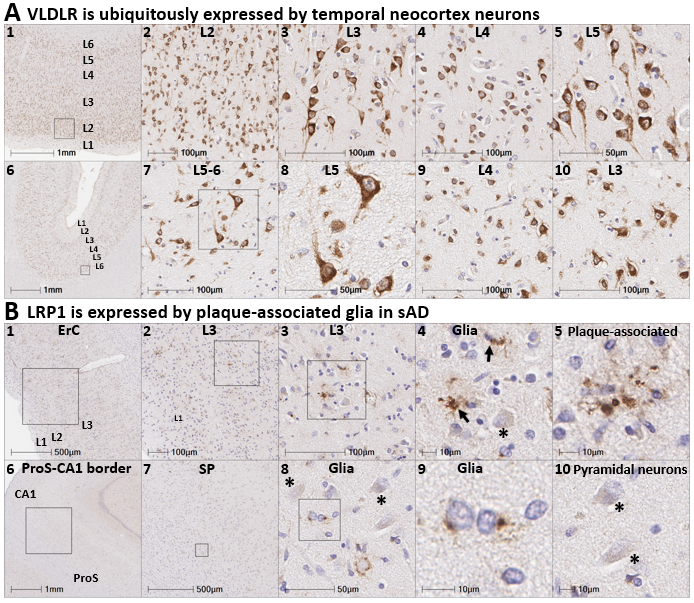


**Ext Fig 3.2. VLDLR and LRP1 lack the restricted expression observed for ApoER2**

Panels **A-B** are coronal sections of the temporal neocortex in a non-AD control (**A_1-5_**) and sAD case (**A_6-10_**), and the ErC (**B_1-5_**) and ProS-CA1 region from a sAD case (**B_6-10_**). The expression of VLDLR and LRP1 was less restricted than ApoER2 and neither closely matched the laminar and cellular distribution of NFT pathology. VLDLR was strongly and ubiquitously expressed by neurons in neocortical layers L2-L6 including neocortical L4 stellate neurons (**A_1-10_**) that are known to be resistant to NFT pathology. LRP1 was expressed by glia and some neurons, with prominent signals in glia surrounding NPs (**B_4, 8-10_**). Neurons with low or no LRP1 expression are demarcated by an * in **B_4, 8 & 10_**.


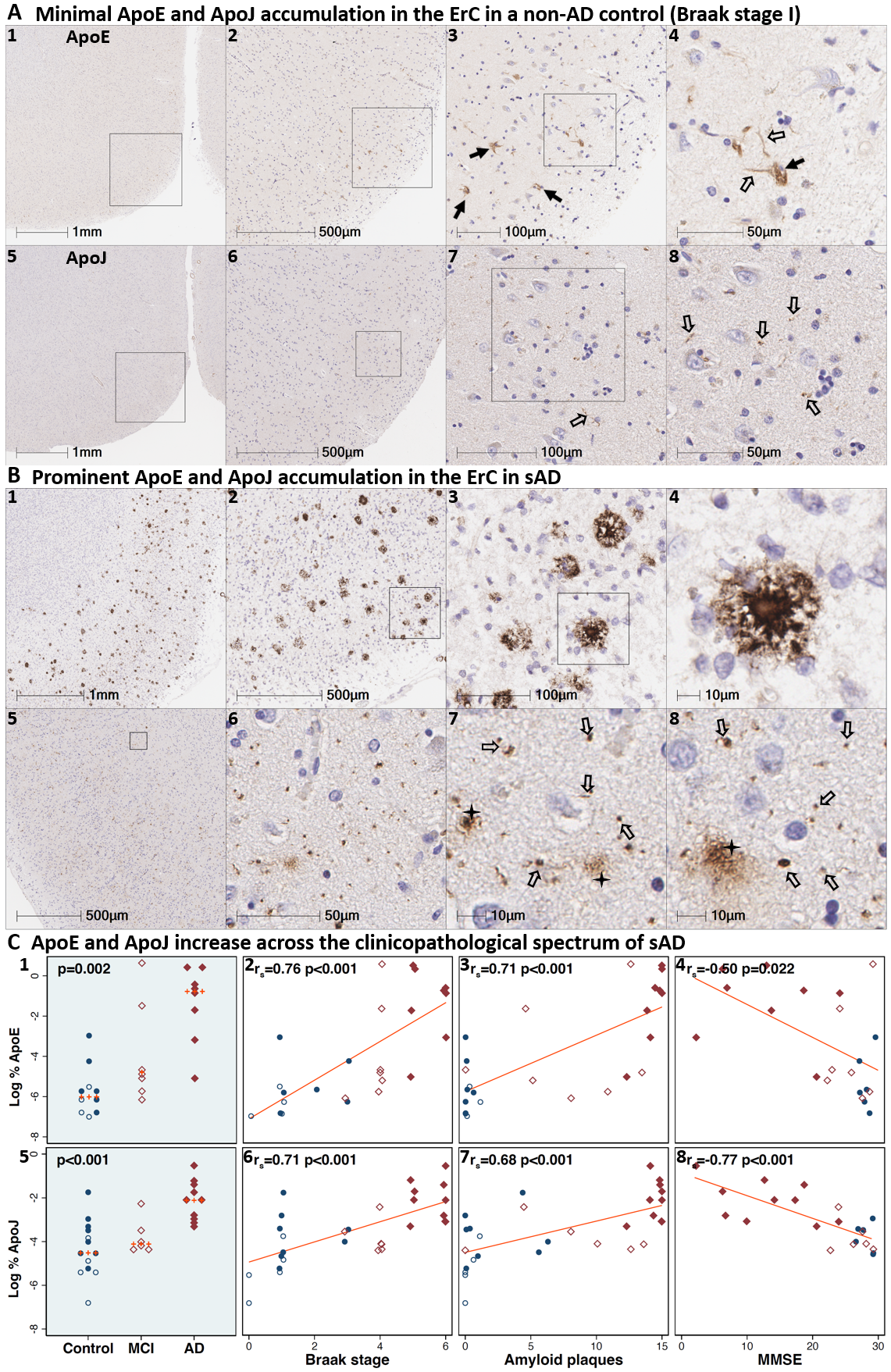


**Ext Fig 4.1** **Accumulation of ApoE and ApoJ in the ErC in sAD**

(**A**) Serial coronal sections of the ErC from a non-AD control case in the earliest stage of NFT pathology (Braak stage I) had only subtle ApoE (**A_1-4_**) and ApoJ (**A_5-8_**) expression. (**B**) Prominent extracellular accumulations of ApoE (**B_1-4_**) and ApoJ (**B_5-8_**) were observed in sAD cases. ApoE expression was most prominent in extracellular plaques. ApoJ was evident in both plaques (black stars) and discrete punctae within the neuropil (open arrows in **B_6-8_**). (**C**) ApoE and ApoJ were higher in sAD cases than controls, and positively correlated with histological progression and antemortem cognitive deficits.


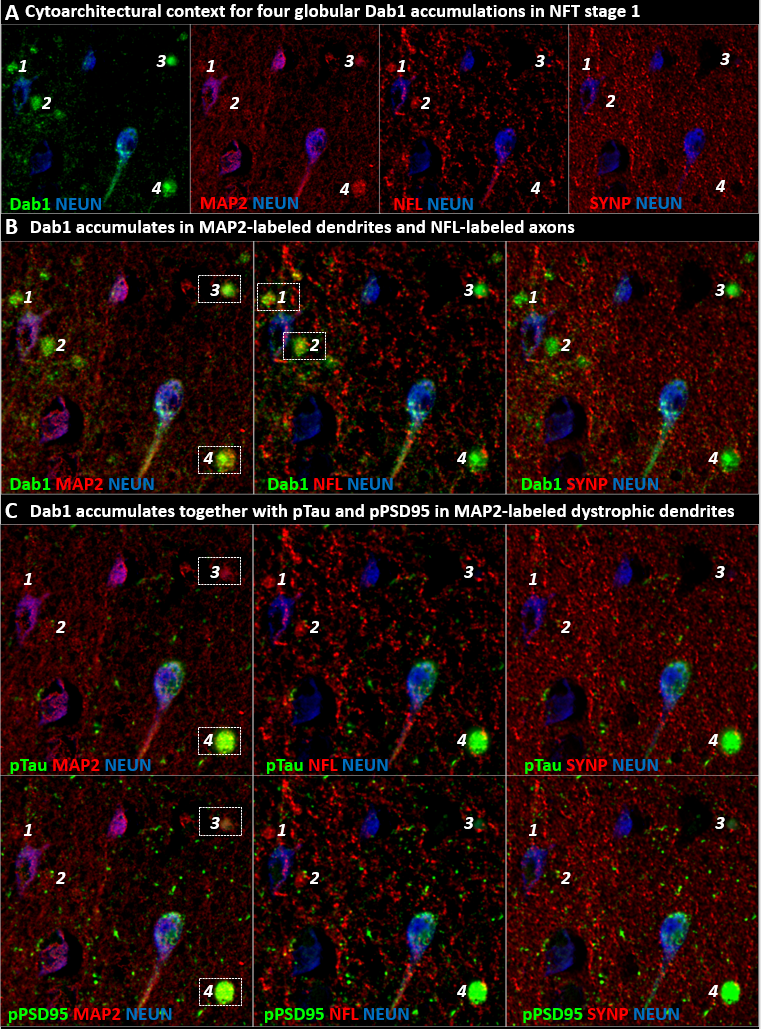


**Ext Fig 5.1 Cytoarchitectural context for early Dab1 accumulation in the ErC**

This figure provides cytoarchitectural context for Dab1 accumulation observed in the Braak stage I case shown in Fig 5. Panel **A** shows Dab1 accumulation in four prominent globular structures (**A_1-4_**) in the vicinity of affected ErC L2 stellate-shaped and pyramidal neurons. Panel **B** shows that two of these globular Dab1 accumulations colocalized with MAP2-labeled dystrophic dendrites and the other two colocalized with NFL-labeled dystrophic axons. Although these Dab1 structures accumulated in close proximity to synaptophysin (SYNP), no clear co-localization was evident. Panel **C** shows that pTau and pPSD95 expression overlapped with at least one the dendritic Dab1 accumulations but did not appear to colocalize with axonal Dab1.


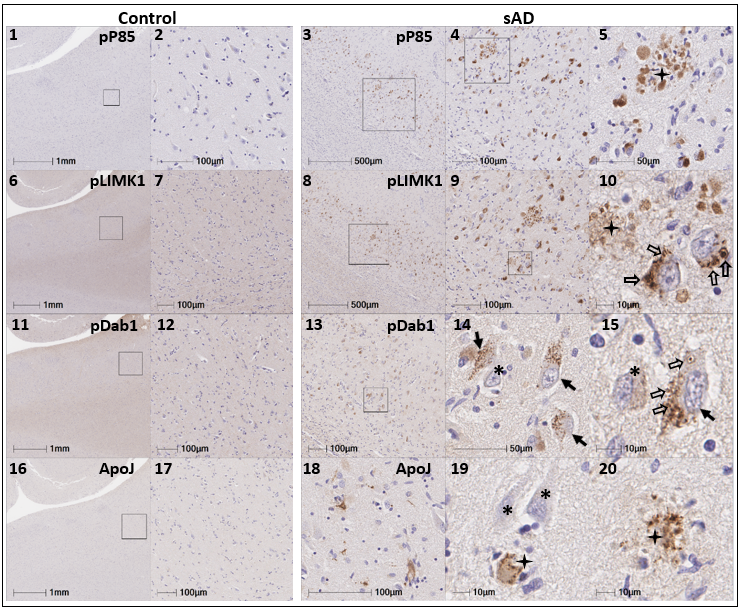


**Ext Fig 6.1. Multiple ApoER2-Dab1 components accumulate in the ProS-CA1 border region in sAD**

(**A**) Serial coronal sections of the ProS-CA1 region from representative non-AD control and sAD cases were probed with antibodies targeting ApoER2-Dab1 pathway components (see **Suppl Table 3**). In the non-sAD control (left column), IHC revealed very low expression of pP85α_Tyr607_, pLIMK1_Thr508_, and pDab1_Tyr220_, and ApoJ. By contrast, in sAD (right column), prominent accumulations of pP85α_Tyr607_, pLIMK1_Thr508_, and pDab1_Tyr220_ were observed within abnormal neurons (solid arrows) and in the vicinity of NPs (black stars). Open arrows in **A_10_** and **A_15_** designate granulovacuolar accumulations of pLIMK1_Thr508_ and pDab1_Tyr220_, respectively. ApoJ accumulated primarily in extracellular plaques (black stars). Neighboring neurons with little or no evidence of ApoER2-Dab1 component accumulation are designated with * (**A_15, 19_**).


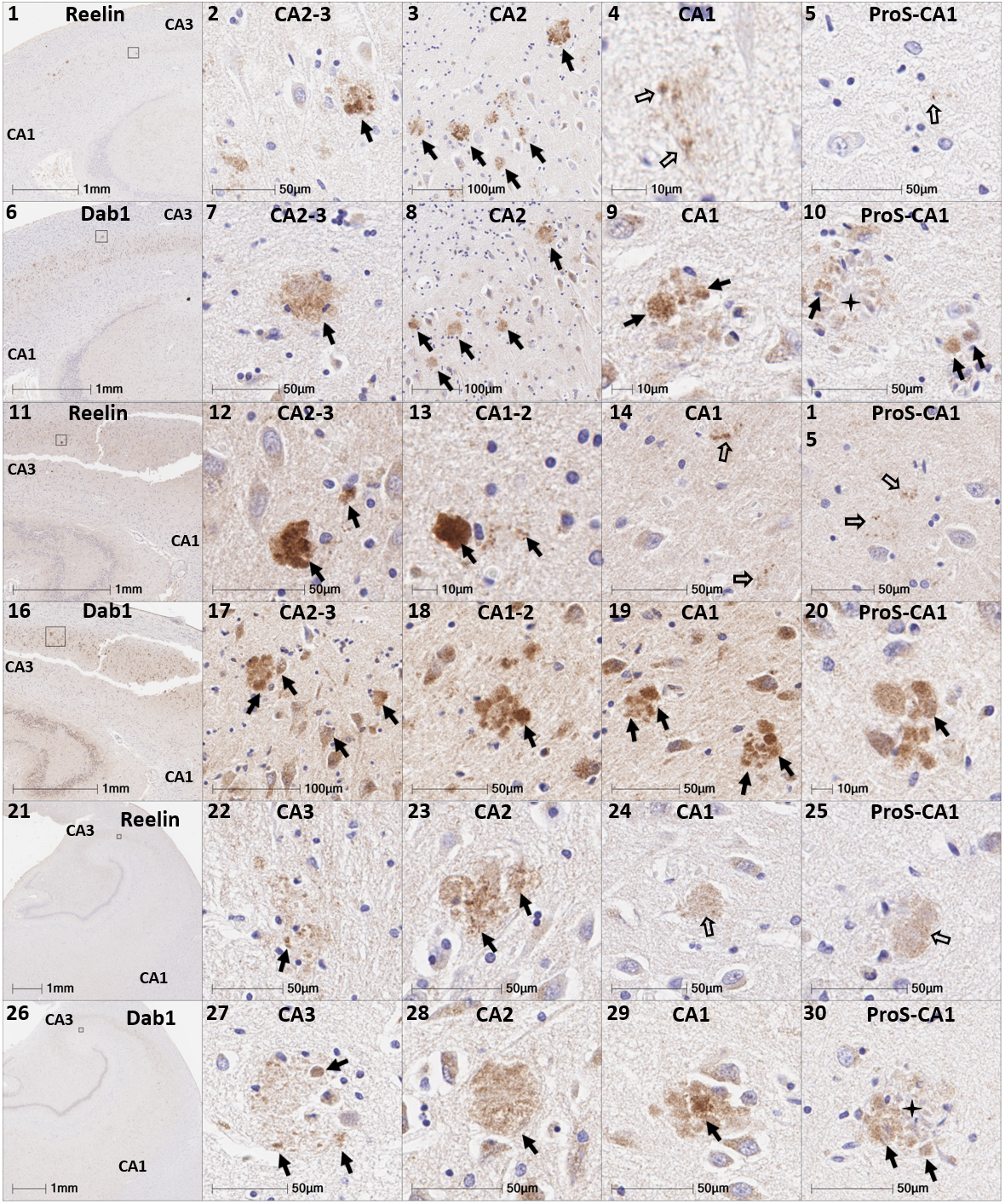


**Ext Fig 6.2 Reelin and Dab1 accumulations in the hippocampus and ProS-CA1 border region in sAD**

We previously reported that peri-plaque Reelin and Dab1 aggregates are evident in the CA2-3 region and the molecular layer of the dentate gyrus in a subset of sAD cases. Three such sAD cases with prominent Reelin and Dab1 accumulation are shown in Panels 1-10, 11-20, and 21-30, respectively. Although Reelin aggregates were prominent in the CA2-3 region (black arrows in Panels 1-3, 11-13, 21-23), they were subtle or absent in CA1 and the ProS-CA1 border region (open arrows in Panels 1-3, 11-13, 21-23). By contrast, Dab1 accumulations were more homogenously distributed throughout the cornu ammonis and the ProS-CA1 border region (black arrows in Panels 6-10, 16-20, 26-30).


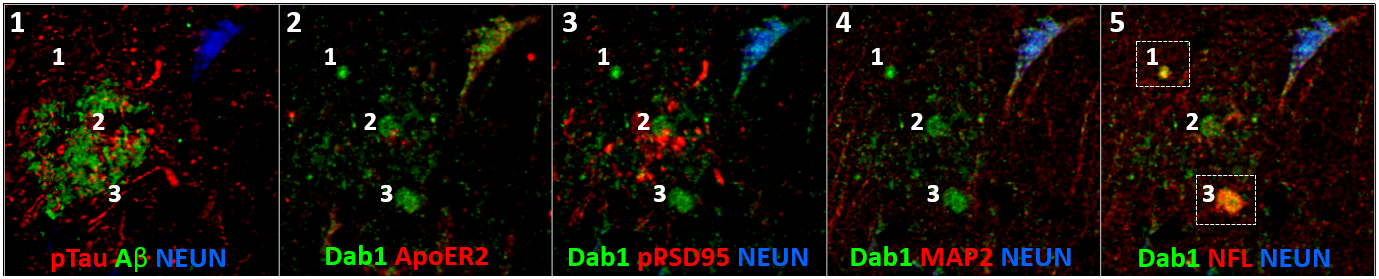


**Ext Fig 9.1 Dab1 accumulation within plaque-associated dystrophic axons in temporal neocortex in sAD** A coronal section of the temporal neocortex from a sAD case was probed with antibodies targeting Dab1, MAP2, NFL, NEUN, pTau, pPSD95, and Aβ (see **Suppl Table 3**). Dab1 accumulated within three prominent globular structures in the vicinity of an Aβ-labeled neuritic plaque. Two of these three globular Dab1-expressing structures colocalized with NFL-labeled dystrophic axons (demarcated by rectangles in panel 5). None of the three Dab1-expressing structures colocalized with MAP2, pTau or pPSD95.


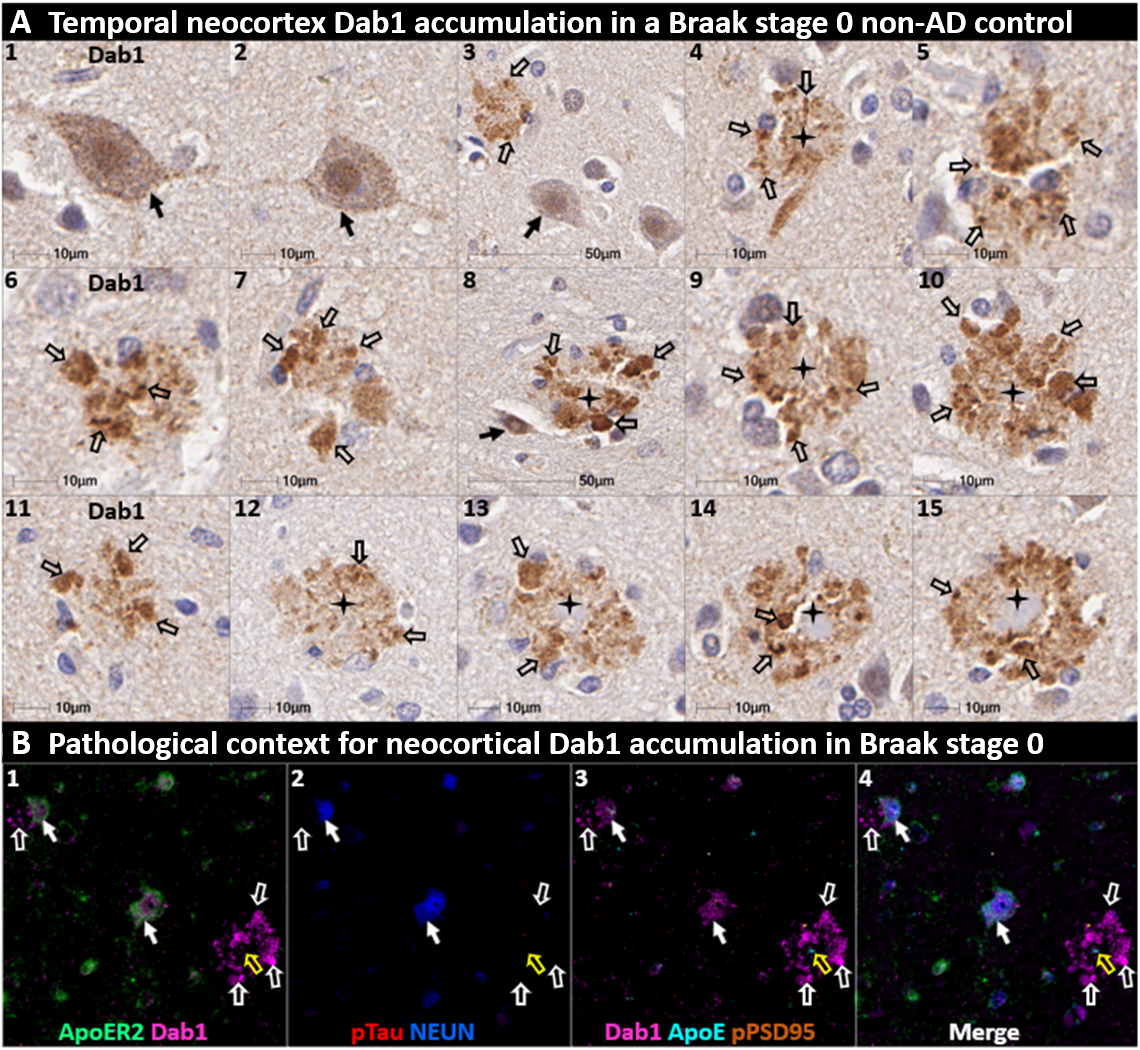


**Ext Fig 9.2 Neocortical Dab1 accumulation in a case with Aβ-ApoE plaques but no pTau pathology.**

Single-target IHC (**A_1-15_**) of temporal neocortex in a non-AD control with Aβ plaques but no overt pTau pathology (Thal phase 3, Braak stage 0) revealed globular Dab1 accumulations that were most prominent in L5 and L3. Dab1 accumulated in the vicinity of plaque-like structures (designated with stars in **A_4, 8-10_**_,_ **_12-15_**). Dab1 expression was evident in a subset of pyramidal neurons (solid arrows in **A_1-3, 8_**). pTau and pPSD95_Thr19_ were minimally expressed in serial sections. MP-IHC (**B_1-4_**) revealed that Dab1 accumulated in ApoER2-expressing neurons (white arrows) and that some globular Dab1 accumulations (open white arrows) were clustered around an ApoE-enriched central core (open yellow arrows).


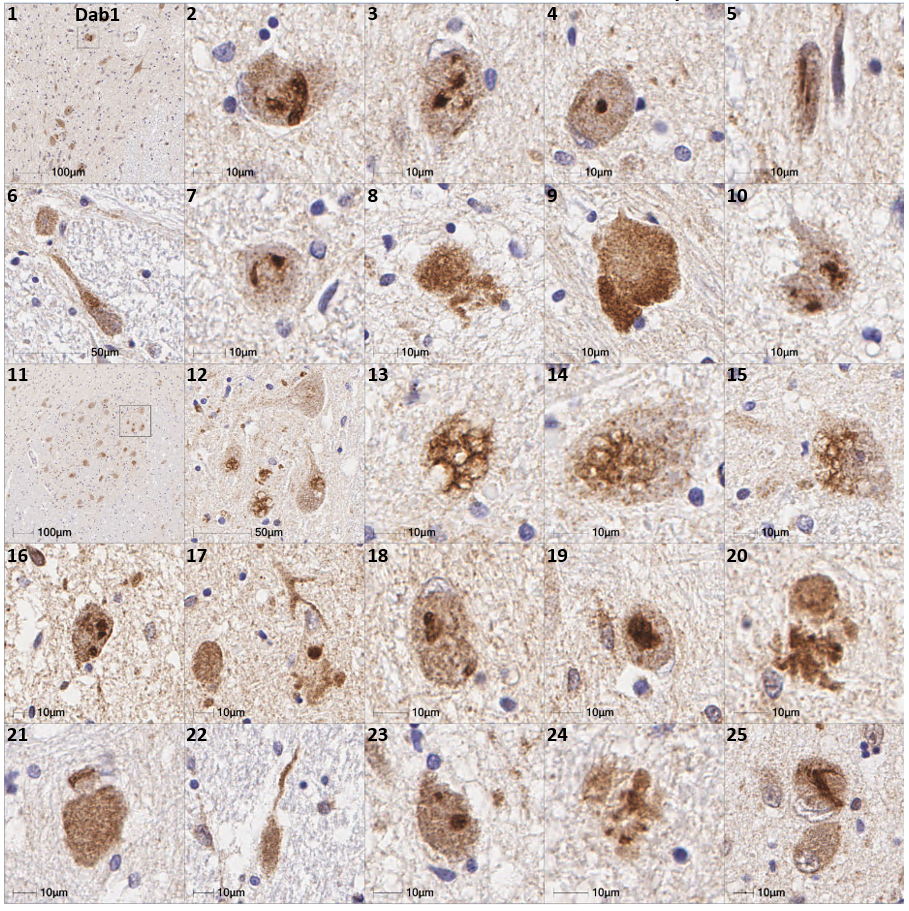


**Ext Fig 10.1. Intra-neuronal Dab1 inclusions in the locus coeruleus and raphe nucleus in sAD**

This figure depicts a variety of morphologies observed for intraneuronal accumulation of Dab1 in pontine LC-PC complex and raphe nucleus in four sAD cases.


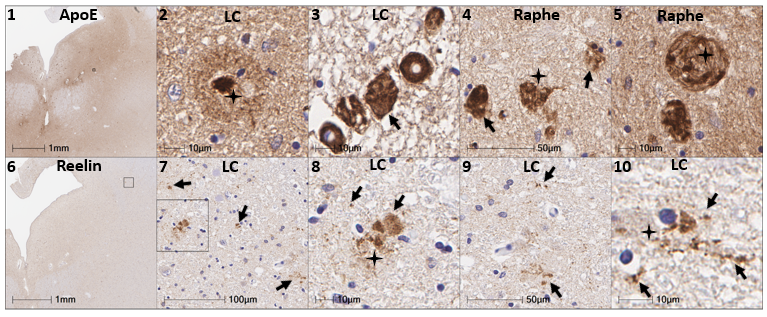


**Ext Fig 10.2. Extracellular accumulations of ApoER2 ligands in locus coeruleus & raphe nucleus in sAD**

Extracellular accumulations of ApoE were observed in many sAD cases (Panels 1-5). ApoE accumulated in both plaque-like structures (Panel 2) and the walls of blood vessels (Panel 3). Extracellular accumulations of Reelin were observed in a small subset of sAD cases (Panels 6-10) but were not evident in most cases.

**III. Supplementary Materials**

**and Methods**

**Neuropathological Assessments**

The neuropathological assessments and endpoints captured by BBDP are detailed in a previous publication [2]. Briefly, the Braak neurofibrillary stage (0 - VI) was determined using thick 40 – 80-micron sections stained with Gallyas, Campbell-Switzer and thioflavin S stains as originally defined by Braak and Braak [4]. Senile plaque density—including neuritic, cored, and diffuse plaques—was assessed in standard regions of the frontal, temporal, and parietal lobes, hippocampal CA1 region and entorhinal/transentorhinal region. Each region was assigned a semi-quantitative score of none, sparse, moderate and frequent and converted to numerical values 0 – 3, according to the CERAD templates [6]. Plaque total is the arithmetic sum of scores from these five regions ranged from 0 – 15. Neurofibrillary tangle density was assessed in the same five regions, with CERAD templates used to obtain semi-quantitative scores of none, sparse, moderate and frequent and these are converted to numerical values 0 – 3. Tangle total is the arithmetic sum of scores from these five regions ranged from 0 – 15. The NIA-Reagan [1] consensus recommendations were used for postmortem diagnosis of AD with high, intermediate and low referring to the likelihood that dementia, if present, is due to AD histopathology. AD was at a minimum defined as intermediate or high NIA-Reagan criteria. Mild Cognitive Impairment denoted the presence of this diagnosis at the time of death. A control designation is a participant without dementia or parkinsonism during life and without a major neuropathological diagnosis.

*Braak Stage:* describing topographical progression of neurofibrillary tangles, dystrophic neurites and neuropil threads, throughout transentorhinal and entorhinal areas, CA1 subfield of hippocampus, amygdala and cerebral neocortex. Evaluations were made, similarly as the original publication [4], in large (3 cm x 5 cm) thick (40 or 80 µm) sections stained with the Campbell-Switzer silver stain, Gallyas silver stain and Thioflavin S stains. Final judgment of tangle density is made on the basis of combined impression from all three stains. For three years, all cases were also stained with the AT8 antibody for phosphorylated tau protein. Note that the AT8 stain has been reported to give higher Braak stages as more neurites are apparent [3].

*Tangle Total:* Average neurofibrillary tangle density in the cortex of the frontal lobe, including superior, middle, and inferior frontal gyri; cortex of the temporal lobe; cortex of the parietal lobe; CA1 subfield of hippocampus; and entorhinal cortex. Tangle density scored according to the CERAD templates [6], as described for the Braak stage above.

*Plaque Total:* Average senile (amyloid) plaque density (all types of plaques considered together) in the cortex of frontal lobe, including superior, middle, and inferior frontal gyri; cortex of temporal lobe; cortex of parietal lobe; CA1 subfield of hippocampus; and entorhinal cortex. Plaque density scored according to CERAD templates [6], using large (3 cm x 5 cm) thick (40 or 80 µm) sections stained with Campbell-Switzer and Gallyas silver stains, and Thioflavin S stains. Validity and accuracy of this combination for estimating density of Aβ deposits established in BBDP laboratories through strong correlations with autoradiographic binding of Florbetapir (amyloid imaging ligand), with biochemical measures (ELISA) of Aβ in human cerebral cortex extracts and with quantitative measures (percentage of section area occupied) of an immunohistochemical stain for Aβ [2].

*Neuritic Plaque Density:* Greatest neuritic plaque density observed across frontal, temporal and parietal cortex regions, scored according to CERAD templates [6]. Evaluations made in large (3 cm x 5 cm) thick (40 or 80 µm) sections stained with the Campbell-Switzer silver stain, Gallyas silver stain and Thioflavin S stains. Final judgment of plaque density made on the basis of the combined impression from all three stains [2].

**Cognitive Assessments**

The cognitive examinations and endpoints captured by BBDP are detailed in a previous publication [2] and are summarized below.

*MMSE Test Score:* Folstein Mini Mental State Examination score (0-30) obtained most proximal to death; includes MMSE scores obtained through BBDP research clinical visits and by review of private medical records [5].

*CDR Sum:* Sum of Boxes (subsection) of the Clinical Dementia Rating (CDR) Scale. CDR is widely used for staging dementia severity [7].

*FAST Score:* FAST is a functional assessment based both on caregiver report and clinician’s observations: 1 = normal; 2 = subjective (only) report of forgetfulness or work difficulties; 3 = observed early executive dysfunction; 4 = definite memory and/or executive dysfunction; 5 = some decline in basic activities of daily living (ADLs); 6 (a-e) definite decline in basic ADLs, with the final stage (e) being fecal incontinence; 7 (a-e) progressive loss of speech and motor abilities, with the final stage (e) being loss of the ability to hold up the head independently [8].

*Figure Recall Score:* Subject is asked to copy three simple figures, and after delay, is asked to reproduce the figures from memory. The score is the number correctly reproduced.

*Rey Auditory Verbal Learning Test (AVLT):* evaluates short-term auditory-verbal memory, rate of learning, learning strategies, retroactive, and proactive interference, presence of confabulation, of confusion in memory processes, retention of information, and differences between learning and retrieval. Participants are given list of 15 unrelated words repeated over five trials and asked to repeat as many words as possible. After the five trials, the number of recalled words is summed as “AVLT Total Learning Score” (0-75 scale). After the fifth learning trial, another list of 15 unrelated words is provided. The participant recalls as many words as possible from this distracter list. After a brief delay, the participant is asked to repeat the original list of 15 words (AVLT A6) [9, 10].

**REFERENCES**

1 (1997) Consensus recommendations for the postmortem diagnosis of Alzheimer's disease. The National Institute on Aging, and Reagan Institute Working Group on Diagnostic Criteria for the Neuropathological Assessment of Alzheimer's Disease. Neurobiol Aging 18: S1-2

2 Beach TG, Adler CH, Sue LI, Serrano G, Shill HA, Walker DG, Lue L, Roher AE, Dugger BN, Maarouf Cet al (2015) Arizona Study of Aging and Neurodegenerative Disorders and Brain and Body Donation Program. Neuropathology 35: 354-389 Doi 10.1111/neup.12189

3 Braak H, Alafuzoff I, Arzberger T, Kretzschmar H, Del Tredici K (2006) Staging of Alzheimer disease-associated neurofibrillary pathology using paraffin sections and immunocytochemistry. Acta Neuropathol 112: 389-404 Doi 10.1007/s00401-006-0127-z

4 Braak H, Braak E (1991) Neuropathological stageing of Alzheimer-related changes. Acta Neuropathol 82: 239-259 Doi 10.1007/BF00308809

5 Folstein MF, Folstein SE, McHugh PR (1975) "Mini-mental state". A practical method for grading the cognitive state of patients for the clinician. J Psychiatr Res 12: 189-198 Doi 10.1016/0022-3956(75)90026-6

6 Mirra SS, Heyman A, McKeel D, Sumi SM, Crain BJ, Brownlee LM, Vogel FS, Hughes JP, van Belle G, Berg L (1991) The Consortium to Establish a Registry for Alzheimer's Disease (CERAD). Part II. Standardization of the neuropathologic assessment of Alzheimer's disease. Neurology 41: 479-486 Doi 10.1212/wnl.41.4.479

7 O'Bryant SE, Waring SC, Cullum CM, Hall J, Lacritz L, Massman PJ, Lupo PJ, Reisch JS, Doody R, Texas Alzheimer's Research C (2008) Staging dementia using Clinical Dementia Rating Scale Sum of Boxes scores: a Texas Alzheimer's research consortium study. Arch Neurol 65: 1091-1095 Doi 10.1001/archneur.65.8.1091

8 Reisberg B (1988) Functional assessment staging (FAST). Psychopharmacol Bull 24: 653-659

9 Rey A (1958) L'examen clinique en psychologie. [The clinical examination in psychology.]. Presses Universitaries De France, City

10 Ricci M, Graef S, Blundo C, Miller LA (2012) Using the Rey Auditory Verbal Learning Test (RAVLT) to differentiate alzheimer's dementia and behavioural variant fronto-temporal dementia. Clin Neuropsychol 26: 926-941 Doi 10.1080/13854046.2012.704073

**Antibody selection and validation**

As shown in **Suppl Table 3**, all antibodies used for IHC in this study have previously been used for IHC in human FFPE specimens, and those targeting core ApoER2-Dab1 pathway components have been used specifically for IHC in human brain FFPE specimens in published manuscripts. Data supporting the validation of these antibodies (i.e., Western blot, RNA-protein co-detection, multi-epitope immunolabeling with two or more antibodies targeting the same protein) was published in the supplementary materials and methods section of a 2022 manuscript (Ramsden, Keyes et al, J Alzheimers Dis 87: 1251-1290) and is summarized in **Suppl Table 3**. For the present study, we performed additional automated Western immunoblotting experiments comparing antibody detection of lysates generated from HEK293T cells transfected to transiently overexpress human ApoER2, Dab1, and PSD95 proteins or empty vector transfected HEK293T cells (see **Figs S1-S4** below).


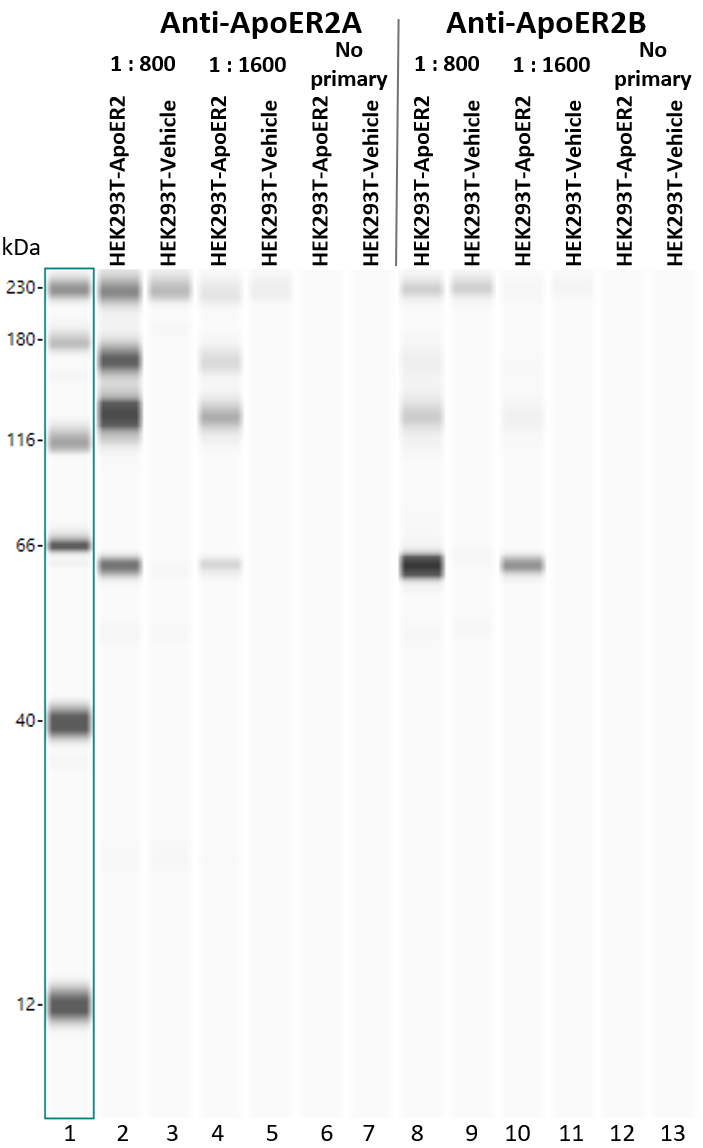


**Fig S1. Immunodetection with ApoER2 antibodies and overexpression or empty vector control cell lysates**

Automated Western immunoblotting was performed with a capillary nano-immunoassay platform (Wes^TM^) using two rabbit IgG antibodies targeting different ApoER2 domains and lysates generated from HEK293T cells that were transfected to transiently overexpress human *LRP8* (gene encoding ApoER2 [Novus, nbp2-06973]) (Lanes 2, 4, 6, 8, 10, 12) or empty vector transfected HEK293T cells (Lanes 3, 5, 7, 9, 11, 13). The first lane represents the molecular mass marker in kDa. Anti-rabbit (Novus DM-001) secondary HRP-conjugated antibody was loaded according to manufacturer instructions. Compass exposure setting 1 (1 second) was applied for visualization of bands. Anti-ApoER2A and anti-ApoER2B target the midchain region of ApoER2 within the beta-propeller domain and the NPXY domain within the cytoplasmic tail of ApoER2, respectively. Using primary antibody dilutions of 1 to 800 (Lanes 2-3 & 8-9), 1 to 1600 (Lanes 4-5 & 10-11) showed bands that are consistent with each other and with the molecular weights specified in commercial lysates. Signals were weaker or absent in vehicle transfected controls and secondary only controls with primary antibodies omitted (Lanes 6-7 & 12-13). Western blots are representative of experiments performed three times.


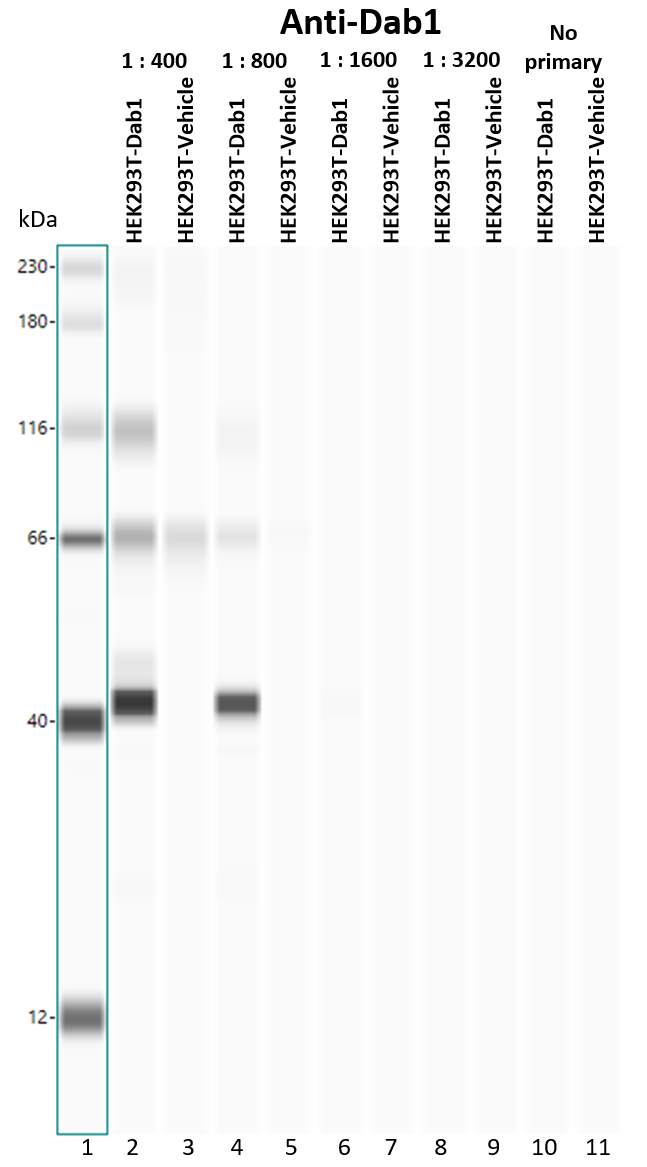


**Fig S2. Immunodetection with Dab1 antibody and overexpression or empty vector control cell lysates**

Automated Western immunoblotting was performed with a capillary nano-immunoassay platform (Wes^TM^) using a rabbit antibody targeting Dab1 (Invitrogen, PA5-86617) and lysates generated from HEK293T cells that were transfected to transiently overexpress human *DAB1* (gene encoding the disabled homolog-1 (Dab1) protein [Novus, nbl1-09709]) (Lanes 2, 4, 6, 8, 10) or empty vector transfected HEK293T cells (Lanes 3, 5, 7, 9, 11). The first lane represents the molecular mass marker in kDa. An anti-rabbit secondary HRP-conjugated antibody (Novus DM-001) was loaded according to manufacturer instructions. Compass exposure setting 1 (1 second) was applied for visualization of bands. Using primary antibody dilutions of 1 to 400 (Lanes 2-3), 1 to 800 (Lanes 4-5), and 1 to 1600 (Lanes 6-7) and 1 to 3200 (Lanes 8-9) and with the primary antibody omitted (Lanes 10-11) showed bands that are consistent with molecular weights specified in commercial lysates and weaker or no signals in vehicle transfected controls. Western blots are representative of experiments performed three times.


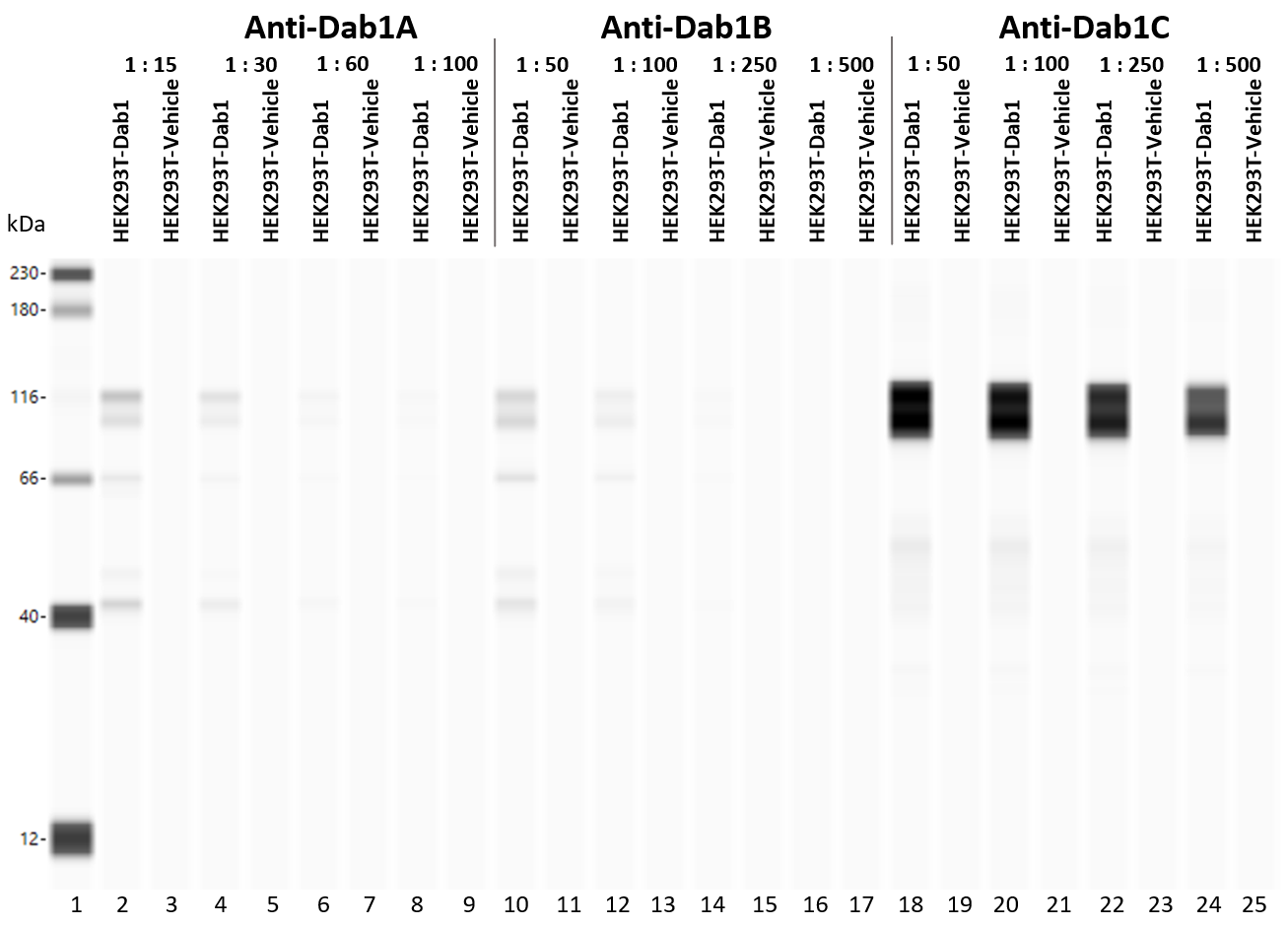


**Fig S3. Immunodetection with Dab1 antibodies and overexpression or empty vector control cell lysates**

Automated Western immunoblotting was performed with a capillary nano-immunoassay platform (Wes^TM^) using three rabbit antibodies targeting different Dab1 domains and lysates generated from HEK293T cells that were transfected to transiently overexpress human *DAB1* (gene encoding the disabled homolog-1 (Dab1) protein [Novus, nbl1-09709]) (Lanes 2, 4, 6, 8, 10, 12, 14, 16, 18, 20, 22, 24) or empty vector transfected HEK293T cells (Lanes 3, 5, 7, 9, 11, 13, 15, 17, 19, 21, 23). The first lane represents the molecular mass marker in kDa. An anti-rabbit secondary HRP-conjugated antibody (Novus DM-001) was loaded according to manufacturer instructions. Compass high dynamic range setting was applied for visualization of bands. Anti-Dab1A (Invitrogen, PA5-86617 Rabbit IgG), anti-Dab1B (Invitrogen, PA5-86616 Rabbit IgG), and anti-Dab1C (Invitrogen, PA5-62538 Rabbit IgG) target two adjacent regions within the Dab1 SH2 domain, and amino acids 517-585 within the extreme C-terminal region of Dab1, respectively. Immunodetection showed bands that are consistent with each other and with the molecular weights specified in commercial lysates and weaker or no signals in vehicle transfected controls. Western blots are representative of experiments performed three times.


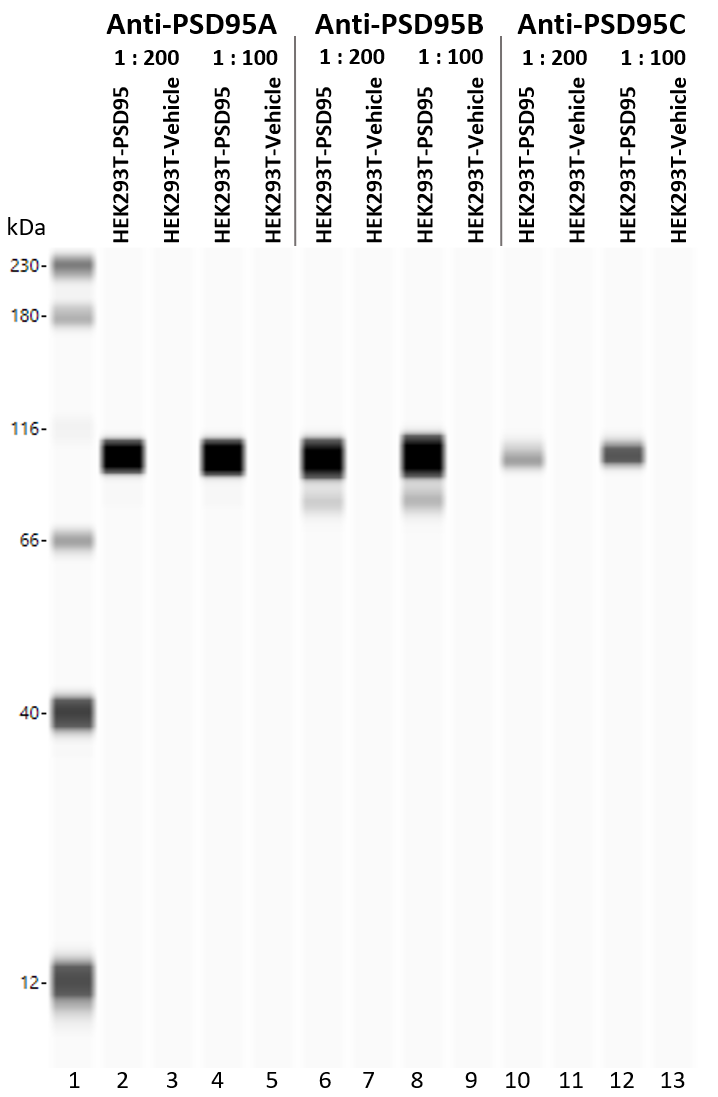


**Fig S4. Immunodetection with PSD95 antibodies and overexpression or empty vector control cell lysates**

Automated Western immunoblotting was performed with a capillary nano-immunoassay platform (Wes^TM^) using three rabbit antibodies targeting different PSD95 domains and lysates generated from HEK293T cells that were transfected to transiently overexpress human *DLG4* (gene encoding PSD95 protein [Novus, nbl1-09911]) (Lanes 2, 4, 6, 8, 10, 12) or empty vector transfected HEK293T cells (Lanes 3, 5, 7, 9, 11, 13). The first lane represents the molecular mass marker in kDa. Isotype matched anti-mouse (Novus DM-002) and anti-rabbit (Novus DM-001) secondary HRP-conjugated antibodies were loaded according to manufacturer instructions. Compass exposure setting 2 (2 seconds) was applied for visualization of bands. Anti-PSD95A (Invitrogen, MA1-0146 Mouse IgG1), anti-PSD95B (Biolegend 810401, Mouse IgG2a), and anti-PSD95C (Millipore ABN998, Rabbit IgG) target the full-length PSD95 protein, amino acids 77-299 within the PDZ-1 and PDZ-2 domains of PSD95, and Thr19-phosphorylated epitope within the extreme N-terminus region of PSD95, respectively. Using primary antibody dilutions of 1 to 200 (Lanes 2-3 & 6-7 & 10-11) and 1 to 100 (Lanes 4-5 & 8-9 & 12-13) showed bands that are consistent with each other and with the molecular weights specified in commercial lysates. Signals were weaker or absent in vehicle transfected controls. Western blots are representative of experiments performed three times.

**Immunohistochemical Marker Quantitation**

Stain positive area as a percentage of each annotated region was quantified using HALO 3.5 image analysis software (Indica Labs, Corrales, NM) HALO Area Quantification v2.4.2 module (**Fig S5A**). Plaque-associated objects per mm^2^ within each annotated region were identified and quantified using the HALO Object Colocalization v2.1.5 module with classifier function enabled (**Fig S5B**).


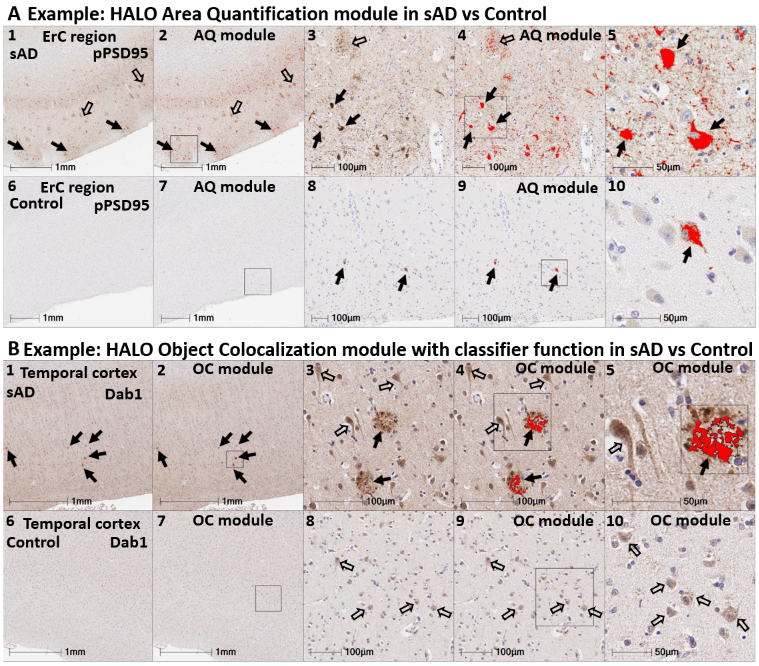


**Fig S5. Representative examples of HALO modules used for detection of IHC markers**

Panels **A_1-10_** illustrate the application of the HALO Area Quantification (AQ) module (v2.4.2) in the ErC of a Braak stage VI sAD case (**A_1-5_**) and a non-AD control (**A_6-10_**). Thr19-pPSD95 was strongly expressed in abnormal neurons (black arrows) and neuritic plaques (open arrows) in sAD with only minimal detection in the non-AD control (lack of brown stain in panels **A_6-10_**). Thr19-pPSD95-positive structures that were identified by the AQ module are depicted in red in **A_2, 4-5_** and **A_7, 9-10_**. Panels **B_1-10_** illustrate the application of the HALO Object Colocalization (OC) v2.1.5 module. As shown in **Figs 6-10** of the main paper, Dab1 is strongly expressed as globular structures in the vicinity of neuritic plaques and is also typically expressed in neurons. The OC module selectively identified plaque-associated aggregates (black arrows in panels **B_1-5_**) without labeling neurons (open arrows in in panels **B_1-10_**). A representative example of localized Dab1-positive aggregates in the temporal neocortex of one Braak stage VI sAD case is shown in panels **B_1-5_**, with aggregates detected by the OC module depicted in red in panels **B_2_** and **B_4-5_**. The single box shown in panel **B_5_** indicates that the structure in red is counted as one object. The lack of red color and lack of a box around the adjacent neurons in **B_4-5_** indicate that the module selectively identifies and counts plaque-associated Dab1 aggregates but not Dab1 that is present in neurons. The lack of red in panels **B_7_** and **B_9-10_** indicate that no plaque associated Dab1 aggregates were detected.
